# Causal roles of educational duration in bone mineral density and risk factors for osteoporosis: a Mendelian randomization study

**DOI:** 10.1186/s12891-024-07428-8

**Published:** 2024-05-02

**Authors:** Yujun Qin, Xia Yang, Zong Ning

**Affiliations:** 1 Department of General Practice, The First Affiliated Hospital of Guangxi Medical University, Guangxi Zhuang Autonomous Region, Nanning, P.R. China; 2 The People’s Hospital of Hechi, Guangxi Zhuang Autonomous Region, Hechi, P.R. China

**Keywords:** Educational duration, Bone mineral density, Osteoporosis, Risk factors, Mendelian randomization

## Abstract

**Background:**

Educational duration might play a vital role in preventing the occurrence and development of osteoporosis(OP).

**Purpose:**

To assess the causal effect of educational duration on bone mineral density(BMD) and risk factors for OP by Mendelian randomization(MR) study.

**Methods:**

The causal relationship was analyzed using data from genome-wide association study(GWAS). Inverse variance weighting (IVW) was used as the main analysis method. Horizontal pleiotropy was identified by MR-Egger intercept test, MR pleiotropy residual sum and outlier (MR-PRESSO) test. The leave-one-out method was used as a sensitivity analysis.

**Results:**

The IVW results indicated that there was a positive causal relationship between educational duration and BMD (OR = 1.012, 95%CI:1.003–1.022), physical activity(PA) (OR = 1.156, 95%CI:1.032–1.295), calcium consumption (OR = 1.004, 95%CI:1.002–1.005), and coffee intake (OR = 1.019, 95%CI:1.014–1.024). There was a negative association between whole body fat mass (OR = 0.950, 95%CI:0.939–0.961), time for vigorous PA (OR = 0.955, 95%CI:0.939–0.972), sunbath (OR = 0.987, 95%CI:0.986–0.989), salt consumption (OR = 0.965, 95%CI:0.959–0.971), fizzy drink intake (OR = 0.985, 95%CI:0.978–0.992), smoking (OR = 0.969, 95%CI:0.964–0.975), and falling risk (OR = 0.976, 95%CI:0.965–0.987). There was no significant association between educational duration and lean mass, time for light-to-moderate PA, milk intake, and alcohol intake. Horizontal pleiotropy was absent in this study. The results were robust under sensitivity analyses.

**Conclusion:**

A longer educational duration was causally linked with increased BMD. No causal relationship had been found between educational duration and lean mass, time for light-to-moderate PA, milk intake, and alcohol consumption as risk factors for osteoporosis.

**Supplementary Information:**

The online version contains supplementary material available at 10.1186/s12891-024-07428-8.

## Introduction

Osteoporosis(OP) is a widely prevalent skeletal disorder that poses a significant public health concern due to its increasing incidence worldwide [1]. Exploring the risk factors and maintaining bone mineral density(BMD) are crucial for its prevention and treatment. Therefore, identifying and addressing the risk factors can reduce the risk of developing OP and improve overall skeletal health [2]. OP diagnosis and treatment vary by region and urban/rural areas. Risk factors include low physical activity(PA), calcium deficiency, lack of sunlight exposure, high sodium intake, dietary imbalances, low weight, falls, smoking, alcohol, excessive intake of coffee and fizzy drink [3]. Taking preventative measures is crucial. In recent years, attention has been drawn to the potential correlation between educational level and the risk factors for OP. The impact of educational level on individual health outcomes has been widely discussed and researched. It is considered a comprehensive measure of an individual’s knowledge and socioeconomic status [4]. Furthermore, research has indicated a positive correlation between bone mineral density and educational attainment [5]. Higher education levels are linked to better BMD due to healthier lifestyles—more PA [6], less smoking, and healthier dietary habits [7]. Educational interventions can promote healthier BMD levels. However, a study revealed a lack of awareness regarding BMD and related intervention measures [8]. Currently, there is no universally agreed upon consensus regarding the correlation between educational attainment and BMD, or the risk factors associated with OP. It is imperative to conduct further research to understand the potential impact of educational level on BMD, body composition, and lifestyles, in order to provide scientific evidence for this relationship. Mendelian randomization (MR) has become a powerful approach for estimating causal relationships and enhancing causal inference. By utilizing genetic variants as instrumental variables (IVs), MR can effectively reduce bias in causal analysis of exposure and outcomes. As a result, MR has been extensively utilized in various fields to explore the causal relationships between different factors [9]. Our hypothesis is that if the duration of education is related to osteoporosis, then it is possible that a longer duration of education could lead to a decrease in the risk of developing osteoporosis by influencing bone density and related risk factors. Therefore, we aim to explore the correlation between the duration of education as an exposure factor and the outcome of bone density and related risk factors for osteoporosis using MR analysis. By examining the causal relationship between exposure and outcome, we hope to determine whether the duration of education is indeed associated with osteoporosis. The purpose of this article is to raise awareness and emphasize the importance of osteoporosis, while promoting education about its risks and preventive measures. It is crucial to integrate knowledge about osteoporosis and its preventive measures into early education systems, in order to reduce the increased risk of developing osteoporosis associated with varying levels of education.

## Methods

### GWAS data sources

The data used in this study were obtained from the IEU GWAS database at the University of Bristol (https://gwasmrcieu.ac.uk). The exposure variable of educational duration data was obtained from a study by Loh PR published in 2018 [10], which included 461,457 samples and 11,972,619 single nucleotide polymorphisms (SNPs). For the outcome variables, BMD data was obtained from a study by Mbatchou J published in 2021 [11]. Data on whole-body fat mass, sunbath, time for light PA, time for moderate PA, time for vigorous PA, milk intake, calcium consumption, salt consumption, coffee and fizzy drink intake were obtained from a study by Ben Elsworth published in 2018 [12]. Whole-body lean mass data was obtained from a study by Medina-Gomez C published in 2017 [13]. PA data was obtained from a study by Hanscombe KB published in 2021 [14]. Falling risk data was obtained from a study by Trajanoska K published in 2020 [15]. Smoking data was obtained from the study by Loh PR published in 2018 [10]. Alcohol consumption data was obtained from a study by Howe LJ published in 2022 [16]. Detailed information is given in Table 1.

**Table 1:** Details of studies and datasets used for analyses

Exposure /Outcomes	Year	Population	Sample size	Number of SNPs	Author	PMID/note	GWAS ID
Educational years	2018	European	461,457	11,972,619	Loh PR	29892013	ebi-a-GCST90029013
Bone mineral density	2021	European	365,403	10,783,906	Mbatchou J	34017140	ebi-a-GCST90014022
Whole body fat mass	2018	European	454,137	9,851,867	Ben Elsworth	23100	ukb-b-19393
Whole body lean mass	2017	European	331,291	10,894,596	Neale	HG19/GRCh37	ukb-a-266
Sunbath	2018	European	436,185	9,851,867	Ben Elsworth	2277	ukb-b-4943
Physical activity	2021	European	89,683	8,669,219	Hanscombe KB	34753499	ebi-a-GCST90093322
Time for light physical activity	2018	European	64,949	9,851,867	Ben Elsworth	104920	ukb-b-8865
Time for moderate physical activity	2018	European	64,949	9,851,867	Ben Elsworth	104910	ukb-b-2115
Time for vigorous physical activity	2018	European	64,949	9,851,867	Ben Elsworth	104900	ukb-b-13702
Falling risk	2020	European	451,179	7,720,247	Trajanoska K	32999390	ebi-a-GCST90012857
Salt consumption	2018	European	462,630	9,851,867	Ben Elsworth	1478	ukb-b-8121
Smoking	2018	European	468,170	11,973,425	Loh PR	29892013	ebi-a-GCST90029014
Coffee intake	2018	European	428,860	9,851,867	Ben Elsworth	1498	ukb-b-5237
Fizzy drink intake	2018	European	64,949	9,851,867	Ben Elsworth	100170	ukb-b-2832
Calcium consumption	2018	European	461,384	9,851,867	Ben Elsworth	6179#3	ukb-b-7043
Milk intake	2018	European	64,943	9,851,867	Ben Elsworth	100520	ukb-b-2966
Alcohol intake	2022	European	83,626	7,914,362	Howe LJ	EFO:0007878	ieu-b-4834

*SNPs* single nucleotide polymorphisms, *GWAS* genome-wide association study, *ID* Identity document

### Instrumental variables selection process

Genome-wide significant SNPs, which were independent of and highly correlated with the exposure variable and outcome variable, were selected as IVs. The genome-wide information from the UK Biobank’s whole-genome sequencing project was used as a reference [17]. The genome-wide significance threshold for educational duration was set at *p* < 5 × 10^–8^. The linkage disequilibrium (R^2^) threshold was set at 0.001, and the genetic distance was set at 10 MB. IVs without any linkage effects were selected from the data. Then, IVs that were significant predictors of the outcome variable (*P* < 0.05) were excluded from the selected IVs. For missed SNPs in the outcome GWAS dataset, proxies were identified at the cutoff of R^2^ > 0.8. If no suitable proxy was available, SNPs were discarded. The F-statistic was used to verify the strength of IVs, using the following formula: R^2^ × (N − 2)/(1 − R^2^). Here, R^2^ indicated the proportion of variance in educational duration explained by a given SNP and N indicated sample size. More specifically, R^2^ was calculated with the following formula: R^2^ = [2 × Beta^2^ × (1 − EAF) × EAF]/[2 × Beta^2^ × (1 − EAF) × EAF + 2 × SE^2^ × N × (1 − EAF) × EAF]. Here, Beta indicated the genetic effect of SNP on educational duration, EAF was effect allele frequency, SE was standard error, and N was sample size; only strong IVs (F-statistic > 10) for each of the exposures of interest were retained. Fourth, we excluded ambiguous and palindromic SNPs (minor allele frequency > 0.42) for which the effect cannot be corrected in the harmonizing process. The MR-pleiotropy residual sum and outlier (MR-PRESSO) test was conducted to discard SNPs with potential pleiotropy.

### Mendelian randomization

To obtain robust and reliable causal inference on the impact of educational duration on BMD and risk factors for osteoporosis, we conducted a multiplicative random-effects inverse-variance weighted (MRE-IVW) analysis in the main analysis. Sensitivity analyses were performed using the Weighted median(WM) method and MR-Egger regression. The MR-Egger regression was not constrained by a zero intercept and can identify genotype-outcome dose–response relationships considering pleiotropic effects. However, the MR-Egger method was more sensitive in detecting associations between unobserved genetic variation and confounders in the exposure-outcome association and required a larger sample size for the same level of potential exposure variation. The WM method provided consistent effect estimates when at least 50% of the information in the analysis came from valid instruments. The heterogeneity was assessed using the Cochran’s Q test with the IVW method [18]. Heterogeneity was considered non-existent when the *p*-value of Cochran’s Q was > 0.05. The intercept term obtained from the MR-Egger regression was used to examine horizontal pleiotropy. Leave-one-out analyses were then performed to assess whether the IVW estimates were biased by the influence of single SNPs. The leave-one-out method was used as a sensitivity analysis. We looked up each SNP in Phenoscanner (http://www.phenoscanner.medschl.cam.ac.uk/). All the statistical analyses were performed using R software(version 4.0.2, TwoSampleMR package 0.5.5).

## Results

**Fig. 1:**
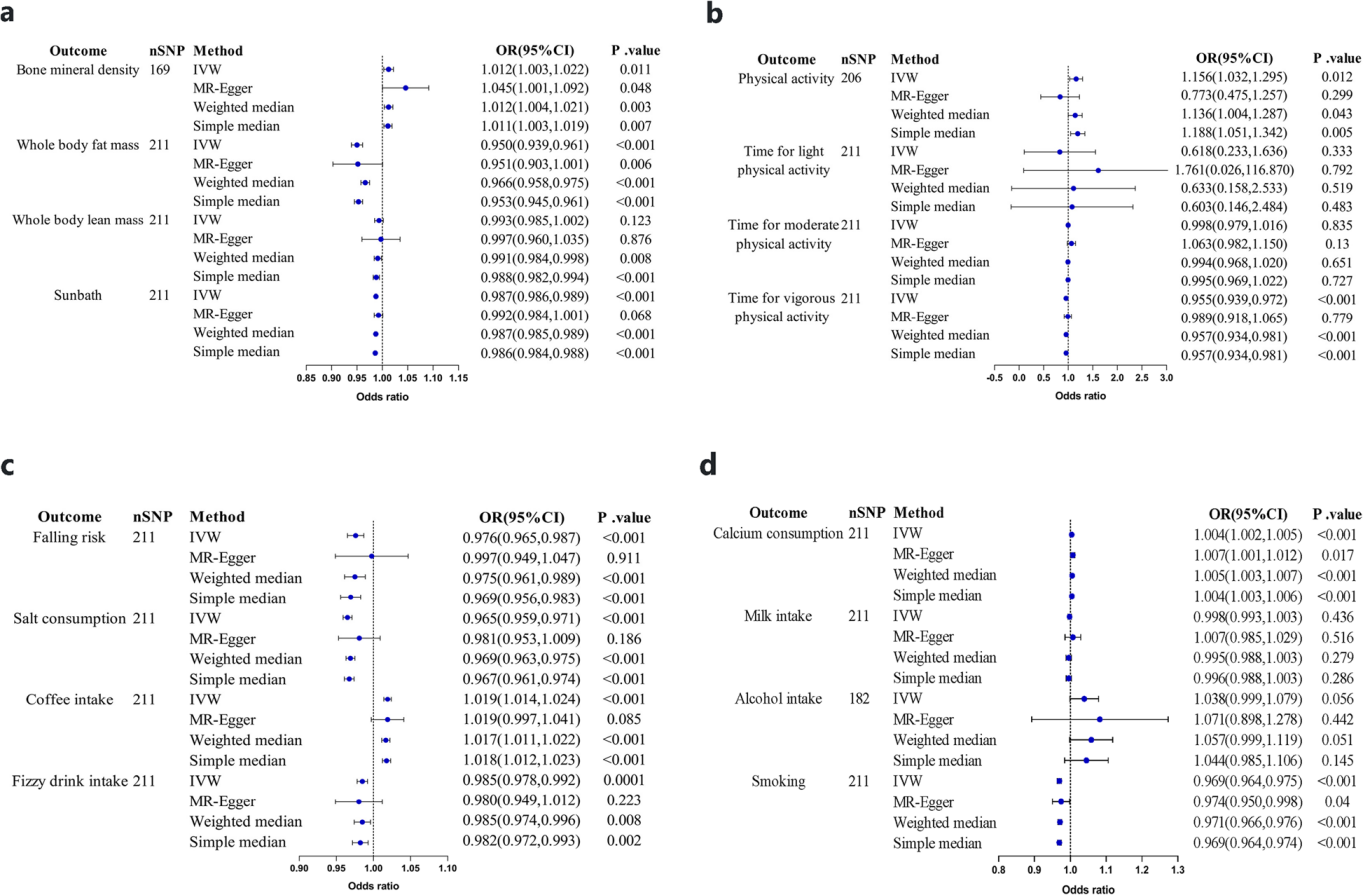
Forest plot of the causal relationships between Years of education and outcomes using different MR methods. **a** Forest plot of the causal influence of Years of education on BMD, Whole body fat mass, Whole body lean mass, Sunbath; **b** Forest plot of the causal relationships between Years of education and PA, Time for light PA, Time for moderate PA,Time for vigorous PA; (**c**) Forest plot of the causal relationships between Years of education and Falling risk, Salt consumption, Coffee intake, Fizzy drink intake; **d** Forest plot of the causal relationships between Years of education and Calcium consumption, Milk intake, Alcohol intake, Smoking. BMD, bone mineral density; PA, physical activity; OR, odds ratio; CI, confidence interval; MR, Mendelian randomization; SNP, single nucleotide polymorphism; IVW, inverse variance weighted

**Fig. 2:**
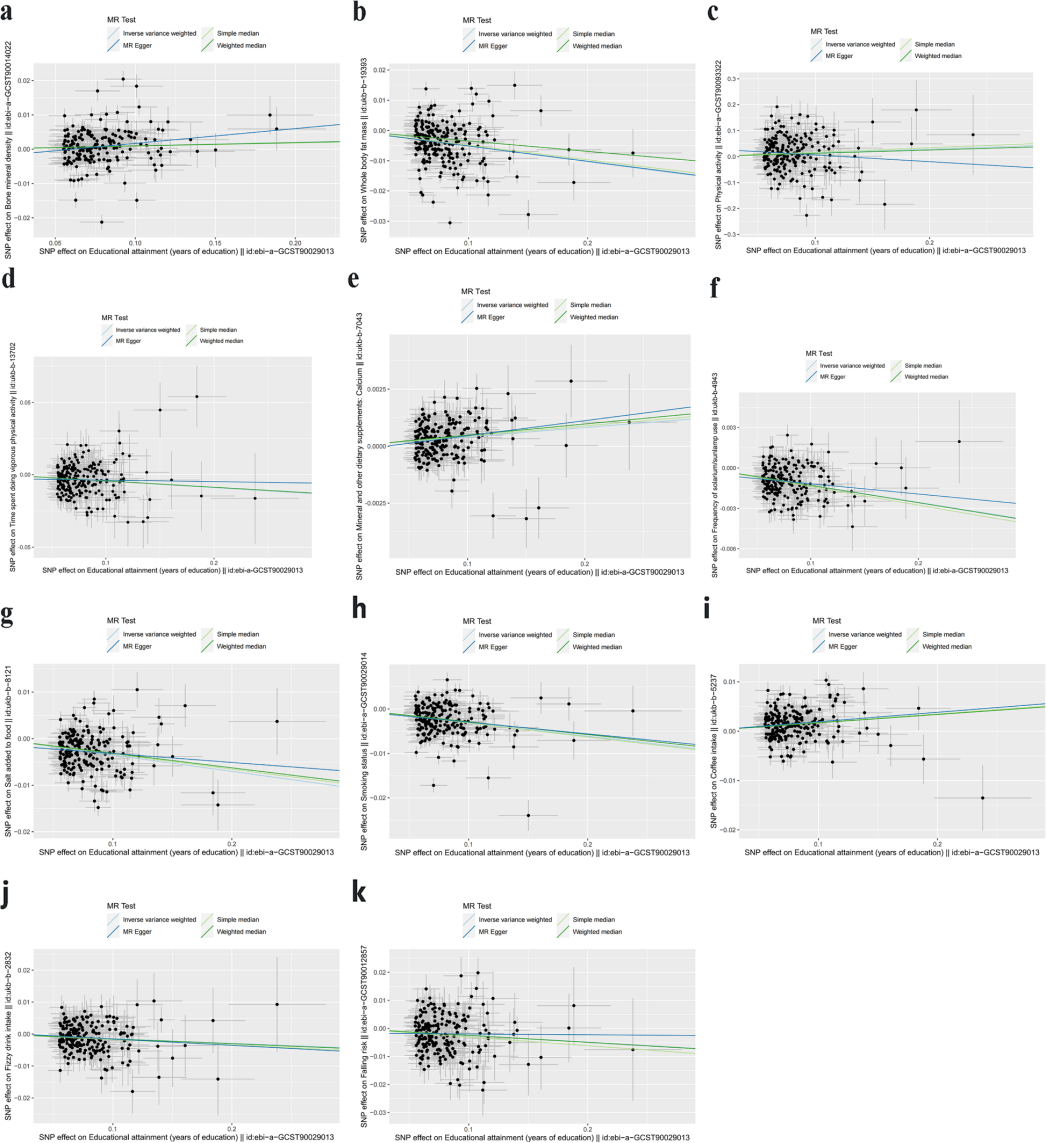
Scatter plot of the causal relationships between Years of education and outcomes using different MR methods. **a** Scatter plot of the causal relationships between Years of education and BMD; **b** Scatter plot of the causal relationships between Years of education and Whole body fat mass; **c** Scatter plot of the causal relationships between Years of education and Physical activity; **d** Scatter plot of the causal relationships between Years of education and Time for vigorous physical activity; **e** Scatter plot of the causal relationships between Years of education and Calcium consumption; **f** Scatter plot of the causal relationships between Years of education and Sunbath; **g** Scatter plot of the causal relationships between Years of education and Salt consumption; **h** Scatter plot of the causal relationships between Years of education and Smoking; **i** Scatter plot of the causal relationships between Years of education and Coffee intake; **j** Scatter plot of the causal relationships between Years of education and Fizzy drink; **k** Scatter plot of the causal relationships between Years of education and Falling risk. The slope of each line corresponds to the causal estimates for each method. The individual SNP effect on the outcome (point and vertical line) against its effect on the exposure (point and horizontal line) was delineated in the background. BMD, bone mineral density; MR, Mendelian randomization; SNP, single nucleotide polymorphism

**Fig. 3:**
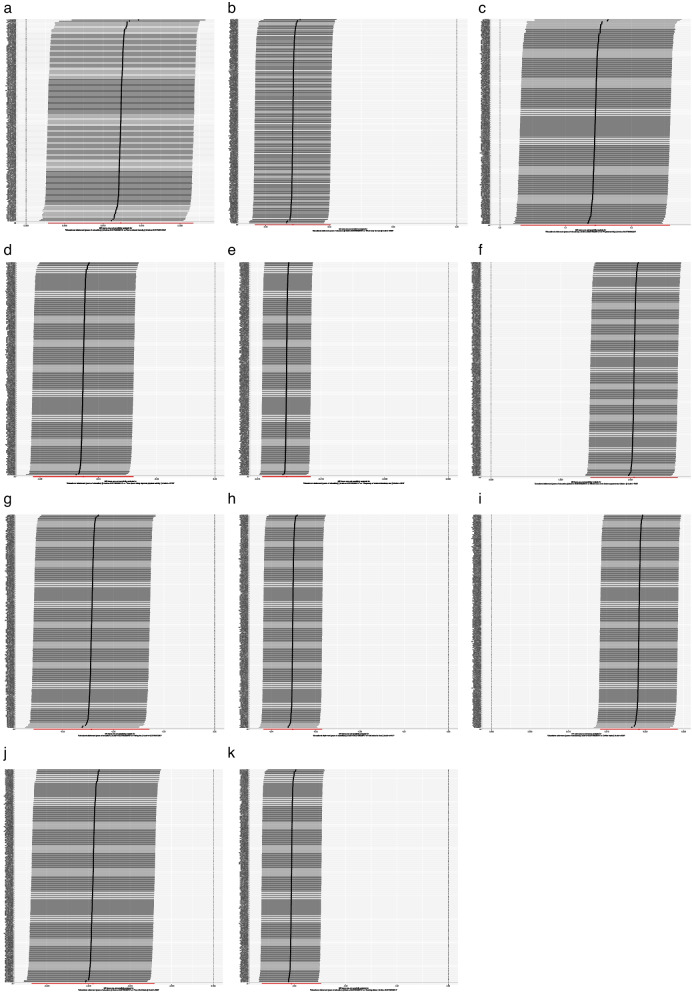
Results of leave-one-out method sensitivity analysis. **a** Leave-one-out sensitivity analysis for the effect of Years of education on BMD; **b** Leave-one-out sensitivity analysis for the effect of Years of education on Whole body fat mass; **c** Leave-one-out sensitivity analysis for the effect of Years of education on PA; **d** Leave-one-out sensitivity analysis for the effect of Years of education on Time for vigorous PA; **e** Leave-one-out sensitivity analysis for the effect of Years of education on Sunbath; **f** Leave-one-out sensitivity analysis for the effect of Years of education on Calcium consumption; **g** Leave-one-out sensitivity analysis for the effect of Years of education on Falling risk; **h** Leave-one-out sensitivity analysis for the effect of Years of education on Salt consumption; **i** Leave-one-out sensitivity analysis for the effect of Years of education on Coffee intake; **j** Leave-one-out sensitivity analysis for the effect of Years of education on Fizzy drink; **k** Leave-one-out sensitivity analysis for the effect of Years of education on smoking. BMD, bone mineral density; PA, physical activity; MR, Mendelian randomization

A total of 169 SNPs were included as IVs in the analysis of the association between educational duration and BMD (Fig. 1a). The MR results showed consistent directions of effect for the IVW, MR-Egger, WM, and simple median methods (Fig. 2a). The result of IVW method suggested a significant association between educational duration and BMD (beta = 0.012, se = 0.005, OR = 1.012, 95%CI:1.003–1.022, *P* = 0.011, F = 183.210) (Tables 2 and 3). Sensitivity analyses indicated that none of the SNPs had a substantial impact on the estimated causal association when individually excluded (Fig. 3a). The *p*-value of Cochran’s Q was 4.607e-61 (Table 3). There was no evidence of horizontal pleiotropy(Mr_pleiotropy *P*.value = 0.142) (Table 3).

**Table 2:** Mendelian randomization estimates of years of education on outcome variable

Outcomes	Method	Estimate-beta	SE	OR(95%CI)	*P*.value	Adjust *P*.value
Bone mineral density	IVW	0.012	0.005	1.012(1.003,1.022)	0.011	0.014
	MR-Egger	0.044	0.022	1.045(1.001,1.092)	0.048	
	Weighted median	0.012	0.004	1.012(1.004,1.021)	0.003	
	Simple median	0.011	0.004	1.011(1.003,1.019)	0.007	
Whole body fat mass	IVW	-0.051	0.006	0.950(0.939,0.961)	< 0.001	< 0.001
	MR-Egger	-0.050	0.026	0.951(0.903,1.001)	0.006	
	Weighted median	-0.034	0.004	0.966(0.958,0.975)	< 0.001	
	Simple median	-0.048	0.004	0.953(0.945,0.961)	< 0.001	
Whole body lean mass	IVW	-0.007	0.004	0.993(0.985,1.002)	0.123	0.157
	MR-Egger	-0.003	0.019	0.997(0.960,1.035)	0.876	
	Weighted median	-0.009	0.003	0.991(0.984,0.998)	0.008	
	Simple median	-0.012	0.003	0.988(0.982,0.994)	< 0.001	
Sunbath	IVW	-0.013	0.001	0.987(0.986,0.989)	< 0.001	< 0.001
	MR-Egger	-0.008	0.004	0.992(0.984,1.001)	0.068	
	Weighted median	-0.013	0.001	0.987(0.985,0.989)	< 0.001	
	Simple median	-0.014	0.001	0.986(0.984,0.988)	< 0.001	
Physical activity	IVW	0.145	0.058	1.156(1.032,1.295)	0.012	0.017
	MR-Egger	-0.258	0.248	0.773(0.475,1.257)	0.299	
	Weighted median	0.128	0.063	1.136(1.004,1.287)	0.043	
	Simple median	0.172	0.062	1.188(1.051,1.342)	0.005	
Time for light physical activity	IVW	-0.480	0.496	0.618(0.233,1.636)	0.333	0.409
	MR-Egger	0.566	2.140	1.761(0.026,116.870)	0.792	
	Weighted median	-0.457	0.707	0.633(0.158,2.533)	0.519	
	Simple median	-0.506	0.723	0.603(0.146,2.484)	0.483	
Time for moderate physical activity	IVW	-0.002	0.009	0.998(0.979,1.016)	0.835	0.971
	MR-Egger	0.061	0.040	1.063(0.982,1.150)	0.130	
	Weighted median	-0.006	0.013	0.994(0.968,1.020)	0.651	
	Simple median	-0.005	0.014	0.995(0.969,1.022)	0.727	
Time for vigorous physical activity	IVW	-0.045	0.009	0.955(0.939,0.972)	< 0.001	< 0.001
	MR-Egger	-0.011	0.038	0.989(0.918,1.065)	0.779	
	Weighted median	-0.043	0.012	0.957(0.934,0.981)	< 0.001	
	Simple median	-0.044	0.012	0.957(0.935,0.980)	< 0.001	
Falling risk	IVW	-0.024	0.006	0.976(0.965,0.987)	< 0.001	< 0.001
	MR-Egger	-0.003	0.025	0.997(0.949,1.047)	0.911	
	Weighted median	-0.025	0.007	0.975(0.961,0.989)	< 0.001	
	Simple median	-0.031	0.007	0.969(0.956,0.983)	< 0.001	
Salt consumption	IVW	-0.035	0.003	0.965(0.959,0.971)	< 0.001	< 0.001
	MR-Egger	-0.019	0.014	0.981(0.953,1.009)	0.186	
	Weighted median	-0.031	0.003	0.969(0.963,0.975)	< 0.001	
	Simple median	-0.033	0.003	0.967(0.961,0.974)	< 0.001	
Smoking	IVW	-0.031	0.003	0.969(0.964,0.975)	< 0.001	< 0.001
	MR-Egger	-0.026	0.013	0.974(0.950,0.998)	0.040	
	Weighted median	-0.029	0.002	0.971(0.966,0.976)	< 0.001	
	Simple median	-0.031	0.003	0.969(0.964,0.974)	< 0.001	
Coffee intake	IVW	0.019	0.003	1.019(1.014,1.024)	< 0.001	< 0.001
	MR-Egger	0.019	0.011	1.019(0.997,1.041)	0.085	
	Weighted median	0.017	0.002	1.017(1.011,1.022)	< 0.001	
	Simple median	0.018	0.003	1.018(1.012,1.023)	< 0.001	
Fizzy drink intake	IVW	-0.014	0.004	0.985(0.978,0.992)	0.0001	0.0002
	MR-Egger	-0.019	0.016	0.980(0.949,1.012)	0.223	
	Weighted median	-0.015	0.006	0.985(0.974,0.996)	0.008	
	Simple median	-0.017	0.005	0.982(0.972,0.993)	0.002	
Calcium consumption	IVW	0.004	0.001	1.004(1.002,1.005)	< 0.001	< 0.001
	MR-Egger	0.007	0.003	1.007(1.001,1.012)	0.017	
	Weighted median	0.005	0.001	1.005(1.003,1.007)	< 0.001	
	Simple median	0.004	0.001	1.004(1.003,1.006)	< 0.001	
Milk intake	IVW	-0.002	0.003	0.998(0.993,1.003)	0.436	0.528
	MR-Egger	0.007	0.011	1.007(0.985,1.029)	0.516	
	Weighted median	-0.004	0.003	0.995(0.988,1.003)	0.279	
	Simple median	-0.004	0.004	0.996(0.988,1.003)	0.286	
Alcohol intake	IVW	0.038	0.019	1.038(0.999,1.079)	0.056	0.075
	MR-Egger	0.069	0.089	1.071(0.898,1.278)	0.442	
	Weighted median	0.056	0.028	1.057(0.999,1.119)	0.051	
	Simple median	0.043	0.029	1.044(0.985,1.106)	0.145	

*beta* allele effect value, *se* standard error, *OR* odds ratio, *CI* confidence interval, *MR* Mendelian randomization, *IVW* inverse variance weighted

**Table 3:** Reliability test of MR analysis results

**Outcomes**	**nSNPs**	**R2**	**F**	Q_*P*.value	**MR-PRESSO *P*.value**	**Mr_pleiotropy *P*.value**	**Egger_intercept**
Bone mineral density	169	0.212	183.210	4.607e-61	0.580	0.142	-0.002
Whole body fat mass	211	0.021	196.813	4.026e-273	0.338	0.969	-7.790e-05
Whole body lean mass	211	0.029	269.872	1.816e-221	0.626	0.836	-0.0003
Sunbath	211	0.0217	204.919	5.140e-07	0.769	0.211	-0.0004
Physical activity	206	0.103	971.005	1.989e-20	0.762	0.097	0.032
Time for light physical activity	211	0.145	1403.242	0.375	0.385	0.616	-0.083
Time for moderate physical activity	211	0.145	1403.242	0.126	0.105	0.108	-0.005
Time for vigorous physical activity	211	0.145	1403.242	0.014	0.014	0.348	-0.003
Falling risk	211	0.021	198.105	1.189e-07	0.577	0.378	-0.002
Salt consumption	211	0.020	193.198	1.304e-57	0.934	0.258	-0.001
Smoking	211	0.020	190.911	2.778e-87	0.203	0.726	-0.0003
Coffee intake	211	0.022	208.422	3.153e-26	0.669	0.996	3.885e-06
Fizzy drink intake	211	0.145	1403.242	0.611	0.640	0.735	0.0004
Calcium consumption	211	0.020	193.720	3.908e-05	0.851	0.351	-0.0002
Milk intake	211	0.145	1403.377	0.596	0.601	0.394	-0.0007
Alcohol intake	182	0.095	756.346	0.341	0.351	0.720	-0.002

*SNPs* single nucleotide polymorphisms, *MR-PRESSO* MR pleiotropy residual sum and outlier, *Q_P.value* the *p*-value of Cochran’s Q

**Fig. 4:**
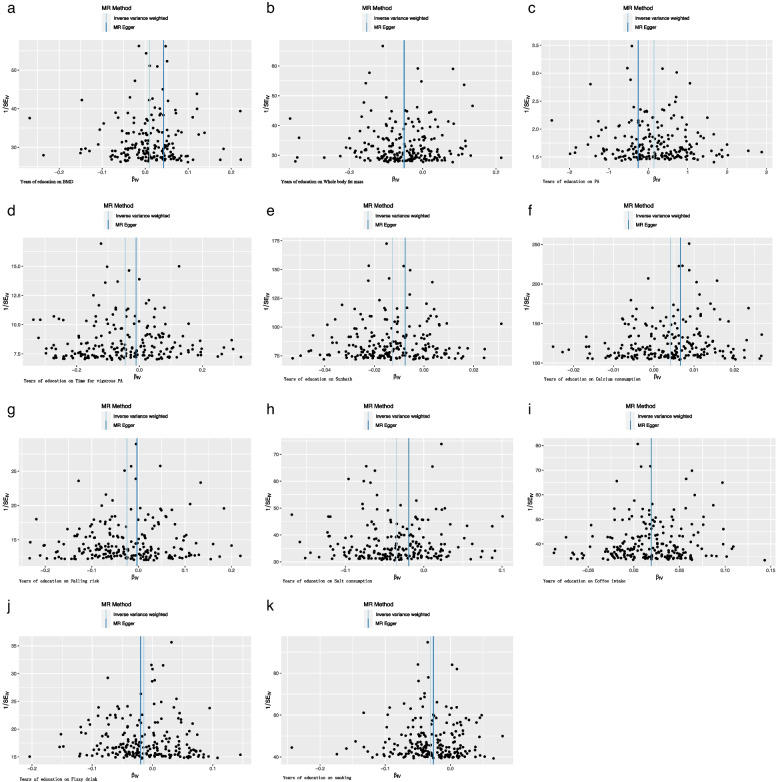
Funnel plots. **a** Funnel plot for the effect of Years of education on BMD; **b** Funnel plot for the effect of Years of education on Whole body fat mass; **c** Funnel plot for the effect of Years of education on PA; **d** Funnel plot for the effect of Years of education on Time for vigorous PA; **e** Funnel plot for the effect of Years of education on Sunbath; **f** Funnel plot for the effect of Years of education on Calcium consumption; **g** Funnel plot for the effect of Years of education on Falling risk; **h** Funnel plot for the effect of Years of education on Salt consumption; **i** Funnel plot for the effect of Years of education on Coffee intake; **j** Funnel plot for the effect of Years of education on Fizzy drink; **k** Funnel plot for the effect of Years of education on smoking. BMD, bone mineral density; PA, physical activity; MR, Mendelian randomization

With the risk factors for osteoporosis, the IVW results indicated that there was a positive causal relationship between educational duration and PA (nSNPs = 206, beta = 0.145, se = 0.058, OR = 1.156, 95%CI:1.032–1.295, *P* = 0.012, F = 971.005, Mr_pleiotropy *P*.value = 0.097), calcium consumption (nSNPs = 211, beta = 0.004, se = 0.001, OR = 1.004, 95%CI:1.002–1.005, *P* = 0.012, F = 193.720, Mr_pleiotropy *P*.value = 0.351), and coffee intake (nSNPs = 211, beta = 0.019, se = 0.003, OR = 1.019, 95%CI:1.014–1.024, *P* = 0.012, F = 208.421, Mr_pleiotropy *P*.value = 0.996). There was a negative association between whole body fat mass (nSNPs = 211, beta = -0.051, se = 0.006, OR = 0.949, 95%CI:0.938–0.961, *P* < 0.001, F = 196.813, Mr_pleiotropy *P*.value = 0.969), time for vigorous PA (nSNPs = 211, beta = -0.045, se = 0.009, OR = 0.955, 95%CI:0.939–0.972, *P* < 0.001, F = 1403.241, Mr_pleiotropy *P*.value = 0.348), sunbath (nSNPs = 211, beta = -0.013, se = 0.001, OR = 0.987, 95%CI:0.986–0.989, *P* < 0.001, F = 204.919, Mr_pleiotropy *P*.value = 0.211), salt consumption (nSNPs = 211, beta = -0.035, se = 0.003, OR = 0.965, 95%CI:0.959–0.971, *P* < 0.001, F = 193.198, Mr_pleiotropy *P*.value = 0.258), fizzy drink intake (nSNPs = 211, beta = -0.014, se = 0.004, OR = 0.985, 95%CI:0.978–0.992, *P* = 0.0001, F = 1403.242, Mr_pleiotropy *P*.value = 0.735), smoking (nSNPs = 211, beta = -0.031, se = 0.003, OR = 0.969, 95%CI:0.964–0.975, *P* < 0.001, F = 190.910, Mr_pleiotropy *P*.value = 0.726), and falling risk (nSNPs = 211, beta = -0.024, se = 0.006, OR = 0.976, 95%CI:0.965–0.987, *P* < 0.001, F = 198.104, Mr_pleiotropy *P*.value = 0.378) (Tables 2 and 3). There was no significant association between educational duration and lean mass, time for light-to-moderate PA, the intake of milk and alcohol (Fig. 1). Horizontal pleiotropy was absent in this study (Table 3). The results were robust under sensitivity analyses (Figs. 2, 3 and 4).

## Discussion

Osteoporosis has emerged as a major public health issue. Preventing osteoporosis presents significant challenges, including high incidence rates and low awareness, diagnosis, and treatment rates [1, 2]. Osteoporosis diagnosis and treatment vary significantly across regions and between urban and rural areas [4]. Identifying the underlying causes is crucial for devising effective measures to promote treatment in all regions. When assessing the impact of educational attainment on osteoporosis, it is essential to examine whether higher education levels promote awareness of the condition and enhance prevention and treatment efforts.

Based on a study of 1,424 Mexican–American women aged 67 or older, there was a significant correlation between higher education levels and lower incidence of osteoporosis (OR = 1.13, 95%CI:1.05–1.20) [19]. Okbay et al. [20] found that the polygenic index of educational attainment (EA PGI) significantly predicted osteoporosis (Incremental Nagelkerke’s R^2^ = 0.030%, 95% CI: 0.017% ~ 0.050%, *P*-value = 2.985E-08). It should be noted that a substantial portion of the predictive power of EA PGI arises from factors other than direct effects, in addition to the direct effects. A study found that individuals with lower educational attainment had a higher prevalence of unhealthy lifestyle factors related to osteoporosis, such as low milk consumption and lack of exercise (*P* < 0.05) [21]. A survey conducted in a community of 560 women aged 40 or older revealed that illiterate women were less likely to take calcium supplements, exercise, or engage in daily activity for less than 20 min, compared to those with higher educational levels [22]. They faced more barriers to implementing healthy practices and had less motivation for health [23]. A multicenter longitudinal study in Italy found a negative correlation between lower education level (45.8%) and body mass index(BMI) (*P* = 0.013), smoking (*P* < 0.001), and fractures (*P* < 0.001) [18].

Previous studies have identified correlations between different educational levels and partial risk factors for osteoporosis. However, these studies were based on cross-sectional data or small sample sizes. It has not been established whether there is a causal relationship between them. With the establishment of public databases, there is now an opportunity to use MR to analyze the causal relationship between educational duration and the risk factors for osteoporosis, based on large sample sizes. MR utilizes genetic variation as an IV to overcome confounding factors and reverse causality effects. It has been applied in various studies to analyze the relationship between exposure and outcome, providing a more accurate analysis and understanding of their association [24]. Our MR analysis clarified the nature and extent of the relationship between educational duration and risk factors associated with osteoporosis, providing a better understanding of the relationship between educational attainment and osteoporosis risk. The results showed a positive causal relationship between years of education and BMD, PA, calcium consumption, and coffee intake. There was a negative causal roles of educational duration in whole-body fat mass, sunbath, time for vigorous PA, salt consumption, fizzy drink intake, smoking, and falling risk. In previous studies, Zhou J et al. also observed a significant dose–response positive correlation between educational level and BMD (*P* = 0.011) among 685 postmenopausal women aged 48–63 after adjusting for age and weight [25]. According to the data from the National Youth Longitudinal Study involving 12,686 participants, higher educational attainment mediated a positive relationship between self-reported PA and individual control and health [26]. A cross-sectional study analyzed data from 3,924 healthy men and women aged 65–95 and found that certain factors such as calcium supplementation, PA, educational level, and maintaining a normal BMI were positively associated with BMD [27]. Fravel et al. found a significant association between higher education and increased calcium supplement consumption in a study of 15,729 participants [28]. Low educational attainment was linked to increased odds of obesity, lack of physical activity, and smoking among 13,714 women aged 45–50 [29]. Compared to low-income and lower-educated individuals, higher-income and better-educated individuals had more knowledge of diseases related to high sodium intake [30]. Among 2,989 participants, Clermont et al. discovered that individuals with lower educational levels (87.1%) tended to add more salt to meals consumed at home [31]. In another study, lower educational attainment was found to be a statistically significant predictor of longer sunlight exposure(β = -0.18, *p* < 0.001) [32]. In a questionnaire survey conducted by researchers, participants were interviewed regarding environmental, dietary, and genetic risk factors. The results showed that higher educational attainment was associated with reduced consumption of fruit juice and fizzy drink [33]. De Roza et al. discovered statistical differences in fall risk among 360 older adults based on their educational attainment [34], which was consistent with our study findings.

However, there were some differences noted. A study of 19 cohorts found that high school graduation was associated with decreased moderate-to-vigorous physical activity of -7.04 min/day (95% CI:-11.26, -2.82). The study also suggested that the transition from high school was a crucial moment to prevent decreases in physical activity and increases in weight [35]. A dietary intake survey revealed that people with higher educational levels had higher intake of alcohol and coffee compared to their counterparts with lower educational levels [36] Low educational level was associated with decreased lean mass and high fat mass in a study by Mantovani et al. [37]. Our results showed no causal effect of educational duration on lean mass, time for light-to-moderate PA, as well as the intake of milk and alcohol. The results suggested a lack of correlation between educational duration and the aforementioned risk factors, which did not support the hypothesis of a causal relationship. This was due to the assumption of IVs not being strongly correlated with the exposure in MR.

In light of these findings, this study endeavors to delve into the potential mechanisms that underlie the observed correlation between educational duration and the risk factors associated with osteoporosis. Education now emphasizes holistic development, exposing individuals to a greater variety of health promotion and knowledge dissemination activities. Those with higher educational attainment benefit more from these efforts. Increased exposure and cognitive abilities lead to greater emphasis on health management and heightened health awareness. Individuals with longer educational years are more aware of the harms of smoking, risks associated with high-salt diet, and the consumption of carbonated beverages. This heightened awareness leads to greater constraint and influence on their behavior. They develop a deeper understanding of the detrimental effects of these behaviors and gradually adopt healthier habits, such as quitting smoking and limiting the intake of salt and fizzy drink. Additionally, the educational environment incorporates physical education, and intervention programs focusing on physical activity have been effective in increasing exercise levels [38]. Therefore, targeting behavior change interventions among individuals with higher educational attainment, whether at the individual, family, societal, or school level, can have upstream benefits [39]. Consequently, individuals with longer educational years prioritize physical exercise, pay attention to calcium intake, which promotes bone health, increases BMD, and reduces the falling risk by enhancing muscle balance and strength. Meanwhile, individuals with higher levels of education place greater emphasis on body management and tend to focus on weight reduction, resulting in a decrease in overall body fat without a reduction in lean mass [40]. Additionally, individuals with longer educational years are more likely to be engaged in mental labor, requiring sustained energy and focus. As a result, their coffee consumption tends to be relatively higher [41]. However, their outdoor activity time is noticeably reduced, leading to decreased exposure to sunlight. Moreover, due to time constraints and the nature of their work, they may find it challenging to engage in prolonged and intense physical exercise. Instead, they often opt for moderate aerobic and resistance exercises during suitable periods [42]. Given the demands of modern work and social life, alcohol consumption is a common way of socializing, and thus, is not significantly influenced by educational attainment. Furthermore, with improvements in the economy and material living standards, the consumption of dairy products, such as milk, has become widespread [43] and does not exhibit notable differences across educational levels.

We should also consider some limitations. Firstly, all included participants are of European origin, and it remains unclear whether our findings are applicable to other populations. Secondly, there may be some overlap of samples between educational duration and outcomes, which could potentially impact the results. Thirdly, MR findings primarily capture the long-term effects of a genetically predisposed status of education. They might not accurately reflect the short-term impact of educational interventions or policies.

## Conclusion

Longer educational duration is causally linked with increased BMD, physical activity, calcium intake, and coffee consumption, but negatively associated with whole body fat mass, sunbath, time for intense physical activity, salt and fizzy drink intake, smoking, and falling risk. No causal relationship was found between educational duration and lean mass, time for light-to-moderate physical activity, milk intake, and alcohol consumption. These findings have important implications for public health policies and call for further research in this field.

### Supplementary Information

**Supplementary Material 1.**

## Data Availability

Data supporting the findings of this study were available within the paper. The data used in this study were obtained from the IEU GWAS database at the University of Bristol (https://gwasmrcieu.ac.uk).
