# Supplementary material for: Causal roles of educational duration in bone mineral density and risk factors for osteoporosis: a Mendelian randomization study
Source: BMC Musculoskelet Disord. 2024 May 2;25:345. doi: 10.1186/s12891-024-07428-8 (PMC11064366; doi:10.1186/s12891-024-07428-8)
Supplement: Supplementary file 1 — Supplementary Material 1. [file 12891_2024_7428_MOESM1_ESM.zip › IVs of Educational attainment on BMD.docx]

| SNP | b | se | P.value | adjust P.value |
| --- | --- | --- | --- | --- |
| rs10058365 | 0.012114046 | 0.004842362 | 0.012360526 | 0.014730105 |
| rs10066409 | 0.012426769 | 0.004834185 | 0.010152175 | 0.014730105 |
| rs1010334 | 0.012389567 | 0.00483329 | 0.010365809 | 0.014730105 |
| rs10189857 | 0.011777652 | 0.004826143 | 0.014671582 | 0.015604517 |
| rs10215082 | 0.012539737 | 0.004827235 | 0.009384885 | 0.014730105 |
| rs10511592 | 0.012245593 | 0.004836259 | 0.011340159 | 0.014730105 |
| rs10518019 | 0.01224317 | 0.004843266 | 0.011475535 | 0.014730105 |
| rs10745789 | 0.012416567 | 0.004832547 | 0.010188565 | 0.014730105 |
| rs10765775 | 0.012040003 | 0.004840018 | 0.012861016 | 0.014730105 |
| rs10844179 | 0.012072254 | 0.004829268 | 0.012425975 | 0.014730105 |
| rs10854884 | 0.011484331 | 0.004794256 | 0.016600632 | 0.016798259 |
| rs11138947 | 0.012558108 | 0.0048259 | 0.009261932 | 0.014730105 |
| rs11155821 | 0.011467908 | 0.004774921 | 0.01631927 | 0.016712505 |
| rs11243838 | 0.012059165 | 0.004827654 | 0.012491906 | 0.014730105 |
| rs11249939 | 0.014593949 | 0.004423424 | 0.000969461 | 0.014730105 |
| rs11572842 | 0.012573223 | 0.004821901 | 0.009119969 | 0.014730105 |
| rs115877304 | 0.012378238 | 0.004835192 | 0.0104663 | 0.014730105 |
| rs11635966 | 0.012485005 | 0.004833184 | 0.009789307 | 0.014730105 |
| rs11661305 | 0.012077392 | 0.004833078 | 0.012457857 | 0.014730105 |
| rs11678980 | 0.01232765 | 0.004849164 | 0.011015037 | 0.014730105 |
| rs11693764 | 0.012063734 | 0.00482702 | 0.012447078 | 0.014730105 |
| rs11714679 | 0.01255233 | 0.004825831 | 0.009293318 | 0.014730105 |
| rs11720121 | 0.013121177 | 0.004786324 | 0.006117995 | 0.014730105 |
| rs11732657 | 0.012281747 | 0.00483452 | 0.011071715 | 0.014730105 |
| rs11736863 | 0.012438661 | 0.004838995 | 0.01015503 | 0.014730105 |
| rs11764590 | 0.012197405 | 0.004837129 | 0.011681549 | 0.014730105 |
| rs11871429 | 0.012099093 | 0.004831686 | 0.012275846 | 0.014730105 |
| rs12029988 | 0.012116887 | 0.004832972 | 0.012171618 | 0.014730105 |
| rs12132451 | 0.012365619 | 0.004839653 | 0.010616859 | 0.014730105 |
| rs12468040 | 0.012758053 | 0.004830256 | 0.008259365 | 0.014730105 |
| rs12474895 | 0.01236911 | 0.004834869 | 0.010518126 | 0.014730105 |
| rs12503522 | 0.01224103 | 0.004833728 | 0.01132782 | 0.014730105 |
| rs12532494 | 0.012371271 | 0.004843288 | 0.010639769 | 0.014730105 |
| rs12574281 | 0.012183488 | 0.004832816 | 0.011702463 | 0.014730105 |
| rs12663818 | 0.012508582 | 0.004828149 | 0.009576385 | 0.014730105 |
| rs12735232 | 0.012558832 | 0.004828179 | 0.0092911 | 0.014730105 |
| rs12921005 | 0.012236109 | 0.004833806 | 0.011362077 | 0.014730105 |
| rs12967855 | 0.011679026 | 0.004847748 | 0.015989053 | 0.01647357 |
| rs1334297 | 0.012765734 | 0.004844943 | 0.008417256 | 0.014730105 |
| rs1363862 | 0.012237338 | 0.004833653 | 0.01135125 | 0.014730105 |
| rs1369128 | 0.012118682 | 0.004833647 | 0.012170881 | 0.014730105 |
| rs1381247 | 0.0120339 | 0.00482383 | 0.012607172 | 0.014730105 |
| rs1391438 | 0.011292476 | 0.004778299 | 0.018113797 | 0.01822098 |
| rs1452075 | 0.01230301 | 0.004834901 | 0.01093951 | 0.014730105 |
| rs145590108 | 0.012396916 | 0.004836373 | 0.010369223 | 0.014730105 |
| rs1566085 | 0.012807021 | 0.004834874 | 0.008075796 | 0.014730105 |
| rs1620977 | 0.01108943 | 0.004769154 | 0.020059105 | 0.020059105 |
| rs17489649 | 0.012610061 | 0.004822292 | 0.008924005 | 0.014730105 |
| rs17513684 | 0.012535385 | 0.004827443 | 0.009412612 | 0.014730105 |
| rs17628095 | 0.012325563 | 0.004836336 | 0.010817696 | 0.014730105 |
| rs1788783 | 0.011858254 | 0.004823002 | 0.013944603 | 0.015099251 |
| rs1812587 | 0.012354668 | 0.004834954 | 0.010610198 | 0.014730105 |
| rs1835340 | 0.012171808 | 0.004832154 | 0.011771555 | 0.014730105 |
| rs185291 | 0.012055849 | 0.00485833 | 0.013083799 | 0.014730105 |
| rs1869165 | 0.012106896 | 0.004829785 | 0.012185875 | 0.014730105 |
| rs1880692 | 0.012312021 | 0.004834301 | 0.010871418 | 0.014730105 |
| rs1892417 | 0.011577253 | 0.004789277 | 0.015634927 | 0.016306365 |
| rs1917008 | 0.012005673 | 0.004821167 | 0.012767096 | 0.014730105 |
| rs1964927 | 0.012084804 | 0.004831891 | 0.012382557 | 0.014730105 |
| rs2145265 | 0.012342552 | 0.004834491 | 0.01067933 | 0.014730105 |
| rs215632 | 0.012630147 | 0.004818838 | 0.008767369 | 0.014730105 |
| rs2175420 | 0.012558362 | 0.004827755 | 0.009287559 | 0.014730105 |
| rs2182398 | 0.012539302 | 0.004825228 | 0.009357844 | 0.014730105 |
| rs2190872 | 0.01205357 | 0.004826218 | 0.012506586 | 0.014730105 |
| rs2287838 | 0.012056791 | 0.004826342 | 0.01248532 | 0.014730105 |
| rs2309812 | 0.01195834 | 0.004846637 | 0.013611807 | 0.014833379 |
| rs2332818 | 0.012097008 | 0.004828481 | 0.012233219 | 0.014730105 |
| rs2411453 | 0.012201366 | 0.004846366 | 0.011814671 | 0.014730105 |
| rs2559509 | 0.013104993 | 0.004754769 | 0.005848107 | 0.014730105 |
| rs2570497 | 0.012400214 | 0.004836671 | 0.01035358 | 0.014730105 |
| rs2604541 | 0.011625563 | 0.004766021 | 0.014717375 | 0.015604517 |
| rs2706762 | 0.012729788 | 0.004812067 | 0.00815971 | 0.014730105 |
| rs2725371 | 0.012165321 | 0.00483883 | 0.011933531 | 0.014730105 |
| rs2735421 | 0.011878253 | 0.00483833 | 0.014087221 | 0.015157136 |
| rs281324 | 0.011912769 | 0.004814327 | 0.013344484 | 0.014813297 |
| rs2820313 | 0.012258203 | 0.004834714 | 0.011230107 | 0.014730105 |
| rs2834011 | 0.012425762 | 0.004833571 | 0.010148713 | 0.014730105 |
| rs2974312 | 0.012658219 | 0.004827587 | 0.008740032 | 0.014730105 |
| rs2998309 | 0.012526429 | 0.004825905 | 0.009440832 | 0.014730105 |
| rs324801 | 0.012657781 | 0.004816404 | 0.008587432 | 0.014730105 |
| rs333078 | 0.012310232 | 0.004834676 | 0.010889118 | 0.014730105 |
| rs34042385 | 0.012007582 | 0.004822372 | 0.01277524 | 0.014730105 |
| rs34192341 | 0.012157416 | 0.004832997 | 0.011886276 | 0.014730105 |
| rs34364916 | 0.012293771 | 0.004834727 | 0.010996642 | 0.014730105 |
| rs34470581 | 0.012325865 | 0.004838244 | 0.010846971 | 0.014730105 |
| rs35039375 | 0.012672579 | 0.004822422 | 0.008592754 | 0.014730105 |
| rs35091253 | 0.012592628 | 0.004835572 | 0.009209834 | 0.014730105 |
| rs35811586 | 0.012069924 | 0.004826857 | 0.01239914 | 0.014730105 |
| rs35917528 | 0.012072273 | 0.004828281 | 0.012407929 | 0.014730105 |
| rs3788556 | 0.012361276 | 0.004837884 | 0.010615735 | 0.014730105 |
| rs3794620 | 0.012480367 | 0.004833821 | 0.009826246 | 0.014730105 |
| rs3800925 | 0.012030151 | 0.004834461 | 0.012831349 | 0.014730105 |
| rs3825083 | 0.012060029 | 0.004831231 | 0.012550902 | 0.014730105 |
| rs3827531 | 0.012672243 | 0.004813218 | 0.008468413 | 0.014730105 |
| rs3847225 | 0.01224746 | 0.00484955 | 0.011553922 | 0.014730105 |
| rs4130477 | 0.012527306 | 0.004825817 | 0.009434533 | 0.014730105 |
| rs4146675 | 0.012176757 | 0.004832237 | 0.011738788 | 0.014730105 |
| rs4246167 | 0.012557765 | 0.004833948 | 0.009381545 | 0.014730105 |
| rs4726070 | 0.012334901 | 0.004837493 | 0.010776806 | 0.014730105 |
| rs4731992 | 0.012092983 | 0.004842214 | 0.012510493 | 0.014730105 |
| rs4780563 | 0.012436468 | 0.004832631 | 0.010069411 | 0.014730105 |
| rs4958568 | 0.012987562 | 0.00477463 | 0.006525837 | 0.014730105 |
| rs55800473 | 0.012959625 | 0.004787048 | 0.006784792 | 0.014730105 |
| rs55842281 | 0.012532723 | 0.004829911 | 0.009464194 | 0.014730105 |
| rs55872852 | 0.012259488 | 0.004834593 | 0.011219558 | 0.014730105 |
| rs56118554 | 0.012611186 | 0.004836551 | 0.009121278 | 0.014730105 |
| rs575113 | 0.011887601 | 0.004809021 | 0.013438282 | 0.014813297 |
| rs59123361 | 0.012410105 | 0.004838817 | 0.010326564 | 0.014730105 |
| rs6071573 | 0.011920657 | 0.004825915 | 0.013506241 | 0.014813297 |
| rs613872 | 0.013112475 | 0.004780625 | 0.006091087 | 0.014730105 |
| rs61787087 | 0.011953079 | 0.004815171 | 0.01305084 | 0.014730105 |
| rs61868084 | 0.012588711 | 0.004824451 | 0.009071284 | 0.014730105 |
| rs62018215 | 0.01142679 | 0.004719308 | 0.015465745 | 0.016229485 |
| rs62182125 | 0.012342993 | 0.00483374 | 0.010664377 | 0.014730105 |
| rs62184483 | 0.012093603 | 0.0048439 | 0.012536672 | 0.014730105 |
| rs62253608 | 0.013065051 | 0.004763742 | 0.006095442 | 0.014730105 |
| rs62389638 | 0.012345977 | 0.004839445 | 0.010737905 | 0.014730105 |
| rs6429911 | 0.012230426 | 0.004836513 | 0.011446326 | 0.014730105 |
| rs6556982 | 0.012384948 | 0.004833209 | 0.010393086 | 0.014730105 |
| rs660001 | 0.0122947 | 0.004840095 | 0.011079622 | 0.014730105 |
| rs66844142 | 0.012045483 | 0.004824683 | 0.012537707 | 0.014730105 |
| rs6760772 | 0.012276673 | 0.004834583 | 0.011106034 | 0.014730105 |
| rs6779254 | 0.012355387 | 0.004839381 | 0.010677121 | 0.014730105 |
| rs6789699 | 0.012511228 | 0.004830994 | 0.009603626 | 0.014730105 |
| rs6935954 | 0.012323831 | 0.00486093 | 0.011235671 | 0.014730105 |
| rs6959579 | 0.012151994 | 0.004831323 | 0.011894738 | 0.014730105 |
| rs702606 | 0.012226372 | 0.004834346 | 0.011436701 | 0.014730105 |
| rs7031698 | 0.011977816 | 0.004820849 | 0.0129702 | 0.014730105 |
| rs7070693 | 0.012391371 | 0.00484181 | 0.010490021 | 0.014730105 |
| rs711793 | 0.012093922 | 0.004829202 | 0.012268306 | 0.014730105 |
| rs71646142 | 0.012310931 | 0.004835285 | 0.010894615 | 0.014730105 |
| rs7195278 | 0.012744831 | 0.004822188 | 0.008218565 | 0.014730105 |
| rs7233920 | 0.012462081 | 0.004837283 | 0.009987654 | 0.014730105 |
| rs72674898 | 0.012151218 | 0.004832141 | 0.011914539 | 0.014730105 |
| rs72828517 | 0.012275786 | 0.004844863 | 0.011284156 | 0.014730105 |
| rs72977992 | 0.012512653 | 0.00482702 | 0.009536141 | 0.014730105 |
| rs73499064 | 0.012329069 | 0.004836503 | 0.010797954 | 0.014730105 |
| rs7526112 | 0.012252674 | 0.004841198 | 0.011376452 | 0.014730105 |
| rs7531271 | 0.011767229 | 0.004827057 | 0.014778396 | 0.015604517 |
| rs75433564 | 0.012342586 | 0.004835802 | 0.010700364 | 0.014730105 |
| rs7583473 | 0.0125642 | 0.004827789 | 0.009255358 | 0.014730105 |
| rs7598246 | 0.012243438 | 0.004836935 | 0.011366035 | 0.014730105 |
| rs7629643 | 0.012377018 | 0.004833428 | 0.010445778 | 0.014730105 |
| rs76608582 | 0.012366956 | 0.004836088 | 0.010551149 | 0.014730105 |
| rs7675394 | 0.012350278 | 0.004843098 | 0.010769877 | 0.014730105 |
| rs77025239 | 0.012335113 | 0.004835432 | 0.010741905 | 0.014730105 |
| rs7758776 | 0.012259236 | 0.004835499 | 0.011236463 | 0.014730105 |
| rs77675579 | 0.011436499 | 0.004744652 | 0.015935133 | 0.01647357 |
| rs7768116 | 0.012177927 | 0.004832733 | 0.011739349 | 0.014730105 |
| rs781289 | 0.012034654 | 0.004832567 | 0.0127627 | 0.014730105 |
| rs7868164 | 0.012317271 | 0.004833933 | 0.010831605 | 0.014730105 |
| rs7868984 | 0.011645506 | 0.004854329 | 0.016440195 | 0.016735528 |
| rs7873964 | 0.012481955 | 0.004832639 | 0.009798948 | 0.014730105 |
| rs7966054 | 0.012694991 | 0.004815747 | 0.008385472 | 0.014730105 |
| rs7977614 | 0.012145253 | 0.004833708 | 0.011983955 | 0.014730105 |
| rs7988201 | 0.01201272 | 0.004828596 | 0.012852572 | 0.014730105 |
| rs7988627 | 0.012164771 | 0.004833821 | 0.011849493 | 0.014730105 |
| rs79937071 | 0.012193582 | 0.004833207 | 0.01163995 | 0.014730105 |
| rs8008382 | 0.013418538 | 0.004658319 | 0.003969772 | 0.014730105 |
| rs8020034 | 0.012114609 | 0.004837175 | 0.01226315 | 0.014730105 |
| rs8057808 | 0.012523007 | 0.004831484 | 0.009543118 | 0.014730105 |
| rs807478 | 0.012694021 | 0.004813342 | 0.008357945 | 0.014730105 |
| rs837065 | 0.012629558 | 0.004829481 | 0.008920233 | 0.014730105 |
| rs868698 | 0.012093012 | 0.004832357 | 0.012331692 | 0.014730105 |
| rs879394 | 0.011932645 | 0.004816254 | 0.013227752 | 0.014794196 |
| rs9372625 | 0.01247088 | 0.004864715 | 0.010361151 | 0.014730105 |
| rs9643120 | 0.0126238 | 0.004826622 | 0.008910928 | 0.014730105 |
| rs9797233 | 0.012157285 | 0.004831452 | 0.011860101 | 0.014730105 |
| rs9888796 | 0.012021607 | 0.004827531 | 0.012766452 | 0.014730105 |
| All | 0.01229661 | 0.004809931 | 0.010572977 | 0.014730105 |
