# Supplementary material for: Causal roles of educational duration in bone mineral density and risk factors for osteoporosis: a Mendelian randomization study
Source: BMC Musculoskelet Disord. 2024 May 2;25:345. doi: 10.1186/s12891-024-07428-8 (PMC11064366; doi:10.1186/s12891-024-07428-8)
Supplement: Supplementary file 1 — Supplementary Material 1. [file 12891_2024_7428_MOESM1_ESM.zip › IVs of Educational attainment on calcium.docx]

| SNP | b | se | P.value | adjust P.value |
| --- | --- | --- | --- | --- |
| rs10058365 | 0.004080471 | 0.00064101 | 1.94E-10 | 2.36E-10 |
| rs10066409 | 0.004117039 | 0.00064038 | 1.28E-10 | 2.36E-10 |
| rs1010334 | 0.004099634 | 0.000640285 | 1.53E-10 | 2.36E-10 |
| rs10189857 | 0.004084073 | 0.000641192 | 1.90E-10 | 2.36E-10 |
| rs10215082 | 0.0041562 | 0.0006378 | 7.20E-11 | 2.36E-10 |
| rs1050847 | 0.004064953 | 0.000638713 | 1.96E-10 | 2.36E-10 |
| rs10511592 | 0.004137575 | 0.000639589 | 9.86E-11 | 2.36E-10 |
| rs10518019 | 0.004116258 | 0.000641169 | 1.36E-10 | 2.36E-10 |
| rs10745789 | 0.004107675 | 0.000640323 | 1.41E-10 | 2.36E-10 |
| rs10760023 | 0.004171815 | 0.00063519 | 5.11E-11 | 2.36E-10 |
| rs10765775 | 0.004009595 | 0.000636137 | 2.92E-10 | 3.03E-10 |
| rs10844179 | 0.004129478 | 0.000639884 | 1.09E-10 | 2.36E-10 |
| rs10854884 | 0.004145644 | 0.000640379 | 9.56E-11 | 2.36E-10 |
| rs10994777 | 0.004114523 | 0.00064086 | 1.36E-10 | 2.36E-10 |
| rs11138947 | 0.004128409 | 0.000639845 | 1.10E-10 | 2.36E-10 |
| rs11155821 | 0.004095285 | 0.000640838 | 1.65E-10 | 2.36E-10 |
| rs11214468 | 0.004130404 | 0.000639964 | 1.09E-10 | 2.36E-10 |
| rs11243838 | 0.004122962 | 0.000640093 | 1.19E-10 | 2.36E-10 |
| rs11249939 | 0.00410481 | 0.000641271 | 1.54E-10 | 2.36E-10 |
| rs11572842 | 0.00412235 | 0.000639942 | 1.18E-10 | 2.36E-10 |
| rs115877304 | 0.004112348 | 0.000640417 | 1.35E-10 | 2.36E-10 |
| rs11604034 | 0.004016013 | 0.000634349 | 2.44E-10 | 2.62E-10 |
| rs11635966 | 0.004190145 | 0.000634163 | 3.91E-11 | 2.36E-10 |
| rs11661305 | 0.004178791 | 0.000636223 | 5.10E-11 | 2.36E-10 |
| rs11678980 | 0.004174533 | 0.000639669 | 6.75E-11 | 2.36E-10 |
| rs11690224 | 0.00414643 | 0.000638541 | 8.38E-11 | 2.36E-10 |
| rs11693764 | 0.004092582 | 0.000640158 | 1.63E-10 | 2.36E-10 |
| rs11714679 | 0.004084886 | 0.000639881 | 1.73E-10 | 2.36E-10 |
| rs11720121 | 0.004067266 | 0.000640181 | 2.11E-10 | 2.37E-10 |
| rs11732657 | 0.004083418 | 0.00063974 | 1.74E-10 | 2.36E-10 |
| rs11736863 | 0.004131143 | 0.000640518 | 1.12E-10 | 2.36E-10 |
| rs11764590 | 0.004057529 | 0.000638828 | 2.13E-10 | 2.37E-10 |
| rs117799466 | 0.004100878 | 0.000640382 | 1.52E-10 | 2.36E-10 |
| rs118083122 | 0.004104705 | 0.000640413 | 1.46E-10 | 2.36E-10 |
| rs11871429 | 0.004135155 | 0.000639697 | 1.02E-10 | 2.36E-10 |
| rs11915747 | 0.004049388 | 0.000640043 | 2.50E-10 | 2.67E-10 |
| rs12029988 | 0.004079882 | 0.000639935 | 1.82E-10 | 2.36E-10 |
| rs12076635 | 0.004099332 | 0.000641997 | 1.71E-10 | 2.36E-10 |
| rs12132451 | 0.004130284 | 0.000640463 | 1.13E-10 | 2.36E-10 |
| rs12468040 | 0.004020913 | 0.000637506 | 2.84E-10 | 2.97E-10 |
| rs12474895 | 0.004050489 | 0.000637293 | 2.07E-10 | 2.36E-10 |
| rs12503522 | 0.004167334 | 0.000635794 | 5.58E-11 | 2.36E-10 |
| rs12532494 | 0.004178186 | 0.000638234 | 5.89E-11 | 2.36E-10 |
| rs12574281 | 0.004081565 | 0.000639672 | 1.76E-10 | 2.36E-10 |
| rs12663818 | 0.004029359 | 0.000633824 | 2.05E-10 | 2.36E-10 |
| rs12735232 | 0.004099561 | 0.000640499 | 1.55E-10 | 2.36E-10 |
| rs12804787 | 0.004120907 | 0.000640007 | 1.20E-10 | 2.36E-10 |
| rs12921005 | 0.004148908 | 0.000638154 | 7.96E-11 | 2.36E-10 |
| rs12967855 | 0.004173773 | 0.000641779 | 7.85E-11 | 2.36E-10 |
| rs1334297 | 0.004033368 | 0.000640465 | 3.02E-10 | 3.13E-10 |
| rs13409451 | 0.004165053 | 0.000639553 | 7.39E-11 | 2.36E-10 |
| rs1363862 | 0.004062588 | 0.000638079 | 1.93E-10 | 2.36E-10 |
| rs1369128 | 0.004067427 | 0.000639267 | 1.98E-10 | 2.36E-10 |
| rs1381247 | 0.004132398 | 0.000639463 | 1.03E-10 | 2.36E-10 |
| rs1391438 | 0.004070423 | 0.000640913 | 2.14E-10 | 2.37E-10 |
| rs1452075 | 0.004086067 | 0.000639925 | 1.71E-10 | 2.36E-10 |
| rs145590108 | 0.004227178 | 0.000627174 | 1.58E-11 | 2.36E-10 |
| rs1566085 | 0.004142869 | 0.000641275 | 1.04E-10 | 2.36E-10 |
| rs1569266 | 0.004135425 | 0.000639651 | 1.01E-10 | 2.36E-10 |
| rs1620977 | 0.004018266 | 0.00063877 | 3.16E-10 | 3.22E-10 |
| rs1689510 | 0.004127038 | 0.000640862 | 1.20E-10 | 2.36E-10 |
| rs17489649 | 0.004143427 | 0.000638933 | 8.88E-11 | 2.36E-10 |
| rs17513684 | 0.004073265 | 0.000639273 | 1.87E-10 | 2.36E-10 |
| rs175325 | 0.004035174 | 0.000635602 | 2.17E-10 | 2.37E-10 |
| rs17563464 | 0.004103532 | 0.00064095 | 1.53E-10 | 2.36E-10 |
| rs17628095 | 0.00407555 | 0.000639622 | 1.87E-10 | 2.36E-10 |
| rs1788783 | 0.004074916 | 0.000640249 | 1.96E-10 | 2.36E-10 |
| rs1812587 | 0.004177581 | 0.000634986 | 4.74E-11 | 2.36E-10 |
| rs1835340 | 0.004135239 | 0.00063936 | 9.95E-11 | 2.36E-10 |
| rs185291 | 0.00402946 | 0.000641133 | 3.28E-10 | 3.33E-10 |
| rs1869165 | 0.004149442 | 0.000638265 | 7.97E-11 | 2.36E-10 |
| rs1880692 | 0.004081544 | 0.000639615 | 1.76E-10 | 2.36E-10 |
| rs1892417 | 0.004084348 | 0.000640507 | 1.81E-10 | 2.36E-10 |
| rs1917008 | 0.004144957 | 0.000638537 | 8.51E-11 | 2.36E-10 |
| rs192436652 | 0.004061978 | 0.00063871 | 2.02E-10 | 2.36E-10 |
| rs1964927 | 0.004134166 | 0.000639845 | 1.04E-10 | 2.36E-10 |
| rs1980251 | 0.004017599 | 0.000638212 | 3.07E-10 | 3.16E-10 |
| rs2145265 | 0.004082809 | 0.000639748 | 1.75E-10 | 2.36E-10 |
| rs215632 | 0.004076934 | 0.000639457 | 1.82E-10 | 2.36E-10 |
| rs2175420 | 0.004125294 | 0.000640145 | 1.16E-10 | 2.36E-10 |
| rs2182398 | 0.004103353 | 0.000640277 | 1.47E-10 | 2.36E-10 |
| rs2190872 | 0.004118857 | 0.000640142 | 1.24E-10 | 2.36E-10 |
| rs2287838 | 0.004110858 | 0.000640298 | 1.36E-10 | 2.36E-10 |
| rs2299098 | 0.004142901 | 0.000640425 | 9.87E-11 | 2.36E-10 |
| rs2309812 | 0.004196544 | 0.000638852 | 5.07E-11 | 2.36E-10 |
| rs2332818 | 0.004045983 | 0.000636147 | 2.02E-10 | 2.36E-10 |
| rs2411453 | 0.004051101 | 0.00064004 | 2.46E-10 | 2.63E-10 |
| rs2559509 | 0.004126752 | 0.000640096 | 1.14E-10 | 2.36E-10 |
| rs2570497 | 0.004144375 | 0.000639417 | 9.08E-11 | 2.36E-10 |
| rs2604541 | 0.004094517 | 0.000640138 | 1.59E-10 | 2.36E-10 |
| rs2706762 | 0.004095029 | 0.000640368 | 1.61E-10 | 2.36E-10 |
| rs2725371 | 0.004109842 | 0.000640903 | 1.43E-10 | 2.36E-10 |
| rs2735421 | 0.004090929 | 0.00064171 | 1.83E-10 | 2.36E-10 |
| rs281324 | 0.004156985 | 0.000637503 | 7.00E-11 | 2.36E-10 |
| rs2820313 | 0.004124414 | 0.000639992 | 1.16E-10 | 2.36E-10 |
| rs2834011 | 0.004100056 | 0.0006404 | 1.53E-10 | 2.36E-10 |
| rs2974312 | 0.004099525 | 0.000640847 | 1.58E-10 | 2.36E-10 |
| rs2998309 | 0.004098239 | 0.000640189 | 1.54E-10 | 2.36E-10 |
| rs324801 | 0.004109996 | 0.000640335 | 1.38E-10 | 2.36E-10 |
| rs333078 | 0.004158528 | 0.000637258 | 6.77E-11 | 2.36E-10 |
| rs34042385 | 0.004140651 | 0.00063894 | 9.14E-11 | 2.36E-10 |
| rs34192341 | 0.004152912 | 0.000638142 | 7.62E-11 | 2.36E-10 |
| rs34364916 | 0.004109939 | 0.000640309 | 1.37E-10 | 2.36E-10 |
| rs34470581 | 0.004085209 | 0.000640345 | 1.77E-10 | 2.36E-10 |
| rs34945223 | 0.004102773 | 0.000640338 | 1.48E-10 | 2.36E-10 |
| rs35039375 | 0.004105255 | 0.000640674 | 1.48E-10 | 2.36E-10 |
| rs35091253 | 0.004095772 | 0.000641087 | 1.67E-10 | 2.36E-10 |
| rs35811586 | 0.004104089 | 0.000640303 | 1.46E-10 | 2.36E-10 |
| rs35917528 | 0.004119984 | 0.000640194 | 1.23E-10 | 2.36E-10 |
| rs35999162 | 0.004020723 | 0.000643555 | 4.17E-10 | 4.19E-10 |
| rs363096 | 0.004081176 | 0.000640153 | 1.83E-10 | 2.36E-10 |
| rs3747631 | 0.003946295 | 0.000630125 | 3.78E-10 | 3.82E-10 |
| rs3788556 | 0.004066152 | 0.000639383 | 2.02E-10 | 2.36E-10 |
| rs3794620 | 0.004083614 | 0.000640196 | 1.79E-10 | 2.36E-10 |
| rs3800925 | 0.004066102 | 0.000639926 | 2.10E-10 | 2.37E-10 |
| rs3825083 | 0.004064465 | 0.000639125 | 2.03E-10 | 2.36E-10 |
| rs3827531 | 0.004104902 | 0.000640302 | 1.45E-10 | 2.36E-10 |
| rs3847225 | 0.004050261 | 0.000640443 | 2.55E-10 | 2.70E-10 |
| rs3943093 | 0.00409861 | 0.000641286 | 1.65E-10 | 2.36E-10 |
| rs4130477 | 0.004055802 | 0.000637352 | 1.97E-10 | 2.36E-10 |
| rs4146675 | 0.004055859 | 0.000637392 | 1.98E-10 | 2.36E-10 |
| rs417968 | 0.004189212 | 0.000637741 | 5.07E-11 | 2.36E-10 |
| rs42210 | 0.004115022 | 0.000640204 | 1.30E-10 | 2.36E-10 |
| rs4246167 | 0.004146573 | 0.0006397 | 9.05E-11 | 2.36E-10 |
| rs4700393 | 0.004062965 | 0.000643338 | 2.69E-10 | 2.83E-10 |
| rs4726070 | 0.004109007 | 0.000640663 | 1.42E-10 | 2.36E-10 |
| rs4731992 | 0.004187129 | 0.000637853 | 5.22E-11 | 2.36E-10 |
| rs4757957 | 0.004100243 | 0.000640564 | 1.54E-10 | 2.36E-10 |
| rs4780563 | 0.004122147 | 0.000640111 | 1.20E-10 | 2.36E-10 |
| rs4808766 | 0.004035286 | 0.000634366 | 2.00E-10 | 2.36E-10 |
| rs4958568 | 0.004056736 | 0.000638047 | 2.04E-10 | 2.36E-10 |
| rs55800473 | 0.004083507 | 0.000640169 | 1.78E-10 | 2.36E-10 |
| rs55842281 | 0.004144744 | 0.000639138 | 8.88E-11 | 2.36E-10 |
| rs55859553 | 0.004113917 | 0.000640289 | 1.32E-10 | 2.36E-10 |
| rs55872852 | 0.004062018 | 0.000638188 | 1.95E-10 | 2.36E-10 |
| rs56118554 | 0.004171971 | 0.0006387 | 6.49E-11 | 2.36E-10 |
| rs575113 | 0.004102232 | 0.000640276 | 1.48E-10 | 2.36E-10 |
| rs59123361 | 0.004156764 | 0.000639019 | 7.77E-11 | 2.36E-10 |
| rs6071573 | 0.00410169 | 0.000640827 | 1.55E-10 | 2.36E-10 |
| rs613872 | 0.004068705 | 0.000639989 | 2.05E-10 | 2.36E-10 |
| rs61787087 | 0.004098896 | 0.000640189 | 1.53E-10 | 2.36E-10 |
| rs61787785 | 0.004147472 | 0.000639117 | 8.62E-11 | 2.36E-10 |
| rs61868084 | 0.004113004 | 0.000640386 | 1.34E-10 | 2.36E-10 |
| rs62018215 | 0.004104722 | 0.000640291 | 1.45E-10 | 2.36E-10 |
| rs62182125 | 0.004149035 | 0.000638084 | 7.91E-11 | 2.36E-10 |
| rs62184483 | 0.004174066 | 0.000639164 | 6.55E-11 | 2.36E-10 |
| rs62253608 | 0.004072568 | 0.000639463 | 1.91E-10 | 2.36E-10 |
| rs62389638 | 0.004211733 | 0.000632518 | 2.76E-11 | 2.36E-10 |
| rs6429911 | 0.004129446 | 0.000640065 | 1.11E-10 | 2.36E-10 |
| rs6556982 | 0.004124198 | 0.000639906 | 1.16E-10 | 2.36E-10 |
| rs660001 | 0.004058341 | 0.000639207 | 2.17E-10 | 2.37E-10 |
| rs6682095 | 0.004056675 | 0.000638796 | 2.15E-10 | 2.37E-10 |
| rs66844142 | 0.004107783 | 0.000640295 | 1.40E-10 | 2.36E-10 |
| rs6760772 | 0.004144269 | 0.000638695 | 8.66E-11 | 2.36E-10 |
| rs67651814 | 0.004084519 | 0.000640293 | 1.78E-10 | 2.36E-10 |
| rs6779254 | 0.004111701 | 0.000640824 | 1.40E-10 | 2.36E-10 |
| rs6789699 | 0.004044207 | 0.000636889 | 2.15E-10 | 2.37E-10 |
| rs67944653 | 0.004075035 | 0.000639472 | 1.86E-10 | 2.36E-10 |
| rs6935954 | 0.00396679 | 0.000636969 | 4.74E-10 | 4.74E-10 |
| rs6959579 | 0.004138065 | 0.000639167 | 9.53E-11 | 2.36E-10 |
| rs702606 | 0.00410134 | 0.000640347 | 1.50E-10 | 2.36E-10 |
| rs7031698 | 0.00410305 | 0.00064035 | 1.48E-10 | 2.36E-10 |
| rs7070693 | 0.004159331 | 0.000639345 | 7.74E-11 | 2.36E-10 |
| rs711793 | 0.004107897 | 0.000640365 | 1.41E-10 | 2.36E-10 |
| rs71646142 | 0.004119394 | 0.000640211 | 1.24E-10 | 2.36E-10 |
| rs7195278 | 0.003986062 | 0.000630641 | 2.60E-10 | 2.75E-10 |
| rs7233920 | 0.00409081 | 0.000640689 | 1.71E-10 | 2.36E-10 |
| rs72674898 | 0.004134707 | 0.000639472 | 1.01E-10 | 2.36E-10 |
| rs72807818 | 0.004205031 | 0.000630393 | 2.55E-11 | 2.36E-10 |
| rs72828517 | 0.004122461 | 0.00064124 | 1.29E-10 | 2.36E-10 |
| rs72977992 | 0.004136631 | 0.000639205 | 9.70E-11 | 2.36E-10 |
| rs73040036 | 0.004156845 | 0.000637414 | 6.96E-11 | 2.36E-10 |
| rs73499064 | 0.004167148 | 0.000636985 | 6.07E-11 | 2.36E-10 |
| rs75033012 | 0.004192626 | 0.000633739 | 3.70E-11 | 2.36E-10 |
| rs7526112 | 0.004108415 | 0.000641025 | 1.46E-10 | 2.36E-10 |
| rs7531271 | 0.004113406 | 0.000641509 | 1.44E-10 | 2.36E-10 |
| rs75433564 | 0.00413849 | 0.00063944 | 9.67E-11 | 2.36E-10 |
| rs7548936 | 0.004100281 | 0.000641559 | 1.65E-10 | 2.36E-10 |
| rs7580304 | 0.004055453 | 0.00063719 | 1.96E-10 | 2.36E-10 |
| rs7583473 | 0.004084147 | 0.000640093 | 1.76E-10 | 2.36E-10 |
| rs7598246 | 0.004045215 | 0.000637327 | 2.19E-10 | 2.38E-10 |
| rs7629643 | 0.004113939 | 0.000640232 | 1.31E-10 | 2.36E-10 |
| rs76608582 | 0.004204273 | 0.000631114 | 2.71E-11 | 2.36E-10 |
| rs7675394 | 0.004133487 | 0.000640746 | 1.11E-10 | 2.36E-10 |
| rs76878669 | 0.004103919 | 0.000640378 | 1.47E-10 | 2.36E-10 |
| rs77025239 | 0.004104645 | 0.000640421 | 1.46E-10 | 2.36E-10 |
| rs7758776 | 0.004087844 | 0.000640121 | 1.70E-10 | 2.36E-10 |
| rs77675579 | 0.004091032 | 0.000640333 | 1.67E-10 | 2.36E-10 |
| rs7768116 | 0.004136296 | 0.000639354 | 9.83E-11 | 2.36E-10 |
| rs781289 | 0.004125058 | 0.000640535 | 1.19E-10 | 2.36E-10 |
| rs78452560 | 0.004086589 | 0.00064043 | 1.76E-10 | 2.36E-10 |
| rs7868164 | 0.004134578 | 0.000639274 | 9.96E-11 | 2.36E-10 |
| rs7868984 | 0.004075667 | 0.00064357 | 2.41E-10 | 2.60E-10 |
| rs7873964 | 0.004091137 | 0.000640336 | 1.67E-10 | 2.36E-10 |
| rs7966054 | 0.004059218 | 0.000638307 | 2.03E-10 | 2.36E-10 |
| rs7977614 | 0.004142551 | 0.000639247 | 9.15E-11 | 2.36E-10 |
| rs7987170 | 0.004101395 | 0.000640458 | 1.52E-10 | 2.36E-10 |
| rs7988201 | 0.00415745 | 0.000638306 | 7.35E-11 | 2.36E-10 |
| rs7988627 | 0.004113338 | 0.00064041 | 1.34E-10 | 2.36E-10 |
| rs79937071 | 0.004090462 | 0.000640098 | 1.65E-10 | 2.36E-10 |
| rs8008382 | 0.004082398 | 0.000639783 | 1.76E-10 | 2.36E-10 |
| rs8020034 | 0.004067429 | 0.000639831 | 2.06E-10 | 2.36E-10 |
| rs8057808 | 0.00412714 | 0.000640201 | 1.14E-10 | 2.36E-10 |
| rs807478 | 0.004079018 | 0.000639601 | 1.80E-10 | 2.36E-10 |
| rs837065 | 0.004157735 | 0.00063888 | 7.62E-11 | 2.36E-10 |
| rs868698 | 0.004091933 | 0.000640418 | 1.66E-10 | 2.36E-10 |
| rs879394 | 0.004052448 | 0.000637193 | 2.02E-10 | 2.36E-10 |
| rs9372625 | 0.004042069 | 0.000642465 | 3.14E-10 | 3.22E-10 |
| rs9643120 | 0.004096419 | 0.000640604 | 1.61E-10 | 2.36E-10 |
| rs9797233 | 0.004157979 | 0.000637146 | 6.76E-11 | 2.36E-10 |
| rs9888796 | 0.004155118 | 0.00063827 | 7.52E-11 | 2.36E-10 |
| All | 0.004105915 | 0.000637739 | 1.21E-10 | 2.36E-10 |
