# Supplementary material for: Causal roles of educational duration in bone mineral density and risk factors for osteoporosis: a Mendelian randomization study
Source: BMC Musculoskelet Disord. 2024 May 2;25:345. doi: 10.1186/s12891-024-07428-8 (PMC11064366; doi:10.1186/s12891-024-07428-8)
Supplement: Supplementary file 1 — Supplementary Material 1. [file 12891_2024_7428_MOESM1_ESM.zip › IVs of Educational attainment on coffee.docx]

| SNP | b | se | P.value | adjust P.value |
| --- | --- | --- | --- | --- |
| rs10058365 | 0.019235262 | 0.002582108 | 9.37E-14 | 1.62E-13 |
| rs10066409 | 0.019164753 | 0.002578967 | 1.08E-13 | 1.62E-13 |
| rs1010334 | 0.01931824 | 0.002572964 | 6.00E-14 | 1.62E-13 |
| rs10189857 | 0.019168148 | 0.002582841 | 1.16E-13 | 1.62E-13 |
| rs10215082 | 0.019285548 | 0.002575802 | 7.04E-14 | 1.62E-13 |
| rs1050847 | 0.019570081 | 0.002539661 | 1.30E-14 | 1.62E-13 |
| rs10511592 | 0.019321637 | 0.002574421 | 6.13E-14 | 1.62E-13 |
| rs10518019 | 0.0190417 | 0.002579117 | 1.55E-13 | 1.72E-13 |
| rs10745789 | 0.019189411 | 0.002578293 | 9.87E-14 | 1.62E-13 |
| rs10760023 | 0.019348968 | 0.002569811 | 5.10E-14 | 1.62E-13 |
| rs10765775 | 0.019112825 | 0.002581975 | 1.34E-13 | 1.62E-13 |
| rs10844179 | 0.019108737 | 0.002577547 | 1.23E-13 | 1.62E-13 |
| rs10854884 | 0.019189969 | 0.002582242 | 1.07E-13 | 1.62E-13 |
| rs10994777 | 0.019233598 | 0.002580151 | 9.02E-14 | 1.62E-13 |
| rs11138947 | 0.019139043 | 0.002578125 | 1.14E-13 | 1.62E-13 |
| rs11155821 | 0.019019028 | 0.002576097 | 1.55E-13 | 1.72E-13 |
| rs11214468 | 0.019598015 | 0.002537724 | 1.14E-14 | 1.62E-13 |
| rs11243838 | 0.019252112 | 0.002577231 | 8.01E-14 | 1.62E-13 |
| rs11249939 | 0.019594262 | 0.002556526 | 1.80E-14 | 1.62E-13 |
| rs11572842 | 0.019120876 | 0.002577055 | 1.17E-13 | 1.62E-13 |
| rs115877304 | 0.019150282 | 0.002578635 | 1.11E-13 | 1.62E-13 |
| rs11604034 | 0.019241669 | 0.002579374 | 8.66E-14 | 1.62E-13 |
| rs11635966 | 0.019257329 | 0.002578023 | 8.03E-14 | 1.62E-13 |
| rs11661305 | 0.019318186 | 0.002575776 | 6.38E-14 | 1.62E-13 |
| rs11678980 | 0.019376221 | 0.002580009 | 5.91E-14 | 1.62E-13 |
| rs11690224 | 0.019105898 | 0.002576944 | 1.22E-13 | 1.62E-13 |
| rs11693764 | 0.019112952 | 0.002577227 | 1.21E-13 | 1.62E-13 |
| rs11714679 | 0.019259433 | 0.00257674 | 7.76E-14 | 1.62E-13 |
| rs11720121 | 0.018715554 | 0.002547225 | 2.02E-13 | 2.10E-13 |
| rs11732657 | 0.019208115 | 0.002578043 | 9.29E-14 | 1.62E-13 |
| rs11736863 | 0.01919104 | 0.002580874 | 1.04E-13 | 1.62E-13 |
| rs11764590 | 0.019083234 | 0.002578095 | 1.34E-13 | 1.62E-13 |
| rs117799466 | 0.019337805 | 0.00257212 | 5.55E-14 | 1.62E-13 |
| rs118083122 | 0.019454283 | 0.002559149 | 2.92E-14 | 1.62E-13 |
| rs11871429 | 0.019222396 | 0.002578643 | 9.02E-14 | 1.62E-13 |
| rs11915747 | 0.019196283 | 0.002583613 | 1.09E-13 | 1.62E-13 |
| rs12029988 | 0.019171538 | 0.002579249 | 1.06E-13 | 1.62E-13 |
| rs12076635 | 0.01859342 | 0.002547708 | 2.92E-13 | 2.97E-13 |
| rs12132451 | 0.019254482 | 0.002579596 | 8.38E-14 | 1.62E-13 |
| rs12468040 | 0.019014956 | 0.002579224 | 1.68E-13 | 1.80E-13 |
| rs12474895 | 0.019086769 | 0.002576586 | 1.28E-13 | 1.62E-13 |
| rs12503522 | 0.019435836 | 0.00255871 | 3.06E-14 | 1.62E-13 |
| rs12532494 | 0.018895285 | 0.002570472 | 1.97E-13 | 2.06E-13 |
| rs12574281 | 0.019177609 | 0.002578349 | 1.02E-13 | 1.62E-13 |
| rs12663818 | 0.019200828 | 0.002578267 | 9.54E-14 | 1.62E-13 |
| rs12735232 | 0.01937539 | 0.002570176 | 4.75E-14 | 1.62E-13 |
| rs12804787 | 0.01917912 | 0.002578104 | 1.01E-13 | 1.62E-13 |
| rs12921005 | 0.019163021 | 0.002578126 | 1.06E-13 | 1.62E-13 |
| rs12967855 | 0.018709383 | 0.00257299 | 3.56E-13 | 3.59E-13 |
| rs1334297 | 0.018705011 | 0.002565709 | 3.09E-13 | 3.14E-13 |
| rs13409451 | 0.019396969 | 0.002576475 | 5.13E-14 | 1.62E-13 |
| rs1363862 | 0.019102603 | 0.002576432 | 1.22E-13 | 1.62E-13 |
| rs1369128 | 0.019460368 | 0.002561785 | 3.04E-14 | 1.62E-13 |
| rs1381247 | 0.019200275 | 0.002578027 | 9.50E-14 | 1.62E-13 |
| rs1391438 | 0.018766257 | 0.002561184 | 2.35E-13 | 2.41E-13 |
| rs1452075 | 0.018973841 | 0.002566931 | 1.45E-13 | 1.66E-13 |
| rs145590108 | 0.019059768 | 0.002576175 | 1.38E-13 | 1.62E-13 |
| rs1566085 | 0.018868257 | 0.002573684 | 2.28E-13 | 2.35E-13 |
| rs1569266 | 0.019106578 | 0.002577725 | 1.24E-13 | 1.62E-13 |
| rs1620977 | 0.019169979 | 0.002585306 | 1.22E-13 | 1.62E-13 |
| rs1689510 | 0.018986365 | 0.00257573 | 1.69E-13 | 1.80E-13 |
| rs17489649 | 0.019333472 | 0.002572466 | 5.67E-14 | 1.62E-13 |
| rs17513684 | 0.019316749 | 0.002573656 | 6.12E-14 | 1.62E-13 |
| rs175325 | 0.019237121 | 0.002578182 | 8.55E-14 | 1.62E-13 |
| rs17563464 | 0.019144233 | 0.002580627 | 1.19E-13 | 1.62E-13 |
| rs17628095 | 0.019075706 | 0.002576483 | 1.32E-13 | 1.62E-13 |
| rs1788783 | 0.019155565 | 0.002580669 | 1.15E-13 | 1.62E-13 |
| rs1812587 | 0.01906943 | 0.002575471 | 1.32E-13 | 1.62E-13 |
| rs1835340 | 0.01924207 | 0.002577236 | 8.26E-14 | 1.62E-13 |
| rs185291 | 0.018264533 | 0.00252164 | 4.38E-13 | 4.41E-13 |
| rs1869165 | 0.019118785 | 0.002577415 | 1.19E-13 | 1.62E-13 |
| rs1880692 | 0.019086452 | 0.00257574 | 1.26E-13 | 1.62E-13 |
| rs1892417 | 0.019191794 | 0.002580418 | 1.03E-13 | 1.62E-13 |
| rs1917008 | 0.019287268 | 0.002574881 | 6.86E-14 | 1.62E-13 |
| rs192436652 | 0.019374632 | 0.002570179 | 4.76E-14 | 1.62E-13 |
| rs1964927 | 0.019070347 | 0.002576612 | 1.35E-13 | 1.62E-13 |
| rs1980251 | 0.018978958 | 0.002579785 | 1.88E-13 | 1.98E-13 |
| rs2145265 | 0.01920144 | 0.002578246 | 9.51E-14 | 1.62E-13 |
| rs215632 | 0.019435535 | 0.002560786 | 3.21E-14 | 1.62E-13 |
| rs2175420 | 0.019303259 | 0.002575375 | 6.61E-14 | 1.62E-13 |
| rs2182398 | 0.019042279 | 0.002572675 | 1.34E-13 | 1.62E-13 |
| rs2190872 | 0.019129198 | 0.00257763 | 1.16E-13 | 1.62E-13 |
| rs2287838 | 0.019036692 | 0.002572607 | 1.36E-13 | 1.62E-13 |
| rs2299098 | 0.019165029 | 0.002581999 | 1.15E-13 | 1.62E-13 |
| rs2309812 | 0.019351531 | 0.002582901 | 6.77E-14 | 1.62E-13 |
| rs2332818 | 0.019161886 | 0.002578123 | 1.07E-13 | 1.62E-13 |
| rs2411453 | 0.019334457 | 0.002580338 | 6.73E-14 | 1.62E-13 |
| rs2559509 | 0.019324346 | 0.002574059 | 6.03E-14 | 1.62E-13 |
| rs2570497 | 0.019256429 | 0.002578469 | 8.13E-14 | 1.62E-13 |
| rs2604541 | 0.019077073 | 0.002575136 | 1.28E-13 | 1.62E-13 |
| rs2706762 | 0.019138131 | 0.002578547 | 1.15E-13 | 1.62E-13 |
| rs2725371 | 0.019211765 | 0.002580496 | 9.70E-14 | 1.62E-13 |
| rs2735421 | 0.019198108 | 0.002584272 | 1.10E-13 | 1.62E-13 |
| rs281324 | 0.019326051 | 0.002572611 | 5.81E-14 | 1.62E-13 |
| rs2820313 | 0.019030462 | 0.002572481 | 1.39E-13 | 1.62E-13 |
| rs2834011 | 0.019049899 | 0.002574493 | 1.37E-13 | 1.62E-13 |
| rs2974312 | 0.019651496 | 0.002539109 | 9.98E-15 | 1.62E-13 |
| rs2998309 | 0.019040912 | 0.002572323 | 1.34E-13 | 1.62E-13 |
| rs324801 | 0.019075727 | 0.002575491 | 1.30E-13 | 1.62E-13 |
| rs333078 | 0.019108137 | 0.002576971 | 1.22E-13 | 1.62E-13 |
| rs34042385 | 0.019200769 | 0.002578147 | 9.51E-14 | 1.62E-13 |
| rs34192341 | 0.019159148 | 0.002578602 | 1.09E-13 | 1.62E-13 |
| rs34364916 | 0.019072057 | 0.002575114 | 1.30E-13 | 1.62E-13 |
| rs34470581 | 0.019320674 | 0.002575706 | 6.33E-14 | 1.62E-13 |
| rs34945223 | 0.018937021 | 0.00256227 | 1.46E-13 | 1.66E-13 |
| rs35039375 | 0.019149909 | 0.002579544 | 1.14E-13 | 1.62E-13 |
| rs35091253 | 0.019179391 | 0.002581646 | 1.09E-13 | 1.62E-13 |
| rs35811586 | 0.019298874 | 0.002574194 | 6.53E-14 | 1.62E-13 |
| rs35917528 | 0.019334861 | 0.002572311 | 5.62E-14 | 1.62E-13 |
| rs35999162 | 0.019450424 | 0.00259361 | 6.41E-14 | 1.62E-13 |
| rs363096 | 0.019223745 | 0.0025793 | 9.12E-14 | 1.62E-13 |
| rs3747631 | 0.019511991 | 0.002571973 | 3.29E-14 | 1.62E-13 |
| rs3788556 | 0.019149745 | 0.002579644 | 1.14E-13 | 1.62E-13 |
| rs3794620 | 0.018961576 | 0.002569216 | 1.58E-13 | 1.73E-13 |
| rs3800925 | 0.019107926 | 0.002580218 | 1.31E-13 | 1.62E-13 |
| rs3825083 | 0.01907352 | 0.002577106 | 1.35E-13 | 1.62E-13 |
| rs3827531 | 0.019099759 | 0.002576415 | 1.23E-13 | 1.62E-13 |
| rs3847225 | 0.0192566 | 0.002583851 | 9.15E-14 | 1.62E-13 |
| rs3943093 | 0.019220163 | 0.002582073 | 9.79E-14 | 1.62E-13 |
| rs4130477 | 0.019074238 | 0.002574962 | 1.29E-13 | 1.62E-13 |
| rs4146675 | 0.019124355 | 0.00257733 | 1.17E-13 | 1.62E-13 |
| rs417968 | 0.019348288 | 0.002579189 | 6.30E-14 | 1.62E-13 |
| rs42210 | 0.01905778 | 0.002573973 | 1.32E-13 | 1.62E-13 |
| rs4246167 | 0.019510865 | 0.002560684 | 2.55E-14 | 1.62E-13 |
| rs4700393 | 0.019193799 | 0.002592393 | 1.32E-13 | 1.62E-13 |
| rs4726070 | 0.018832003 | 0.002554108 | 1.67E-13 | 1.80E-13 |
| rs4731992 | 0.019456573 | 0.002572479 | 3.93E-14 | 1.62E-13 |
| rs4757957 | 0.019349149 | 0.002572944 | 5.47E-14 | 1.62E-13 |
| rs4780563 | 0.019479332 | 0.002554808 | 2.45E-14 | 1.62E-13 |
| rs4808766 | 0.019267542 | 0.002575839 | 7.43E-14 | 1.62E-13 |
| rs4958568 | 0.019369264 | 0.002569931 | 4.81E-14 | 1.62E-13 |
| rs55800473 | 0.019238253 | 0.002578713 | 8.63E-14 | 1.62E-13 |
| rs55842281 | 0.019269252 | 0.002577335 | 7.64E-14 | 1.62E-13 |
| rs55859553 | 0.018952306 | 0.002564442 | 1.46E-13 | 1.66E-13 |
| rs55872852 | 0.019253878 | 0.002576825 | 7.90E-14 | 1.62E-13 |
| rs56118554 | 0.018818827 | 0.00256301 | 2.10E-13 | 2.17E-13 |
| rs575113 | 0.019116293 | 0.00257703 | 1.19E-13 | 1.62E-13 |
| rs59123361 | 0.019002347 | 0.00257497 | 1.59E-13 | 1.73E-13 |
| rs6071573 | 0.019277584 | 0.002578574 | 7.66E-14 | 1.62E-13 |
| rs613872 | 0.019326933 | 0.00257708 | 6.40E-14 | 1.62E-13 |
| rs61787087 | 0.018796067 | 0.0025333 | 1.17E-13 | 1.62E-13 |
| rs61787785 | 0.019070629 | 0.002576974 | 1.36E-13 | 1.62E-13 |
| rs61868084 | 0.01949591 | 0.002553226 | 2.24E-14 | 1.62E-13 |
| rs62018215 | 0.019462578 | 0.0025549 | 2.58E-14 | 1.62E-13 |
| rs62182125 | 0.019158519 | 0.002578005 | 1.07E-13 | 1.62E-13 |
| rs62184483 | 0.019016506 | 0.002579925 | 1.69E-13 | 1.80E-13 |
| rs62253608 | 0.019261673 | 0.002577504 | 7.84E-14 | 1.62E-13 |
| rs62389638 | 0.019242351 | 0.002579811 | 8.73E-14 | 1.62E-13 |
| rs6429911 | 0.019070565 | 0.002576614 | 1.35E-13 | 1.62E-13 |
| rs6556982 | 0.01919257 | 0.00257814 | 9.74E-14 | 1.62E-13 |
| rs660001 | 0.019422315 | 0.002569698 | 4.09E-14 | 1.62E-13 |
| rs6682095 | 0.019317067 | 0.002576235 | 6.47E-14 | 1.62E-13 |
| rs66844142 | 0.019037193 | 0.002572403 | 1.36E-13 | 1.62E-13 |
| rs6760772 | 0.018901798 | 0.002556957 | 1.44E-13 | 1.66E-13 |
| rs67651814 | 0.01912294 | 0.00257907 | 1.22E-13 | 1.62E-13 |
| rs6779254 | 0.019392571 | 0.002571896 | 4.69E-14 | 1.62E-13 |
| rs6789699 | 0.019315575 | 0.002574712 | 6.28E-14 | 1.62E-13 |
| rs67944653 | 0.019148583 | 0.002578542 | 1.12E-13 | 1.62E-13 |
| rs6935954 | 0.019632551 | 0.002573327 | 2.36E-14 | 1.62E-13 |
| rs6959579 | 0.019277234 | 0.00257566 | 7.19E-14 | 1.62E-13 |
| rs702606 | 0.019177295 | 0.002578496 | 1.03E-13 | 1.62E-13 |
| rs7031698 | 0.019308673 | 0.002573926 | 6.30E-14 | 1.62E-13 |
| rs7070693 | 0.019211399 | 0.002581493 | 9.92E-14 | 1.62E-13 |
| rs711793 | 0.019257878 | 0.002576836 | 7.81E-14 | 1.62E-13 |
| rs71646142 | 0.019208045 | 0.002578412 | 9.36E-14 | 1.62E-13 |
| rs7195278 | 0.018934754 | 0.002570212 | 1.75E-13 | 1.85E-13 |
| rs7233920 | 0.019283598 | 0.002578447 | 7.50E-14 | 1.62E-13 |
| rs72674898 | 0.019269523 | 0.002576313 | 7.46E-14 | 1.62E-13 |
| rs72807818 | 0.019040916 | 0.002573951 | 1.39E-13 | 1.62E-13 |
| rs72828517 | 0.019155363 | 0.002582544 | 1.20E-13 | 1.62E-13 |
| rs72977992 | 0.019423307 | 0.002561079 | 3.35E-14 | 1.62E-13 |
| rs73040036 | 0.019182331 | 0.002578337 | 1.01E-13 | 1.62E-13 |
| rs73499064 | 0.019188555 | 0.002579127 | 1.01E-13 | 1.62E-13 |
| rs75033012 | 0.019333677 | 0.002574001 | 5.86E-14 | 1.62E-13 |
| rs7526112 | 0.018896021 | 0.002567587 | 1.85E-13 | 1.95E-13 |
| rs7531271 | 0.019145606 | 0.002583056 | 1.24E-13 | 1.62E-13 |
| rs75433564 | 0.019091115 | 0.002577044 | 1.28E-13 | 1.62E-13 |
| rs7548936 | 0.019255339 | 0.002582604 | 8.93E-14 | 1.62E-13 |
| rs7580304 | 0.019327895 | 0.002571473 | 5.64E-14 | 1.62E-13 |
| rs7583473 | 0.019256101 | 0.002577797 | 8.02E-14 | 1.62E-13 |
| rs7598246 | 0.019322498 | 0.002574935 | 6.19E-14 | 1.62E-13 |
| rs7629643 | 0.019239851 | 0.002577203 | 8.30E-14 | 1.62E-13 |
| rs76608582 | 0.019269946 | 0.002577064 | 7.58E-14 | 1.62E-13 |
| rs7675394 | 0.0191004 | 0.00258094 | 1.36E-13 | 1.62E-13 |
| rs76878669 | 0.019199139 | 0.002578447 | 9.62E-14 | 1.62E-13 |
| rs77025239 | 0.019075778 | 0.00257596 | 1.31E-13 | 1.62E-13 |
| rs7758776 | 0.019330001 | 0.002573141 | 5.81E-14 | 1.62E-13 |
| rs77675579 | 0.019282082 | 0.002576715 | 7.25E-14 | 1.62E-13 |
| rs7768116 | 0.019117779 | 0.002577434 | 1.19E-13 | 1.62E-13 |
| rs781289 | 0.019149989 | 0.002580147 | 1.15E-13 | 1.62E-13 |
| rs78452560 | 0.018979857 | 0.002571922 | 1.59E-13 | 1.73E-13 |
| rs7868164 | 0.019241373 | 0.002576905 | 8.21E-14 | 1.62E-13 |
| rs7868984 | 0.01933828 | 0.002590679 | 8.36E-14 | 1.62E-13 |
| rs7873964 | 0.018958302 | 0.002567791 | 1.55E-13 | 1.72E-13 |
| rs7966054 | 0.019290237 | 0.002575881 | 6.95E-14 | 1.62E-13 |
| rs7977614 | 0.019041708 | 0.002574642 | 1.40E-13 | 1.64E-13 |
| rs7987170 | 0.019308467 | 0.002574859 | 6.44E-14 | 1.62E-13 |
| rs7988201 | 0.019108375 | 0.00257845 | 1.26E-13 | 1.62E-13 |
| rs7988627 | 0.019358451 | 0.002570943 | 5.09E-14 | 1.62E-13 |
| rs79937071 | 0.019177918 | 0.002578452 | 1.02E-13 | 1.62E-13 |
| rs8008382 | 0.019062518 | 0.00257488 | 1.33E-13 | 1.62E-13 |
| rs8020034 | 0.019065984 | 0.002578329 | 1.42E-13 | 1.64E-13 |
| rs8057808 | 0.018802146 | 0.002547687 | 1.58E-13 | 1.73E-13 |
| rs807478 | 0.019283459 | 0.002575637 | 7.05E-14 | 1.62E-13 |
| rs837065 | 0.019406447 | 0.002571001 | 4.41E-14 | 1.62E-13 |
| rs868698 | 0.019151439 | 0.002579214 | 1.13E-13 | 1.62E-13 |
| rs879394 | 0.019141142 | 0.002578007 | 1.13E-13 | 1.62E-13 |
| rs9372625 | 0.018573564 | 0.002566104 | 4.55E-13 | 4.55E-13 |
| rs9643120 | 0.019128106 | 0.002579195 | 1.20E-13 | 1.62E-13 |
| rs9797233 | 0.019309563 | 0.00257332 | 6.20E-14 | 1.62E-13 |
| rs9888796 | 0.018825651 | 0.002549947 | 1.55E-13 | 1.72E-13 |
| All | 0.019179418 | 0.002567901 | 8.09E-14 | 1.62E-13 |
