# Supplementary material for: Causal roles of educational duration in bone mineral density and risk factors for osteoporosis: a Mendelian randomization study
Source: BMC Musculoskelet Disord. 2024 May 2;25:345. doi: 10.1186/s12891-024-07428-8 (PMC11064366; doi:10.1186/s12891-024-07428-8)
Supplement: Supplementary file 1 — Supplementary Material 1. [file 12891_2024_7428_MOESM1_ESM.zip › IVs of Educational attainment on falling risk.docx]

| SNP | b | se | P.value | adjust P.value |
| --- | --- | --- | --- | --- |
| rs10058365 | -0.024744107 | 0.005835226 | 2.23E-05 | 4.70E-05 |
| rs10066409 | -0.024309264 | 0.005839504 | 3.14E-05 | 4.70E-05 |
| rs1010334 | -0.024182646 | 0.005836326 | 3.42E-05 | 4.70E-05 |
| rs10189857 | -0.024570743 | 0.005843891 | 2.62E-05 | 4.70E-05 |
| rs10215082 | -0.024195976 | 0.005837573 | 3.40E-05 | 4.70E-05 |
| rs1050847 | -0.024788274 | 0.005812075 | 2.00E-05 | 4.70E-05 |
| rs10511592 | -0.024070024 | 0.005834221 | 3.70E-05 | 4.70E-05 |
| rs10518019 | -0.023815607 | 0.005830478 | 4.41E-05 | 4.74E-05 |
| rs10745789 | -0.024619442 | 0.005825448 | 2.38E-05 | 4.70E-05 |
| rs10760023 | -0.024613364 | 0.005825016 | 2.38E-05 | 4.70E-05 |
| rs10765775 | -0.023992083 | 0.005841719 | 4.01E-05 | 4.70E-05 |
| rs10844179 | -0.024612669 | 0.005828352 | 2.41E-05 | 4.70E-05 |
| rs10854884 | -0.024958669 | 0.00581895 | 1.79E-05 | 4.70E-05 |
| rs10994777 | -0.023742438 | 0.005818466 | 4.49E-05 | 4.74E-05 |
| rs11138947 | -0.024038449 | 0.005830417 | 3.74E-05 | 4.70E-05 |
| rs11155821 | -0.024193056 | 0.005842451 | 3.46E-05 | 4.70E-05 |
| rs11214468 | -0.024308444 | 0.005839813 | 3.15E-05 | 4.70E-05 |
| rs11243838 | -0.024122671 | 0.005834968 | 3.56E-05 | 4.70E-05 |
| rs11249939 | -0.0241855 | 0.005845748 | 3.51E-05 | 4.70E-05 |
| rs11572842 | -0.024346842 | 0.005837098 | 3.03E-05 | 4.70E-05 |
| rs115877304 | -0.024451574 | 0.005836761 | 2.80E-05 | 4.70E-05 |
| rs11604034 | -0.024877524 | 0.005813323 | 1.87E-05 | 4.70E-05 |
| rs11635966 | -0.024449857 | 0.005838215 | 2.82E-05 | 4.70E-05 |
| rs11661305 | -0.024384439 | 0.005840644 | 2.98E-05 | 4.70E-05 |
| rs11678980 | -0.024370711 | 0.005851637 | 3.12E-05 | 4.70E-05 |
| rs11690224 | -0.024509934 | 0.005832962 | 2.65E-05 | 4.70E-05 |
| rs11693764 | -0.02409873 | 0.005833272 | 3.61E-05 | 4.70E-05 |
| rs11714679 | -0.02480419 | 0.005808181 | 1.95E-05 | 4.70E-05 |
| rs11720121 | -0.023549714 | 0.005805667 | 4.98E-05 | 5.06E-05 |
| rs11732657 | -0.024461765 | 0.005834641 | 2.76E-05 | 4.70E-05 |
| rs11736863 | -0.024473797 | 0.005841492 | 2.79E-05 | 4.70E-05 |
| rs11764590 | -0.02334871 | 0.005759647 | 5.04E-05 | 5.09E-05 |
| rs117799466 | -0.024433251 | 0.005836747 | 2.84E-05 | 4.70E-05 |
| rs118083122 | -0.024274327 | 0.005838744 | 3.22E-05 | 4.70E-05 |
| rs11871429 | -0.024123362 | 0.005836384 | 3.58E-05 | 4.70E-05 |
| rs11915747 | -0.024641963 | 0.005843689 | 2.48E-05 | 4.70E-05 |
| rs12029988 | -0.023963566 | 0.005828592 | 3.93E-05 | 4.70E-05 |
| rs12076635 | -0.023835008 | 0.00584276 | 4.52E-05 | 4.74E-05 |
| rs12132451 | -0.024931154 | 0.005811265 | 1.79E-05 | 4.70E-05 |
| rs12468040 | -0.024050166 | 0.005844555 | 3.87E-05 | 4.70E-05 |
| rs12474895 | -0.024221368 | 0.005838268 | 3.34E-05 | 4.70E-05 |
| rs12503522 | -0.023950544 | 0.005821576 | 3.89E-05 | 4.70E-05 |
| rs12532494 | -0.024736272 | 0.005834712 | 2.24E-05 | 4.70E-05 |
| rs12574281 | -0.024416328 | 0.005836447 | 2.87E-05 | 4.70E-05 |
| rs12663818 | -0.023919352 | 0.005820473 | 3.96E-05 | 4.70E-05 |
| rs12735232 | -0.024510823 | 0.005835374 | 2.66E-05 | 4.70E-05 |
| rs12804787 | -0.023920639 | 0.005818875 | 3.94E-05 | 4.70E-05 |
| rs12921005 | -0.024509315 | 0.005832218 | 2.64E-05 | 4.70E-05 |
| rs12967855 | -0.024533665 | 0.005862195 | 2.85E-05 | 4.70E-05 |
| rs1334297 | -0.023846784 | 0.005848934 | 4.56E-05 | 4.74E-05 |
| rs13409451 | -0.023809607 | 0.005833888 | 4.48E-05 | 4.74E-05 |
| rs1363862 | -0.023875114 | 0.005814083 | 4.02E-05 | 4.70E-05 |
| rs1369128 | -0.024267355 | 0.005840353 | 3.25E-05 | 4.70E-05 |
| rs1381247 | -0.023939073 | 0.005820745 | 3.91E-05 | 4.70E-05 |
| rs1391438 | -0.024908299 | 0.005828122 | 1.92E-05 | 4.70E-05 |
| rs1452075 | -0.025133575 | 0.00575433 | 1.26E-05 | 4.70E-05 |
| rs145590108 | -0.024061119 | 0.005834867 | 3.73E-05 | 4.70E-05 |
| rs1566085 | -0.024535904 | 0.005849557 | 2.73E-05 | 4.70E-05 |
| rs1569266 | -0.024498935 | 0.005835356 | 2.69E-05 | 4.70E-05 |
| rs1620977 | -0.025489511 | 0.005787021 | 1.06E-05 | 4.70E-05 |
| rs1689510 | -0.02369598 | 0.005819892 | 4.67E-05 | 4.81E-05 |
| rs17489649 | -0.024542118 | 0.00583206 | 2.57E-05 | 4.70E-05 |
| rs17513684 | -0.024124518 | 0.005834996 | 3.56E-05 | 4.70E-05 |
| rs175325 | -0.024163626 | 0.005837447 | 3.48E-05 | 4.70E-05 |
| rs17563464 | -0.024107806 | 0.005840815 | 3.67E-05 | 4.70E-05 |
| rs17628095 | -0.024231434 | 0.005839132 | 3.33E-05 | 4.70E-05 |
| rs1788783 | -0.024497011 | 0.005840502 | 2.74E-05 | 4.70E-05 |
| rs1812587 | -0.024136252 | 0.00583554 | 3.53E-05 | 4.70E-05 |
| rs1835340 | -0.024154227 | 0.005835237 | 3.48E-05 | 4.70E-05 |
| rs185291 | -0.02615543 | 0.00574012 | 5.20E-06 | 4.70E-05 |
| rs1869165 | -0.024335964 | 0.005838105 | 3.07E-05 | 4.70E-05 |
| rs1880692 | -0.02459241 | 0.005827009 | 2.44E-05 | 4.70E-05 |
| rs1892417 | -0.024258914 | 0.005842683 | 3.30E-05 | 4.70E-05 |
| rs1917008 | -0.024396203 | 0.005836516 | 2.92E-05 | 4.70E-05 |
| rs192436652 | -0.024570861 | 0.005832444 | 2.52E-05 | 4.70E-05 |
| rs1964927 | -0.02407606 | 0.005835149 | 3.69E-05 | 4.70E-05 |
| rs1980251 | -0.025076167 | 0.005820877 | 1.65E-05 | 4.70E-05 |
| rs2145265 | -0.024604875 | 0.005826769 | 2.41E-05 | 4.70E-05 |
| rs215632 | -0.023657045 | 0.005788726 | 4.37E-05 | 4.74E-05 |
| rs2175420 | -0.024472227 | 0.005836517 | 2.75E-05 | 4.70E-05 |
| rs2182398 | -0.024031869 | 0.005828253 | 3.73E-05 | 4.70E-05 |
| rs2190872 | -0.024093918 | 0.005832639 | 3.61E-05 | 4.70E-05 |
| rs2287838 | -0.024299492 | 0.005837959 | 3.15E-05 | 4.70E-05 |
| rs2299098 | -0.024666158 | 0.00583755 | 2.38E-05 | 4.70E-05 |
| rs2309812 | -0.024013371 | 0.005851465 | 4.06E-05 | 4.70E-05 |
| rs2332818 | -0.02405885 | 0.005830257 | 3.68E-05 | 4.70E-05 |
| rs2411453 | -0.024906353 | 0.005828798 | 1.93E-05 | 4.70E-05 |
| rs2559509 | -0.024040217 | 0.005832516 | 3.76E-05 | 4.70E-05 |
| rs2570497 | -0.02511827 | 0.005778654 | 1.38E-05 | 4.70E-05 |
| rs2604541 | -0.02430001 | 0.005837627 | 3.15E-05 | 4.70E-05 |
| rs2706762 | -0.023999198 | 0.005829633 | 3.84E-05 | 4.70E-05 |
| rs2725371 | -0.02371911 | 0.005816338 | 4.54E-05 | 4.74E-05 |
| rs2735421 | -0.024891026 | 0.005833585 | 1.98E-05 | 4.70E-05 |
| rs281324 | -0.02422718 | 0.00583761 | 3.32E-05 | 4.70E-05 |
| rs2820313 | -0.024349919 | 0.005838049 | 3.03E-05 | 4.70E-05 |
| rs2834011 | -0.024166603 | 0.005836914 | 3.47E-05 | 4.70E-05 |
| rs2974312 | -0.024247202 | 0.00584276 | 3.33E-05 | 4.70E-05 |
| rs2998309 | -0.024443282 | 0.00583472 | 2.80E-05 | 4.70E-05 |
| rs324801 | -0.024066993 | 0.00583162 | 3.68E-05 | 4.70E-05 |
| rs333078 | -0.02416553 | 0.005835876 | 3.46E-05 | 4.70E-05 |
| rs34042385 | -0.024303729 | 0.005837885 | 3.14E-05 | 4.70E-05 |
| rs34192341 | -0.023952146 | 0.005824938 | 3.92E-05 | 4.70E-05 |
| rs34364916 | -0.024725406 | 0.005815865 | 2.12E-05 | 4.70E-05 |
| rs34470581 | -0.02440213 | 0.005840482 | 2.94E-05 | 4.70E-05 |
| rs34945223 | -0.024209479 | 0.005837162 | 3.36E-05 | 4.70E-05 |
| rs35039375 | -0.024454567 | 0.005839089 | 2.81E-05 | 4.70E-05 |
| rs35091253 | -0.024623944 | 0.005838267 | 2.47E-05 | 4.70E-05 |
| rs35811586 | -0.024672519 | 0.005820543 | 2.25E-05 | 4.70E-05 |
| rs35917528 | -0.024394388 | 0.005837686 | 2.93E-05 | 4.70E-05 |
| rs35999162 | -0.024652785 | 0.005878203 | 2.74E-05 | 4.70E-05 |
| rs363096 | -0.023886926 | 0.005825158 | 4.12E-05 | 4.70E-05 |
| rs3747631 | -0.026013984 | 0.005703399 | 5.09E-06 | 4.70E-05 |
| rs3788556 | -0.024968076 | 0.005800569 | 1.67E-05 | 4.70E-05 |
| rs3794620 | -0.024016179 | 0.005832935 | 3.83E-05 | 4.70E-05 |
| rs3800925 | -0.024097411 | 0.005841067 | 3.70E-05 | 4.70E-05 |
| rs3825083 | -0.0252638 | 0.005751184 | 1.12E-05 | 4.70E-05 |
| rs3827531 | -0.024292589 | 0.005837756 | 3.16E-05 | 4.70E-05 |
| rs3847225 | -0.024887783 | 0.005834695 | 1.99E-05 | 4.70E-05 |
| rs3943093 | -0.023998489 | 0.005841093 | 3.98E-05 | 4.70E-05 |
| rs4130477 | -0.024194831 | 0.005836191 | 3.39E-05 | 4.70E-05 |
| rs4146675 | -0.024602479 | 0.005826202 | 2.41E-05 | 4.70E-05 |
| rs417968 | -0.022947322 | 0.005738905 | 6.37E-05 | 6.40E-05 |
| rs42210 | -0.024685816 | 0.005818969 | 2.21E-05 | 4.70E-05 |
| rs4246167 | -0.023891611 | 0.005829841 | 4.16E-05 | 4.70E-05 |
| rs4700393 | -0.025323654 | 0.005839664 | 1.45E-05 | 4.70E-05 |
| rs4726070 | -0.023701282 | 0.005807458 | 4.48E-05 | 4.74E-05 |
| rs4731992 | -0.024466304 | 0.005846688 | 2.86E-05 | 4.70E-05 |
| rs4757957 | -0.024359422 | 0.005840073 | 3.03E-05 | 4.70E-05 |
| rs4780563 | -0.024284336 | 0.005838525 | 3.19E-05 | 4.70E-05 |
| rs4808766 | -0.024871205 | 0.005795236 | 1.77E-05 | 4.70E-05 |
| rs4958568 | -0.024274904 | 0.005839155 | 3.22E-05 | 4.70E-05 |
| rs55800473 | -0.024103887 | 0.005836804 | 3.63E-05 | 4.70E-05 |
| rs55842281 | -0.023888429 | 0.005822503 | 4.08E-05 | 4.70E-05 |
| rs55859553 | -0.024655589 | 0.005823279 | 2.30E-05 | 4.70E-05 |
| rs55872852 | -0.024257374 | 0.005837974 | 3.25E-05 | 4.70E-05 |
| rs56118554 | -0.023818061 | 0.005831699 | 4.42E-05 | 4.74E-05 |
| rs575113 | -0.023800865 | 0.005805781 | 4.14E-05 | 4.70E-05 |
| rs59123361 | -0.024149441 | 0.005841373 | 3.56E-05 | 4.70E-05 |
| rs6071573 | -0.024826613 | 0.005819681 | 1.99E-05 | 4.70E-05 |
| rs613872 | -0.02373472 | 0.005818899 | 4.52E-05 | 4.74E-05 |
| rs61787087 | -0.024479174 | 0.005833059 | 2.71E-05 | 4.70E-05 |
| rs61787785 | -0.023536834 | 0.005784868 | 4.73E-05 | 4.82E-05 |
| rs61868084 | -0.024466286 | 0.005835957 | 2.76E-05 | 4.70E-05 |
| rs62018215 | -0.023847621 | 0.005811289 | 4.07E-05 | 4.70E-05 |
| rs62182125 | -0.024429684 | 0.005835419 | 2.83E-05 | 4.70E-05 |
| rs62184483 | -0.024113471 | 0.005847456 | 3.73E-05 | 4.70E-05 |
| rs62253608 | -0.024411391 | 0.005838508 | 2.90E-05 | 4.70E-05 |
| rs62389638 | -0.023953395 | 0.005832965 | 4.02E-05 | 4.70E-05 |
| rs6429911 | -0.024717774 | 0.005822982 | 2.19E-05 | 4.70E-05 |
| rs6556982 | -0.02433688 | 0.005837531 | 3.06E-05 | 4.70E-05 |
| rs660001 | -0.023853289 | 0.005826735 | 4.24E-05 | 4.71E-05 |
| rs6682095 | -0.023622458 | 0.005800735 | 4.65E-05 | 4.81E-05 |
| rs66844142 | -0.024327246 | 0.005837541 | 3.08E-05 | 4.70E-05 |
| rs6760772 | -0.024971153 | 0.005783031 | 1.57E-05 | 4.70E-05 |
| rs67651814 | -0.02403486 | 0.005834552 | 3.80E-05 | 4.70E-05 |
| rs6779254 | -0.024496218 | 0.005839605 | 2.73E-05 | 4.70E-05 |
| rs6789699 | -0.024301947 | 0.005839784 | 3.16E-05 | 4.70E-05 |
| rs67944653 | -0.024524357 | 0.005833435 | 2.62E-05 | 4.70E-05 |
| rs6935954 | -0.023052889 | 0.005809187 | 7.24E-05 | 7.24E-05 |
| rs6959579 | -0.023812205 | 0.005808451 | 4.14E-05 | 4.70E-05 |
| rs702606 | -0.024618774 | 0.005826362 | 2.39E-05 | 4.70E-05 |
| rs7031698 | -0.024251435 | 0.005838018 | 3.27E-05 | 4.70E-05 |
| rs7070693 | -0.024082505 | 0.005842135 | 3.75E-05 | 4.70E-05 |
| rs711793 | -0.024401418 | 0.005837197 | 2.91E-05 | 4.70E-05 |
| rs71646142 | -0.024429515 | 0.005836783 | 2.85E-05 | 4.70E-05 |
| rs7195278 | -0.0249711 | 0.005808466 | 1.72E-05 | 4.70E-05 |
| rs7233920 | -0.023947808 | 0.005832585 | 4.03E-05 | 4.70E-05 |
| rs72674898 | -0.024365188 | 0.005837865 | 3.00E-05 | 4.70E-05 |
| rs72807818 | -0.02395398 | 0.005825694 | 3.93E-05 | 4.70E-05 |
| rs72828517 | -0.023916886 | 0.005838675 | 4.20E-05 | 4.70E-05 |
| rs72977992 | -0.023746273 | 0.005798238 | 4.21E-05 | 4.70E-05 |
| rs73040036 | -0.024439889 | 0.005835685 | 2.81E-05 | 4.70E-05 |
| rs73499064 | -0.024766742 | 0.005817196 | 2.07E-05 | 4.70E-05 |
| rs75033012 | -0.024134387 | 0.005837712 | 3.56E-05 | 4.70E-05 |
| rs7526112 | -0.023887862 | 0.005831711 | 4.20E-05 | 4.70E-05 |
| rs7531271 | -0.024698064 | 0.005839937 | 2.35E-05 | 4.70E-05 |
| rs75433564 | -0.023911921 | 0.005823002 | 4.02E-05 | 4.70E-05 |
| rs7548936 | -0.024065162 | 0.005846208 | 3.85E-05 | 4.70E-05 |
| rs7580304 | -0.024429924 | 0.005835163 | 2.83E-05 | 4.70E-05 |
| rs7583473 | -0.024275409 | 0.005839859 | 3.23E-05 | 4.70E-05 |
| rs7598246 | -0.024164388 | 0.005838745 | 3.49E-05 | 4.70E-05 |
| rs7629643 | -0.024136172 | 0.005834291 | 3.52E-05 | 4.70E-05 |
| rs76608582 | -0.024061567 | 0.00583368 | 3.71E-05 | 4.70E-05 |
| rs7675394 | -0.024291972 | 0.00584608 | 3.25E-05 | 4.70E-05 |
| rs76878669 | -0.024841172 | 0.005804163 | 1.87E-05 | 4.70E-05 |
| rs77025239 | -0.024197374 | 0.005837648 | 3.40E-05 | 4.70E-05 |
| rs7758776 | -0.023735301 | 0.005803595 | 4.32E-05 | 4.74E-05 |
| rs77675579 | -0.023941023 | 0.005826316 | 3.97E-05 | 4.70E-05 |
| rs7768116 | -0.024758403 | 0.005813106 | 2.05E-05 | 4.70E-05 |
| rs781289 | -0.024423798 | 0.005841264 | 2.90E-05 | 4.70E-05 |
| rs78452560 | -0.024343473 | 0.005841624 | 3.08E-05 | 4.70E-05 |
| rs7868164 | -0.024415084 | 0.005835634 | 2.87E-05 | 4.70E-05 |
| rs7868984 | -0.024428663 | 0.005869275 | 3.15E-05 | 4.70E-05 |
| rs7873964 | -0.024762165 | 0.00581809 | 2.08E-05 | 4.70E-05 |
| rs7966054 | -0.023737055 | 0.005804595 | 4.33E-05 | 4.74E-05 |
| rs7977614 | -0.024062888 | 0.005833848 | 3.71E-05 | 4.70E-05 |
| rs7987170 | -0.024147127 | 0.005836814 | 3.52E-05 | 4.70E-05 |
| rs7988201 | -0.023534551 | 0.005784048 | 4.72E-05 | 4.82E-05 |
| rs7988627 | -0.024232641 | 0.00583873 | 3.32E-05 | 4.70E-05 |
| rs79937071 | -0.02447708 | 0.005834612 | 2.73E-05 | 4.70E-05 |
| rs8008382 | -0.024781127 | 0.005811235 | 2.00E-05 | 4.70E-05 |
| rs8020034 | -0.024495613 | 0.00584016 | 2.74E-05 | 4.70E-05 |
| rs8057808 | -0.024203253 | 0.005839563 | 3.40E-05 | 4.70E-05 |
| rs807478 | -0.024068581 | 0.005832041 | 3.68E-05 | 4.70E-05 |
| rs837065 | -0.024203779 | 0.005842215 | 3.43E-05 | 4.70E-05 |
| rs868698 | -0.02451444 | 0.005835907 | 2.66E-05 | 4.70E-05 |
| rs879394 | -0.024183468 | 0.005836542 | 3.42E-05 | 4.70E-05 |
| rs9372625 | -0.024229405 | 0.005867585 | 3.64E-05 | 4.70E-05 |
| rs9643120 | -0.024122367 | 0.005838272 | 3.60E-05 | 4.70E-05 |
| rs9797233 | -0.023624234 | 0.005779217 | 4.35E-05 | 4.74E-05 |
| rs9888796 | -0.024044285 | 0.005833124 | 3.76E-05 | 4.70E-05 |
| All | -0.024301184 | 0.005814384 | 2.92E-05 | 4.70E-05 |
