# Supplementary material for: Causal roles of educational duration in bone mineral density and risk factors for osteoporosis: a Mendelian randomization study
Source: BMC Musculoskelet Disord. 2024 May 2;25:345. doi: 10.1186/s12891-024-07428-8 (PMC11064366; doi:10.1186/s12891-024-07428-8)
Supplement: Supplementary file 1 — Supplementary Material 1. [file 12891_2024_7428_MOESM1_ESM.zip › IVs of Educational attainment on fizzy drink.docx]

| SNP | b | se | P.value | adjust P.value |
| --- | --- | --- | --- | --- |
| rs10058365 | -0.013838972 | 0.003778538 | 0.000249746 | 0.000253612 |
| rs10066409 | -0.014681101 | 0.003773248 | 9.99E-05 | 0.000192608 |
| rs1010334 | -0.014228561 | 0.003772084 | 0.0001619 | 0.000195016 |
| rs10189857 | -0.014032112 | 0.003778934 | 0.000204618 | 0.000216895 |
| rs10215082 | -0.014362673 | 0.003772735 | 0.000140682 | 0.000192608 |
| rs1050847 | -0.014797937 | 0.003772893 | 8.78E-05 | 0.000192608 |
| rs10511592 | -0.014268276 | 0.003773471 | 0.00015607 | 0.000192608 |
| rs10518019 | -0.013899256 | 0.00377761 | 0.000233804 | 0.000240614 |
| rs10745789 | -0.014353122 | 0.003772221 | 0.000141831 | 0.000192608 |
| rs10760023 | -0.014692368 | 0.003772018 | 9.82E-05 | 0.000192608 |
| rs10765775 | -0.014403573 | 0.003778498 | 0.000137856 | 0.000192608 |
| rs10844179 | -0.014649697 | 0.003772908 | 0.000103233 | 0.000192608 |
| rs10854884 | -0.014851077 | 0.003778044 | 8.46E-05 | 0.000192608 |
| rs10994777 | -0.0148227 | 0.003775702 | 8.64E-05 | 0.000192608 |
| rs11138947 | -0.014408502 | 0.003772559 | 0.000133836 | 0.000192608 |
| rs11155821 | -0.014459253 | 0.003775676 | 0.000128362 | 0.000192608 |
| rs11214468 | -0.014450057 | 0.003773427 | 0.000128443 | 0.000192608 |
| rs11243838 | -0.014367577 | 0.003772665 | 0.000139905 | 0.000192608 |
| rs11249939 | -0.014961881 | 0.003777791 | 7.48E-05 | 0.000192608 |
| rs11572842 | -0.01458777 | 0.003771862 | 0.000109945 | 0.000192608 |
| rs115877304 | -0.014139131 | 0.003773066 | 0.000178688 | 0.000202829 |
| rs11604034 | -0.014657383 | 0.003774975 | 0.000103271 | 0.000192608 |
| rs11635966 | -0.014694398 | 0.003773847 | 9.87E-05 | 0.000192608 |
| rs11661305 | -0.014717152 | 0.003774466 | 9.65E-05 | 0.000192608 |
| rs11678980 | -0.015336818 | 0.003781177 | 4.99E-05 | 0.000192608 |
| rs11690224 | -0.014373775 | 0.003772346 | 0.000138798 | 0.000192608 |
| rs11693764 | -0.014252103 | 0.00377238 | 0.000158084 | 0.000192608 |
| rs11714679 | -0.01446857 | 0.003772382 | 0.000125363 | 0.000192608 |
| rs11720121 | -0.014503591 | 0.003777003 | 0.000123047 | 0.000192608 |
| rs11732657 | -0.014169792 | 0.003772176 | 0.000172377 | 0.00020051 |
| rs11736863 | -0.014282262 | 0.003775975 | 0.000155321 | 0.000192608 |
| rs11764590 | -0.014391738 | 0.003774766 | 0.000137503 | 0.000192608 |
| rs117799466 | -0.014309798 | 0.003772722 | 0.00014886 | 0.000192608 |
| rs118083122 | -0.014648843 | 0.003772882 | 0.000103317 | 0.000192608 |
| rs11871429 | -0.014249305 | 0.003773368 | 0.000159186 | 0.000192843 |
| rs11915747 | -0.014896694 | 0.003780018 | 8.12E-05 | 0.000192608 |
| rs12029988 | -0.013910669 | 0.003773602 | 0.000227529 | 0.000238792 |
| rs12076635 | -0.014600053 | 0.003782149 | 0.000113269 | 0.000192608 |
| rs12132451 | -0.014909129 | 0.003775643 | 7.86E-05 | 0.000192608 |
| rs12468040 | -0.014847119 | 0.003778895 | 8.53E-05 | 0.000192608 |
| rs12474895 | -0.014768046 | 0.003772931 | 9.07E-05 | 0.000192608 |
| rs12503522 | -0.014766154 | 0.003771927 | 9.05E-05 | 0.000192608 |
| rs12532494 | -0.014572801 | 0.00377803 | 0.000114671 | 0.000192608 |
| rs12574281 | -0.014643061 | 0.003772282 | 0.000103707 | 0.000192608 |
| rs12663818 | -0.014306353 | 0.003772322 | 0.000149167 | 0.000192608 |
| rs12735232 | -0.014538147 | 0.003773442 | 0.000116796 | 0.000192608 |
| rs12804787 | -0.014662434 | 0.003771882 | 0.000101366 | 0.000192608 |
| rs12921005 | -0.014361962 | 0.003772075 | 0.000140411 | 0.000192608 |
| rs12967855 | -0.013719292 | 0.003788992 | 0.00029366 | 0.00029366 |
| rs1334297 | -0.014403692 | 0.003784635 | 0.000141327 | 0.000192608 |
| rs13409451 | -0.014210446 | 0.003778872 | 0.000169575 | 0.000198935 |
| rs1363862 | -0.014405419 | 0.003771937 | 0.000133938 | 0.000192608 |
| rs1369128 | -0.01436582 | 0.003773671 | 0.000140745 | 0.000192608 |
| rs1381247 | -0.014483769 | 0.003772004 | 0.000123131 | 0.000192608 |
| rs1391438 | -0.014432818 | 0.003779651 | 0.000134234 | 0.000192608 |
| rs1452075 | -0.014648713 | 0.003772407 | 0.000103124 | 0.000192608 |
| rs145590108 | -0.014333507 | 0.003773673 | 0.000145697 | 0.000192608 |
| rs1566085 | -0.014091101 | 0.003781557 | 0.000194335 | 0.000208076 |
| rs1569266 | -0.014257197 | 0.003773284 | 0.000157802 | 0.000192608 |
| rs1620977 | -0.014708476 | 0.00378261 | 0.000100888 | 0.000192608 |
| rs1689510 | -0.01429253 | 0.003777066 | 0.000154309 | 0.000192608 |
| rs17489649 | -0.014662429 | 0.00377267 | 0.000101706 | 0.000192608 |
| rs17513684 | -0.014342596 | 0.003772744 | 0.000143744 | 0.000192608 |
| rs175325 | -0.014492237 | 0.003773191 | 0.000122612 | 0.000192608 |
| rs17563464 | -0.014538442 | 0.003775957 | 0.000117989 | 0.000192608 |
| rs17628095 | -0.01453955 | 0.003773264 | 0.000116533 | 0.000192608 |
| rs1788783 | -0.014126165 | 0.003775844 | 0.000183148 | 0.000204355 |
| rs1812587 | -0.014386993 | 0.003772704 | 0.000137044 | 0.000192608 |
| rs1835340 | -0.014412583 | 0.003772239 | 0.000133075 | 0.000192608 |
| rs185291 | -0.014616065 | 0.003788025 | 0.000114088 | 0.000192608 |
| rs1869165 | -0.014627216 | 0.003772331 | 0.000105535 | 0.000192608 |
| rs1880692 | -0.014696076 | 0.003772099 | 9.78E-05 | 0.000192608 |
| rs1892417 | -0.014439575 | 0.003775345 | 0.00013093 | 0.000192608 |
| rs1917008 | -0.01456064 | 0.003772109 | 0.00011335 | 0.000192608 |
| rs192436652 | -0.01421337 | 0.003773424 | 0.000165417 | 0.000197448 |
| rs1964927 | -0.014132885 | 0.003773719 | 0.000180335 | 0.000203357 |
| rs1980251 | -0.014861132 | 0.003781336 | 8.49E-05 | 0.000192608 |
| rs2145265 | -0.014732682 | 0.003772324 | 9.40E-05 | 0.000192608 |
| rs215632 | -0.014184815 | 0.003772457 | 0.000169845 | 0.000198935 |
| rs2175420 | -0.014518321 | 0.003773371 | 0.000119294 | 0.000192608 |
| rs2182398 | -0.01468288 | 0.003771998 | 9.92E-05 | 0.000192608 |
| rs2190872 | -0.014603921 | 0.003772246 | 0.000108206 | 0.000192608 |
| rs2287838 | -0.014491166 | 0.003772243 | 0.000122272 | 0.000192608 |
| rs2299098 | -0.014090156 | 0.003777616 | 0.00019155 | 0.000206135 |
| rs2309812 | -0.014955188 | 0.003783386 | 7.72E-05 | 0.000192608 |
| rs2332818 | -0.014105104 | 0.003772091 | 0.000184509 | 0.000204393 |
| rs2411453 | -0.014108672 | 0.003779615 | 0.000189338 | 0.000204794 |
| rs2559509 | -0.014749263 | 0.003773355 | 9.28E-05 | 0.000192608 |
| rs2570497 | -0.014635327 | 0.003774354 | 0.000105504 | 0.000192608 |
| rs2604541 | -0.013841707 | 0.003772032 | 0.000242966 | 0.000248835 |
| rs2706762 | -0.014412519 | 0.00377319 | 0.000133604 | 0.000192608 |
| rs2725371 | -0.014127518 | 0.003775657 | 0.000182752 | 0.000204355 |
| rs2735421 | -0.014120652 | 0.00378102 | 0.000188003 | 0.000204393 |
| rs281324 | -0.014369531 | 0.003772418 | 0.000139472 | 0.000192608 |
| rs2820313 | -0.014789659 | 0.003772349 | 8.83E-05 | 0.000192608 |
| rs2834011 | -0.014413993 | 0.003772897 | 0.000133233 | 0.000192608 |
| rs2974312 | -0.01462668 | 0.003775462 | 0.000107001 | 0.000192608 |
| rs2998309 | -0.014446267 | 0.00377184 | 0.000128127 | 0.000192608 |
| rs324801 | -0.014356241 | 0.003772429 | 0.000141478 | 0.000192608 |
| rs333078 | -0.014595765 | 0.003772302 | 0.000109196 | 0.000192608 |
| rs34042385 | -0.014481525 | 0.003772172 | 0.000123515 | 0.000192608 |
| rs34192341 | -0.014606842 | 0.003772905 | 0.000108163 | 0.000192608 |
| rs34364916 | -0.014427544 | 0.003772157 | 0.000130907 | 0.000192608 |
| rs34470581 | -0.014591093 | 0.003774488 | 0.000110764 | 0.000192608 |
| rs34945223 | -0.014526543 | 0.003772312 | 0.000117717 | 0.000192608 |
| rs35039375 | -0.01414186 | 0.003774108 | 0.00017891 | 0.000202829 |
| rs35091253 | -0.013832654 | 0.003777106 | 0.000250023 | 0.000253612 |
| rs35811586 | -0.014745042 | 0.003772047 | 9.27E-05 | 0.000192608 |
| rs35917528 | -0.014424167 | 0.003772644 | 0.000131647 | 0.000192608 |
| rs35999162 | -0.015295983 | 0.003800348 | 5.70E-05 | 0.000192608 |
| rs363096 | -0.014372515 | 0.003774247 | 0.000140068 | 0.000192608 |
| rs3747631 | -0.014227774 | 0.003781585 | 0.000168301 | 0.000198935 |
| rs3788556 | -0.014336126 | 0.003774442 | 0.000145745 | 0.000192608 |
| rs3794620 | -0.014546271 | 0.00377404 | 0.000116063 | 0.000192608 |
| rs3800925 | -0.014660446 | 0.003776154 | 0.000103441 | 0.000192608 |
| rs3825083 | -0.014396453 | 0.003774033 | 0.0001364 | 0.000192608 |
| rs3827531 | -0.014390755 | 0.003772137 | 0.000136175 | 0.000192608 |
| rs3847225 | -0.013838407 | 0.003781326 | 0.00025254 | 0.000254945 |
| rs3943093 | -0.014600397 | 0.00377804 | 0.000111297 | 0.000192608 |
| rs4130477 | -0.014699717 | 0.003771984 | 9.74E-05 | 0.000192608 |
| rs4146675 | -0.014510919 | 0.003772052 | 0.000119594 | 0.000192608 |
| rs417968 | -0.014127227 | 0.003779074 | 0.000185285 | 0.000204393 |
| rs42210 | -0.01439638 | 0.003772135 | 0.000135354 | 0.000192608 |
| rs4246167 | -0.014276224 | 0.003775621 | 0.0001561 | 0.000192608 |
| rs4700393 | -0.014633818 | 0.003792841 | 0.000114191 | 0.000192608 |
| rs4726070 | -0.014433669 | 0.00377426 | 0.000131177 | 0.000192608 |
| rs4731992 | -0.014652022 | 0.003778811 | 0.000105572 | 0.000192608 |
| rs4757957 | -0.014520032 | 0.003773794 | 0.000119283 | 0.000192608 |
| rs4780563 | -0.014269811 | 0.003772619 | 0.000155281 | 0.000192608 |
| rs4808766 | -0.014348643 | 0.003771885 | 0.000142318 | 0.000192608 |
| rs4958568 | -0.014536611 | 0.00377296 | 0.000116756 | 0.000192608 |
| rs55800473 | -0.014337732 | 0.003773881 | 0.000145163 | 0.000192608 |
| rs55842281 | -0.014600255 | 0.003773564 | 0.000109243 | 0.000192608 |
| rs55859553 | -0.014088812 | 0.003772444 | 0.000187966 | 0.000204393 |
| rs55872852 | -0.014757246 | 0.00377224 | 9.15E-05 | 0.000192608 |
| rs56118554 | -0.014422088 | 0.003777958 | 0.000134849 | 0.000192608 |
| rs575113 | -0.014595815 | 0.003772047 | 0.000109073 | 0.000192608 |
| rs59123361 | -0.014375514 | 0.003775459 | 0.00014031 | 0.000192608 |
| rs6071573 | -0.014729953 | 0.003775264 | 9.55E-05 | 0.000192608 |
| rs613872 | -0.013895467 | 0.003776051 | 0.00023333 | 0.000240614 |
| rs61787087 | -0.01444185 | 0.003771774 | 0.000128703 | 0.000192608 |
| rs61787785 | -0.014289029 | 0.003773961 | 0.000152959 | 0.000192608 |
| rs61868084 | -0.014771319 | 0.003772875 | 9.04E-05 | 0.000192608 |
| rs62018215 | -0.01480015 | 0.003772057 | 8.72E-05 | 0.000192608 |
| rs62182125 | -0.014303414 | 0.003771934 | 0.000149401 | 0.000192608 |
| rs62184483 | -0.014293228 | 0.003779577 | 0.000155761 | 0.000192608 |
| rs62253608 | -0.014269956 | 0.003773384 | 0.000155736 | 0.000192608 |
| rs62389638 | -0.014538386 | 0.00377557 | 0.000117806 | 0.000192608 |
| rs6429911 | -0.014487163 | 0.003773649 | 0.00012352 | 0.000192608 |
| rs6556982 | -0.01464718 | 0.003772031 | 0.000103132 | 0.000192608 |
| rs660001 | -0.014375405 | 0.003775405 | 0.000140296 | 0.000192608 |
| rs6682095 | -0.014329941 | 0.00377479 | 0.000146918 | 0.000192608 |
| rs66844142 | -0.014510566 | 0.003772179 | 0.000119703 | 0.000192608 |
| rs6760772 | -0.014992271 | 0.003772301 | 7.06E-05 | 0.000192608 |
| rs67651814 | -0.014533136 | 0.003774312 | 0.000117858 | 0.000192608 |
| rs6779254 | -0.014157092 | 0.003775311 | 0.000176895 | 0.000202829 |
| rs6789699 | -0.01431769 | 0.003773356 | 0.00014799 | 0.000192608 |
| rs67944653 | -0.014477314 | 0.003772895 | 0.00012445 | 0.000192608 |
| rs6935954 | -0.014668703 | 0.003788318 | 0.000107907 | 0.000192608 |
| rs6959579 | -0.014256996 | 0.003772254 | 0.000157183 | 0.000192608 |
| rs702606 | -0.014293722 | 0.00377246 | 0.000151276 | 0.000192608 |
| rs7031698 | -0.014540049 | 0.003772395 | 0.000116048 | 0.000192608 |
| rs7070693 | -0.014291079 | 0.003777252 | 0.000154664 | 0.000192608 |
| rs711793 | -0.014448883 | 0.003772479 | 0.000128104 | 0.000192608 |
| rs71646142 | -0.014415264 | 0.00377272 | 0.000132954 | 0.000192608 |
| rs7195278 | -0.013943708 | 0.003775881 | 0.000221767 | 0.000233904 |
| rs7233920 | -0.014107988 | 0.003775382 | 0.000186349 | 0.000204393 |
| rs72674898 | -0.014518364 | 0.003772533 | 0.000118873 | 0.000192608 |
| rs72807818 | -0.014331313 | 0.003773017 | 0.000145651 | 0.000192608 |
| rs72828517 | -0.014868913 | 0.00377864 | 8.32E-05 | 0.000192608 |
| rs72977992 | -0.014569845 | 0.003772078 | 0.000112209 | 0.000192608 |
| rs73040036 | -0.014137087 | 0.003772238 | 0.000178488 | 0.000202829 |
| rs73499064 | -0.014336154 | 0.003773448 | 0.000145153 | 0.000192608 |
| rs75033012 | -0.014419908 | 0.003773646 | 0.000132797 | 0.000192608 |
| rs7526112 | -0.014868209 | 0.0037763 | 8.24E-05 | 0.000192608 |
| rs7531271 | -0.013908189 | 0.003779439 | 0.000233269 | 0.000240614 |
| rs75433564 | -0.014591948 | 0.003773171 | 0.000110051 | 0.000192608 |
| rs7548936 | -0.014048363 | 0.00377961 | 0.000201697 | 0.000214873 |
| rs7580304 | -0.014835395 | 0.003771854 | 8.38E-05 | 0.000192608 |
| rs7583473 | -0.013891455 | 0.00377352 | 0.000232049 | 0.000240614 |
| rs7598246 | -0.014413467 | 0.003773875 | 0.000133844 | 0.000192608 |
| rs7629643 | -0.014551147 | 0.003772082 | 0.00011451 | 0.000192608 |
| rs76608582 | -0.014311212 | 0.003773396 | 0.000149041 | 0.000192608 |
| rs7675394 | -0.014387995 | 0.003777442 | 0.000139572 | 0.000192608 |
| rs76878669 | -0.014720087 | 0.003772612 | 9.55E-05 | 0.000192608 |
| rs77025239 | -0.014767172 | 0.003772788 | 9.07E-05 | 0.000192608 |
| rs7758776 | -0.014743804 | 0.003772961 | 9.32E-05 | 0.000192608 |
| rs77675579 | -0.014669361 | 0.003773566 | 0.000101324 | 0.000192608 |
| rs7768116 | -0.014393673 | 0.003772461 | 0.00013593 | 0.000192608 |
| rs781289 | -0.014630186 | 0.003774896 | 0.000106339 | 0.000192608 |
| rs78452560 | -0.014392395 | 0.003774452 | 0.00013723 | 0.000192608 |
| rs7868164 | -0.014682943 | 0.003771846 | 9.91E-05 | 0.000192608 |
| rs7868984 | -0.014910049 | 0.003792716 | 8.45E-05 | 0.000192608 |
| rs7873964 | -0.014470317 | 0.003773494 | 0.000125704 | 0.000192608 |
| rs7966054 | -0.014169418 | 0.003773103 | 0.000173082 | 0.00020051 |
| rs7977614 | -0.014387316 | 0.003773408 | 0.000137392 | 0.000192608 |
| rs7987170 | -0.014764074 | 0.003773075 | 9.12E-05 | 0.000192608 |
| rs7988201 | -0.014306444 | 0.00377403 | 0.000150187 | 0.000192608 |
| rs7988627 | -0.014209852 | 0.003773041 | 0.000165781 | 0.000197448 |
| rs79937071 | -0.014549794 | 0.003772379 | 0.000114821 | 0.000192608 |
| rs8008382 | -0.014468388 | 0.00377251 | 0.000125454 | 0.000192608 |
| rs8020034 | -0.013738585 | 0.003775726 | 0.00027406 | 0.000275359 |
| rs8057808 | -0.014292356 | 0.003773925 | 0.000152395 | 0.000192608 |
| rs807478 | -0.014416983 | 0.003772555 | 0.000132619 | 0.000192608 |
| rs837065 | -0.014538564 | 0.003775518 | 0.000117758 | 0.000192608 |
| rs868698 | -0.014754551 | 0.003773903 | 9.24E-05 | 0.000192608 |
| rs879394 | -0.014534776 | 0.003772354 | 0.000116692 | 0.000192608 |
| rs9372625 | -0.014648569 | 0.003791554 | 0.000111786 | 0.000192608 |
| rs9643120 | -0.014420065 | 0.003774291 | 0.000133126 | 0.000192608 |
| rs9797233 | -0.014433614 | 0.003772031 | 0.000129987 | 0.000192608 |
| rs9888796 | -0.014287257 | 0.003773522 | 0.000152977 | 0.000192608 |
| All | -0.014452528 | 0.003765968 | 0.000124209 | 0.000192608 |
