# Supplementary material for: Causal roles of educational duration in bone mineral density and risk factors for osteoporosis: a Mendelian randomization study
Source: BMC Musculoskelet Disord. 2024 May 2;25:345. doi: 10.1186/s12891-024-07428-8 (PMC11064366; doi:10.1186/s12891-024-07428-8)
Supplement: Supplementary file 1 — Supplementary Material 1. [file 12891_2024_7428_MOESM1_ESM.zip › IVs of Educational attainment on lean mass.docx]

| SNP | b | se | P.value | adjust P.value |
| --- | --- | --- | --- | --- |
| rs10058365 | -0.006668691 | 0.004446903 | 0.133711338 | 0.157249913 |
| rs10066409 | -0.00672001 | 0.004441292 | 0.130260522 | 0.157249913 |
| rs1010334 | -0.006942016 | 0.004439281 | 0.117871561 | 0.157249913 |
| rs10189857 | -0.00701272 | 0.004446523 | 0.11476696 | 0.157249913 |
| rs10215082 | -0.007230818 | 0.004417215 | 0.101638217 | 0.157249913 |
| rs1050847 | -0.006902811 | 0.004441316 | 0.120130434 | 0.157249913 |
| rs10511592 | -0.006780692 | 0.00444271 | 0.126947243 | 0.157249913 |
| rs10518019 | -0.0069325 | 0.004446792 | 0.118998967 | 0.157249913 |
| rs10745789 | -0.006813297 | 0.004441566 | 0.125033203 | 0.157249913 |
| rs10760023 | -0.006723155 | 0.00443966 | 0.129939668 | 0.157249913 |
| rs10765775 | -0.007311326 | 0.004429424 | 0.098814802 | 0.157249913 |
| rs10844179 | -0.006461884 | 0.004423576 | 0.144075007 | 0.157249913 |
| rs10854884 | -0.007275851 | 0.004430943 | 0.100578676 | 0.157249913 |
| rs10994777 | -0.006657799 | 0.004442873 | 0.133994473 | 0.157249913 |
| rs11138947 | -0.007256578 | 0.004412922 | 0.100095037 | 0.157249913 |
| rs11155821 | -0.007218831 | 0.00442933 | 0.103148069 | 0.157249913 |
| rs11214468 | -0.006737499 | 0.004442008 | 0.129325064 | 0.157249913 |
| rs11243838 | -0.006668182 | 0.004438464 | 0.133003544 | 0.157249913 |
| rs11249939 | -0.006550735 | 0.004441907 | 0.140277787 | 0.157249913 |
| rs11572842 | -0.006700996 | 0.004438576 | 0.13111553 | 0.157249913 |
| rs115877304 | -0.006965398 | 0.004439627 | 0.116667708 | 0.157249913 |
| rs11604034 | -0.00633461 | 0.004417864 | 0.15161149 | 0.161255871 |
| rs11635966 | -0.006891718 | 0.004442796 | 0.120850912 | 0.157249913 |
| rs11661305 | -0.006798101 | 0.004444043 | 0.126088349 | 0.157249913 |
| rs11678980 | -0.006944931 | 0.004451147 | 0.118699284 | 0.157249913 |
| rs11690224 | -0.006927848 | 0.004439965 | 0.11867992 | 0.157249913 |
| rs11693764 | -0.007016597 | 0.004435777 | 0.113690879 | 0.157249913 |
| rs11714679 | -0.006774078 | 0.004441491 | 0.127214001 | 0.157249913 |
| rs11720121 | -0.006914631 | 0.004446415 | 0.119921654 | 0.157249913 |
| rs11732657 | -0.006759667 | 0.004440893 | 0.127973691 | 0.157249913 |
| rs11736863 | -0.007007897 | 0.00444247 | 0.11468569 | 0.157249913 |
| rs11764590 | -0.006651243 | 0.004440877 | 0.134202926 | 0.157249913 |
| rs117799466 | -0.006884793 | 0.004441588 | 0.12112358 | 0.157249913 |
| rs118083122 | -0.006911692 | 0.004440859 | 0.119616297 | 0.157249913 |
| rs11871429 | -0.006702072 | 0.004440949 | 0.13125968 | 0.157249913 |
| rs11915747 | -0.005862325 | 0.004383766 | 0.181131102 | 0.18285616 |
| rs12029988 | -0.006727206 | 0.004442042 | 0.129914305 | 0.157249913 |
| rs12076635 | -0.007213414 | 0.004443375 | 0.104502088 | 0.157249913 |
| rs12132451 | -0.007024916 | 0.004441195 | 0.113703828 | 0.157249913 |
| rs12468040 | -0.007221758 | 0.004436842 | 0.103592841 | 0.157249913 |
| rs12474895 | -0.006874388 | 0.004441948 | 0.121717076 | 0.157249913 |
| rs12503522 | -0.006533985 | 0.004427144 | 0.139972948 | 0.157249913 |
| rs12532494 | -0.006704662 | 0.004447224 | 0.131655368 | 0.157249913 |
| rs12574281 | -0.006801849 | 0.004441601 | 0.125671617 | 0.157249913 |
| rs12663818 | -0.006638099 | 0.004436296 | 0.134571452 | 0.157249913 |
| rs12735232 | -0.007042436 | 0.004436458 | 0.112421934 | 0.157249913 |
| rs12804787 | -0.006699222 | 0.004438773 | 0.131234731 | 0.157249913 |
| rs12921005 | -0.006850503 | 0.004441259 | 0.122959689 | 0.157249913 |
| rs12967855 | -0.007604535 | 0.004434276 | 0.086355446 | 0.157249913 |
| rs1334297 | -0.006635 | 0.004454513 | 0.13635558 | 0.157249913 |
| rs13409451 | -0.006826771 | 0.004449494 | 0.124960742 | 0.157249913 |
| rs1363862 | -0.006882608 | 0.004440623 | 0.121160851 | 0.157249913 |
| rs1369128 | -0.006583944 | 0.004436126 | 0.13776513 | 0.157249913 |
| rs1381247 | -0.007296598 | 0.004403173 | 0.097494645 | 0.157249913 |
| rs1391438 | -0.007574365 | 0.00440733 | 0.08569023 | 0.157249913 |
| rs1452075 | -0.007162212 | 0.004423364 | 0.105409065 | 0.157249913 |
| rs145590108 | -0.007000153 | 0.004439569 | 0.114849859 | 0.157249913 |
| rs1566085 | -0.006193731 | 0.004426585 | 0.161749375 | 0.167272524 |
| rs1569266 | -0.007005851 | 0.004438024 | 0.114428557 | 0.157249913 |
| rs1620977 | -0.007472573 | 0.004427177 | 0.091432954 | 0.157249913 |
| rs1689510 | -0.006009669 | 0.004386582 | 0.170683198 | 0.173965567 |
| rs17489649 | -0.006752783 | 0.004441465 | 0.128411646 | 0.157249913 |
| rs17513684 | -0.006501144 | 0.004426413 | 0.141909856 | 0.157249913 |
| rs175325 | -0.006852786 | 0.004442589 | 0.122947135 | 0.157249913 |
| rs17563464 | -0.006853321 | 0.004445898 | 0.123196832 | 0.157249913 |
| rs17628095 | -0.006510273 | 0.004429379 | 0.141617646 | 0.157249913 |
| rs1788783 | -0.005879963 | 0.004353287 | 0.176793108 | 0.179330808 |
| rs1812587 | -0.006512517 | 0.004427595 | 0.141320143 | 0.157249913 |
| rs1835340 | -0.007249289 | 0.004411568 | 0.100332162 | 0.157249913 |
| rs185291 | -0.006795719 | 0.004460084 | 0.127589865 | 0.157249913 |
| rs1869165 | -0.006901382 | 0.004440811 | 0.120165018 | 0.157249913 |
| rs1880692 | -0.00695251 | 0.004438573 | 0.117258718 | 0.157249913 |
| rs1892417 | -0.006024419 | 0.004376098 | 0.168615974 | 0.172688824 |
| rs1917008 | -0.007041142 | 0.004433309 | 0.112232906 | 0.157249913 |
| rs192436652 | -0.006682125 | 0.00444049 | 0.132371147 | 0.157249913 |
| rs1964927 | -0.006636622 | 0.004438788 | 0.134877165 | 0.157249913 |
| rs1980251 | -0.007793646 | 0.004387999 | 0.07571186 | 0.157249913 |
| rs2145265 | -0.006726152 | 0.004440202 | 0.129815405 | 0.157249913 |
| rs215632 | -0.006547984 | 0.004430102 | 0.13939008 | 0.157249913 |
| rs2175420 | -0.006780502 | 0.004442606 | 0.126949022 | 0.157249913 |
| rs2182398 | -0.006701704 | 0.00443884 | 0.131097747 | 0.157249913 |
| rs2190872 | -0.00682893 | 0.004441584 | 0.124171189 | 0.157249913 |
| rs2287838 | -0.007125924 | 0.00442655 | 0.107438386 | 0.157249913 |
| rs2299098 | -0.007007317 | 0.00444493 | 0.114916618 | 0.157249913 |
| rs2309812 | -0.006309844 | 0.004439248 | 0.155207243 | 0.162088352 |
| rs2332818 | -0.006766631 | 0.004440891 | 0.127581149 | 0.157249913 |
| rs2411453 | -0.005793698 | 0.004371227 | 0.185032909 | 0.185909842 |
| rs2559509 | -0.006589291 | 0.00443541 | 0.137382241 | 0.157249913 |
| rs2570497 | -0.006588747 | 0.004437353 | 0.137586952 | 0.157249913 |
| rs2604541 | -0.006795832 | 0.004441211 | 0.125973325 | 0.157249913 |
| rs2706762 | -0.006710689 | 0.004440953 | 0.130764945 | 0.157249913 |
| rs2725371 | -0.006488416 | 0.004434056 | 0.143381432 | 0.157249913 |
| rs2735421 | -0.006890164 | 0.004451586 | 0.121670478 | 0.157249913 |
| rs281324 | -0.00693779 | 0.00443939 | 0.118104288 | 0.157249913 |
| rs2820313 | -0.006149589 | 0.004370542 | 0.159411349 | 0.165662775 |
| rs2834011 | -0.006617261 | 0.004436146 | 0.135785988 | 0.157249913 |
| rs2974312 | -0.006922461 | 0.004444331 | 0.119329642 | 0.157249913 |
| rs2998309 | -0.006639132 | 0.00443537 | 0.134429428 | 0.157249913 |
| rs324801 | -0.006726097 | 0.004440313 | 0.129828118 | 0.157249913 |
| rs333078 | -0.006626048 | 0.004435547 | 0.135214472 | 0.157249913 |
| rs34042385 | -0.006822452 | 0.004441553 | 0.124525877 | 0.157249913 |
| rs34192341 | -0.006869244 | 0.004441862 | 0.121989074 | 0.157249913 |
| rs34364916 | -0.006791574 | 0.004441482 | 0.126233832 | 0.157249913 |
| rs34470581 | -0.006512229 | 0.004432675 | 0.141794307 | 0.157249913 |
| rs34945223 | -0.006895625 | 0.004440868 | 0.120479264 | 0.157249913 |
| rs35039375 | -0.006738765 | 0.004443243 | 0.129359618 | 0.157249913 |
| rs35091253 | -0.006310192 | 0.004423216 | 0.153693143 | 0.161301714 |
| rs35811586 | -0.007074425 | 0.004431104 | 0.110368794 | 0.157249913 |
| rs35917528 | -0.006756646 | 0.004441483 | 0.128194844 | 0.157249913 |
| rs35999162 | -0.006217945 | 0.004463378 | 0.163588645 | 0.168353363 |
| rs363096 | -0.006599008 | 0.004437898 | 0.137023525 | 0.157249913 |
| rs3747631 | -0.006848739 | 0.004452372 | 0.123994255 | 0.157249913 |
| rs3788556 | -0.007167939 | 0.004429741 | 0.105632567 | 0.157249913 |
| rs3794620 | -0.007002014 | 0.004439616 | 0.114757232 | 0.157249913 |
| rs3800925 | -0.007270133 | 0.004426445 | 0.100500603 | 0.157249913 |
| rs3825083 | -0.007141289 | 0.004430766 | 0.107016289 | 0.157249913 |
| rs3827531 | -0.006642853 | 0.004436092 | 0.134274576 | 0.157249913 |
| rs3847225 | -0.006987138 | 0.004450479 | 0.116421065 | 0.157249913 |
| rs3943093 | -0.006507334 | 0.004440082 | 0.142760372 | 0.157249913 |
| rs4130477 | -0.006815845 | 0.00444131 | 0.12487044 | 0.157249913 |
| rs4146675 | -0.006882361 | 0.004440781 | 0.121187429 | 0.157249913 |
| rs417968 | -0.006664527 | 0.004447759 | 0.134029054 | 0.157249913 |
| rs42210 | -0.006758312 | 0.004440732 | 0.128036264 | 0.157249913 |
| rs4246167 | -0.006465453 | 0.004432257 | 0.14464025 | 0.157249913 |
| rs4700393 | -0.007675062 | 0.004438195 | 0.083751758 | 0.157249913 |
| rs4726070 | -0.006918106 | 0.004442827 | 0.119437116 | 0.157249913 |
| rs4731992 | -0.006723779 | 0.004448734 | 0.130688771 | 0.157249913 |
| rs4757957 | -0.006467654 | 0.004427192 | 0.144044778 | 0.157249913 |
| rs4780563 | -0.006524833 | 0.004428604 | 0.140659791 | 0.157249913 |
| rs4808766 | -0.00662237 | 0.004434422 | 0.135332283 | 0.157249913 |
| rs4958568 | -0.007233003 | 0.004418303 | 0.101619079 | 0.157249913 |
| rs55800473 | -0.006692933 | 0.004441467 | 0.131831275 | 0.157249913 |
| rs55842281 | -0.006643637 | 0.004438812 | 0.134467387 | 0.157249913 |
| rs55859553 | -0.006830416 | 0.004441838 | 0.124110853 | 0.157249913 |
| rs55872852 | -0.006673282 | 0.004438302 | 0.132693011 | 0.157249913 |
| rs56118554 | -0.007351491 | 0.004424614 | 0.096613261 | 0.157249913 |
| rs575113 | -0.007229367 | 0.004413564 | 0.101424078 | 0.157249913 |
| rs59123361 | -0.006640743 | 0.004442052 | 0.134921809 | 0.157249913 |
| rs6071573 | -0.007135216 | 0.004434434 | 0.107605937 | 0.157249913 |
| rs613872 | -0.006369525 | 0.004425278 | 0.150051305 | 0.160660993 |
| rs61787087 | -0.007121849 | 0.004425263 | 0.107537257 | 0.157249913 |
| rs61787785 | -0.006562825 | 0.004435342 | 0.138962394 | 0.157249913 |
| rs61868084 | -0.00676353 | 0.004441883 | 0.127840841 | 0.157249913 |
| rs62018215 | -0.006847333 | 0.004441231 | 0.123130748 | 0.157249913 |
| rs62182125 | -0.007103649 | 0.004427753 | 0.108637761 | 0.157249913 |
| rs62184483 | -0.006983843 | 0.004448409 | 0.11642347 | 0.157249913 |
| rs62253608 | -0.006924845 | 0.004441511 | 0.118968156 | 0.157249913 |
| rs62389638 | -0.007190594 | 0.004430726 | 0.10461229 | 0.157249913 |
| rs6429911 | -0.006705808 | 0.004441612 | 0.131102323 | 0.157249913 |
| rs6556982 | -0.006990786 | 0.004436605 | 0.115093566 | 0.157249913 |
| rs660001 | -0.006329066 | 0.00441957 | 0.15212818 | 0.161255871 |
| rs6682095 | -0.006763929 | 0.00444403 | 0.128002527 | 0.157249913 |
| rs66844142 | -0.006842234 | 0.004441176 | 0.123405545 | 0.157249913 |
| rs6760772 | -0.00693569 | 0.004439592 | 0.118232392 | 0.157249913 |
| rs67651814 | -0.006503301 | 0.0044315 | 0.142235504 | 0.157249913 |
| rs6779254 | -0.006626244 | 0.004441001 | 0.13568314 | 0.157249913 |
| rs6789699 | -0.006765572 | 0.004442523 | 0.127780661 | 0.157249913 |
| rs67944653 | -0.006928109 | 0.00444073 | 0.118729546 | 0.157249913 |
| rs6935954 | -0.005691417 | 0.004402551 | 0.19609596 | 0.19609596 |
| rs6959579 | -0.007006296 | 0.004436066 | 0.114245751 | 0.157249913 |
| rs702606 | -0.006696533 | 0.004439422 | 0.131445846 | 0.157249913 |
| rs7031698 | -0.006758234 | 0.004441225 | 0.128083053 | 0.157249913 |
| rs7070693 | -0.006582804 | 0.004442007 | 0.138355251 | 0.157249913 |
| rs711793 | -0.006904211 | 0.00444085 | 0.120016411 | 0.157249913 |
| rs71646142 | -0.006761959 | 0.004441584 | 0.127903703 | 0.157249913 |
| rs7195278 | -0.0067645 | 0.004445542 | 0.128100098 | 0.157249913 |
| rs7233920 | -0.0068066 | 0.004445279 | 0.125720598 | 0.157249913 |
| rs72674898 | -0.006279874 | 0.004395857 | 0.153122295 | 0.161301714 |
| rs72807818 | -0.006700603 | 0.004440331 | 0.131290526 | 0.157249913 |
| rs72828517 | -0.007886553 | 0.004357138 | 0.07029102 | 0.157249913 |
| rs72977992 | -0.006776402 | 0.004440966 | 0.127038559 | 0.157249913 |
| rs73040036 | -0.007115225 | 0.004427739 | 0.108061896 | 0.157249913 |
| rs73499064 | -0.006880102 | 0.004442533 | 0.121456475 | 0.157249913 |
| rs75033012 | -0.007003533 | 0.004439269 | 0.114650178 | 0.157249913 |
| rs7526112 | -0.006752835 | 0.004446029 | 0.128801175 | 0.157249913 |
| rs7531271 | -0.006945252 | 0.004448825 | 0.11849001 | 0.157249913 |
| rs75433564 | -0.006835165 | 0.004442607 | 0.123914691 | 0.157249913 |
| rs7548936 | -0.006668044 | 0.004448481 | 0.133887042 | 0.157249913 |
| rs7580304 | -0.006639032 | 0.004435304 | 0.134429515 | 0.157249913 |
| rs7583473 | -0.006565752 | 0.004434172 | 0.138682115 | 0.157249913 |
| rs7598246 | -0.006419553 | 0.00442277 | 0.146646867 | 0.157812872 |
| rs7629643 | -0.006744901 | 0.004440432 | 0.12876898 | 0.157249913 |
| rs76608582 | -0.00642033 | 0.004421135 | 0.146448672 | 0.157812872 |
| rs7675394 | -0.006643112 | 0.004444915 | 0.135034046 | 0.157249913 |
| rs76878669 | -0.006961164 | 0.004438995 | 0.116837986 | 0.157249913 |
| rs77025239 | -0.007052153 | 0.004434357 | 0.11175725 | 0.157249913 |
| rs7758776 | -0.006760233 | 0.004441902 | 0.128028357 | 0.157249913 |
| rs77675579 | -0.007134403 | 0.004429543 | 0.107258056 | 0.157249913 |
| rs7768116 | -0.006767655 | 0.004441314 | 0.127559763 | 0.157249913 |
| rs781289 | -0.007023308 | 0.004440559 | 0.113734754 | 0.157249913 |
| rs78452560 | -0.007104621 | 0.004435178 | 0.109182252 | 0.157249913 |
| rs7868164 | -0.006818685 | 0.004441122 | 0.124697366 | 0.157249913 |
| rs7868984 | -0.007999393 | 0.004412863 | 0.069871221 | 0.157249913 |
| rs7873964 | -0.006900583 | 0.004442255 | 0.120328472 | 0.157249913 |
| rs7966054 | -0.007084149 | 0.004432756 | 0.110012613 | 0.157249913 |
| rs7977614 | -0.006922583 | 0.004441579 | 0.119094375 | 0.157249913 |
| rs7987170 | -0.006961054 | 0.00443991 | 0.116919261 | 0.157249913 |
| rs7988201 | -0.007055987 | 0.0044368 | 0.111759918 | 0.157249913 |
| rs7988627 | -0.006978372 | 0.004439036 | 0.115939747 | 0.157249913 |
| rs79937071 | -0.006787815 | 0.004441664 | 0.12645927 | 0.157249913 |
| rs8008382 | -0.006583215 | 0.004433092 | 0.137539499 | 0.157249913 |
| rs8020034 | -0.006941125 | 0.004444096 | 0.118317302 | 0.157249913 |
| rs8057808 | -0.007810899 | 0.004315878 | 0.070326027 | 0.157249913 |
| rs807478 | -0.006802474 | 0.004441883 | 0.125660822 | 0.157249913 |
| rs837065 | -0.007199073 | 0.004430169 | 0.104160244 | 0.157249913 |
| rs868698 | -0.006668388 | 0.004440355 | 0.133156735 | 0.157249913 |
| rs879394 | -0.006983246 | 0.004437663 | 0.115572717 | 0.157249913 |
| rs9372625 | -0.006595444 | 0.00446219 | 0.139388214 | 0.157249913 |
| rs9643120 | -0.006971226 | 0.004441397 | 0.116507683 | 0.157249913 |
| rs9797233 | -0.006760758 | 0.004440774 | 0.127901872 | 0.157249913 |
| rs9888796 | -0.007476531 | 0.00438459 | 0.088160215 | 0.157249913 |
| All | -0.00682186 | 0.004423642 | 0.123040418 | 0.157249913 |
