# Supplementary material for: Causal roles of educational duration in bone mineral density and risk factors for osteoporosis: a Mendelian randomization study
Source: BMC Musculoskelet Disord. 2024 May 2;25:345. doi: 10.1186/s12891-024-07428-8 (PMC11064366; doi:10.1186/s12891-024-07428-8)
Supplement: Supplementary file 1 — Supplementary Material 1. [file 12891_2024_7428_MOESM1_ESM.zip › IVs of Educational attainment on milk.docx]

| SNP | b | se | P.value | adjust P.value |
| --- | --- | --- | --- | --- |
| rs10058365 | -0.002002613 | 0.002575607 | 0.436845877 | 0.528108983 |
| rs10066409 | -0.001928991 | 0.002572001 | 0.453256926 | 0.528108983 |
| rs1010334 | -0.001909264 | 0.002571208 | 0.457751072 | 0.528108983 |
| rs10189857 | -0.001967185 | 0.002575876 | 0.445048739 | 0.528108983 |
| rs10215082 | -0.002130933 | 0.002571652 | 0.407317027 | 0.528108983 |
| rs1050847 | -0.002033425 | 0.002571759 | 0.429133847 | 0.528108983 |
| rs10511592 | -0.002136712 | 0.002572153 | 0.406137655 | 0.528108983 |
| rs10518019 | -0.002135945 | 0.002574975 | 0.406820917 | 0.528108983 |
| rs10745789 | -0.002073988 | 0.002571302 | 0.419902267 | 0.528108983 |
| rs10760023 | -0.001875846 | 0.002571163 | 0.465652497 | 0.528108983 |
| rs10765775 | -0.002217047 | 0.00257558 | 0.389350743 | 0.528108983 |
| rs10844179 | -0.001853312 | 0.00257177 | 0.471132945 | 0.528108983 |
| rs10854884 | -0.001948763 | 0.002575271 | 0.449216706 | 0.528108983 |
| rs10994777 | -0.00215898 | 0.002573675 | 0.401542033 | 0.528108983 |
| rs11138947 | -0.002054408 | 0.002571532 | 0.42434576 | 0.528108983 |
| rs11155821 | -0.001944063 | 0.002573657 | 0.450026912 | 0.528108983 |
| rs11214468 | -0.001649312 | 0.002572124 | 0.52137588 | 0.536561585 |
| rs11243838 | -0.001931482 | 0.002571604 | 0.45260422 | 0.528108983 |
| rs11249939 | -0.00169544 | 0.002575099 | 0.510282307 | 0.530559568 |
| rs11572842 | -0.00210622 | 0.002571056 | 0.412669913 | 0.528108983 |
| rs115877304 | -0.0019244 | 0.002571877 | 0.45431095 | 0.528108983 |
| rs11604034 | -0.002430197 | 0.002573179 | 0.344948107 | 0.528108983 |
| rs11635966 | -0.001884169 | 0.002572411 | 0.463892323 | 0.528108983 |
| rs11661305 | -0.002021371 | 0.002572832 | 0.432066681 | 0.528108983 |
| rs11678980 | -0.002243291 | 0.002577406 | 0.384099589 | 0.528108983 |
| rs11690224 | -0.001839137 | 0.002571387 | 0.474466064 | 0.528108983 |
| rs11693764 | -0.002069556 | 0.002571409 | 0.420915858 | 0.528108983 |
| rs11714679 | -0.001889197 | 0.002571412 | 0.462526812 | 0.528108983 |
| rs11720121 | -0.001983343 | 0.002574561 | 0.441085518 | 0.528108983 |
| rs11732657 | -0.00212008 | 0.002571271 | 0.409640525 | 0.528108983 |
| rs11736863 | -0.002222072 | 0.00257386 | 0.387959926 | 0.528108983 |
| rs11764590 | -0.001944933 | 0.002573036 | 0.449714996 | 0.528108983 |
| rs117799466 | -0.002004948 | 0.002571643 | 0.435604646 | 0.528108983 |
| rs118083122 | -0.001941292 | 0.002571751 | 0.450337475 | 0.528108983 |
| rs11871429 | -0.00219829 | 0.002572084 | 0.392732241 | 0.528108983 |
| rs11915747 | -0.002092048 | 0.002576617 | 0.416828412 | 0.528108983 |
| rs12029988 | -0.002213963 | 0.002572243 | 0.389396025 | 0.528108983 |
| rs12076635 | -0.001919407 | 0.002578068 | 0.456565715 | 0.528108983 |
| rs12132451 | -0.001975455 | 0.002573634 | 0.442740158 | 0.528108983 |
| rs12468040 | -0.001993139 | 0.00257585 | 0.439061418 | 0.528108983 |
| rs12474895 | -0.002098835 | 0.002571786 | 0.414442734 | 0.528108983 |
| rs12503522 | -0.001949975 | 0.0025711 | 0.448199315 | 0.528108983 |
| rs12532494 | -0.001843461 | 0.002575261 | 0.474093578 | 0.528108983 |
| rs12574281 | -0.001980515 | 0.002571344 | 0.441165947 | 0.528108983 |
| rs12663818 | -0.002002616 | 0.002571369 | 0.4360899 | 0.528108983 |
| rs12735232 | -0.002035714 | 0.002572134 | 0.428681894 | 0.528108983 |
| rs12804787 | -0.001935025 | 0.00257107 | 0.451681579 | 0.528108983 |
| rs12921005 | -0.001914784 | 0.002571202 | 0.45645068 | 0.528108983 |
| rs12967855 | -0.001739155 | 0.002582734 | 0.500707102 | 0.528108983 |
| rs1334297 | -0.001861397 | 0.002579764 | 0.470578625 | 0.528108983 |
| rs13409451 | -0.001915721 | 0.002575835 | 0.457040914 | 0.528108983 |
| rs1363862 | -0.001891544 | 0.002571108 | 0.461917906 | 0.528108983 |
| rs1369128 | -0.002162091 | 0.002572289 | 0.400610181 | 0.528108983 |
| rs1381247 | -0.001888771 | 0.002571154 | 0.462582967 | 0.528108983 |
| rs1391438 | -0.001917875 | 0.002576366 | 0.456627928 | 0.528108983 |
| rs1452075 | -0.001985668 | 0.002571429 | 0.439993587 | 0.528108983 |
| rs145590108 | -0.002263348 | 0.002572291 | 0.378915817 | 0.528108983 |
| rs1566085 | -0.00212146 | 0.002577666 | 0.41049901 | 0.528108983 |
| rs1569266 | -0.00224329 | 0.002572026 | 0.383105897 | 0.528108983 |
| rs1620977 | -0.002203522 | 0.002578383 | 0.392764967 | 0.528108983 |
| rs1689510 | -0.002114325 | 0.002574605 | 0.411519117 | 0.528108983 |
| rs17489649 | -0.002355156 | 0.002571607 | 0.359755753 | 0.528108983 |
| rs17513684 | -0.002215209 | 0.002571658 | 0.389021352 | 0.528108983 |
| rs175325 | -0.001774164 | 0.002571962 | 0.490313925 | 0.528108983 |
| rs17563464 | -0.002228041 | 0.002573846 | 0.386684115 | 0.528108983 |
| rs17628095 | -0.002172467 | 0.002572013 | 0.398302652 | 0.528108983 |
| rs1788783 | -0.001524643 | 0.002573772 | 0.553598273 | 0.558870637 |
| rs1812587 | -0.00203583 | 0.00257163 | 0.428564957 | 0.528108983 |
| rs1835340 | -0.001899214 | 0.002571314 | 0.460140159 | 0.528108983 |
| rs185291 | -0.002058504 | 0.002582072 | 0.425317805 | 0.528108983 |
| rs1869165 | -0.001986184 | 0.002571376 | 0.439865432 | 0.528108983 |
| rs1880692 | -0.001739383 | 0.002571219 | 0.498734705 | 0.528108983 |
| rs1892417 | -0.001611426 | 0.002573431 | 0.531198168 | 0.544029041 |
| rs1917008 | -0.001997557 | 0.002571226 | 0.43722427 | 0.528108983 |
| rs192436652 | -0.0022018 | 0.002572122 | 0.391984051 | 0.528108983 |
| rs1964927 | -0.001597162 | 0.002572323 | 0.534663638 | 0.544945631 |
| rs1980251 | -0.002405228 | 0.002577515 | 0.350738615 | 0.528108983 |
| rs2145265 | -0.00177091 | 0.002571371 | 0.491010351 | 0.528108983 |
| rs215632 | -0.001783298 | 0.002571462 | 0.487998405 | 0.528108983 |
| rs2175420 | -0.002312929 | 0.002572086 | 0.368523551 | 0.528108983 |
| rs2182398 | -0.001966381 | 0.002571149 | 0.444398532 | 0.528108983 |
| rs2190872 | -0.001700737 | 0.002571317 | 0.508339 | 0.530559568 |
| rs2287838 | -0.002200997 | 0.002571316 | 0.392008457 | 0.528108983 |
| rs2299098 | -0.002005357 | 0.002574979 | 0.436105849 | 0.528108983 |
| rs2309812 | -0.002126451 | 0.002578912 | 0.409625181 | 0.528108983 |
| rs2332818 | -0.002128518 | 0.002571213 | 0.407768778 | 0.528108983 |
| rs2411453 | -0.0019064 | 0.002576341 | 0.459321918 | 0.528108983 |
| rs2559509 | -0.001767259 | 0.002572074 | 0.492022851 | 0.528108983 |
| rs2570497 | -0.001863446 | 0.002572755 | 0.468881877 | 0.528108983 |
| rs2604541 | -0.001854008 | 0.002571173 | 0.470863457 | 0.528108983 |
| rs2706762 | -0.002223757 | 0.002571961 | 0.387250104 | 0.528108983 |
| rs2725371 | -0.00238596 | 0.002573643 | 0.353887797 | 0.528108983 |
| rs2735421 | -0.002263228 | 0.002577299 | 0.379867945 | 0.528108983 |
| rs281324 | -0.001991377 | 0.002571436 | 0.438681236 | 0.528108983 |
| rs2820313 | -0.001886519 | 0.002571389 | 0.463157402 | 0.528108983 |
| rs2834011 | -0.002046461 | 0.002571762 | 0.426181642 | 0.528108983 |
| rs2974312 | -0.001914536 | 0.002573511 | 0.456913317 | 0.528108983 |
| rs2998309 | -0.001953101 | 0.002571042 | 0.447461808 | 0.528108983 |
| rs324801 | -0.002055664 | 0.002571444 | 0.424046915 | 0.528108983 |
| rs333078 | -0.001822031 | 0.002571357 | 0.478580636 | 0.528108983 |
| rs34042385 | -0.002040229 | 0.002571268 | 0.427502735 | 0.528108983 |
| rs34192341 | -0.002237672 | 0.002571768 | 0.384250741 | 0.528108983 |
| rs34364916 | -0.001842289 | 0.002571258 | 0.473686782 | 0.528108983 |
| rs34470581 | -0.00206405 | 0.002572846 | 0.422411869 | 0.528108983 |
| rs34945223 | -0.002134537 | 0.002571364 | 0.406471783 | 0.528108983 |
| rs35039375 | -0.002216102 | 0.002572588 | 0.389001718 | 0.528108983 |
| rs35091253 | -0.002146949 | 0.002574631 | 0.404345206 | 0.528108983 |
| rs35811586 | -0.001912036 | 0.002571182 | 0.45709393 | 0.528108983 |
| rs35917528 | -0.001990366 | 0.002571589 | 0.43894101 | 0.528108983 |
| rs35999162 | -0.001581653 | 0.002590475 | 0.541487718 | 0.549260269 |
| rs363096 | -0.001957894 | 0.002572683 | 0.446638045 | 0.528108983 |
| rs3747631 | -0.002170126 | 0.002577684 | 0.399849714 | 0.528108983 |
| rs3788556 | -0.001938415 | 0.002572815 | 0.451196428 | 0.528108983 |
| rs3794620 | -0.001953037 | 0.002572541 | 0.447741319 | 0.528108983 |
| rs3800925 | -0.0019419 | 0.002573983 | 0.450588649 | 0.528108983 |
| rs3825083 | -0.00203242 | 0.002572537 | 0.42950139 | 0.528108983 |
| rs3827531 | -0.001784276 | 0.002571244 | 0.487723012 | 0.528108983 |
| rs3847225 | -0.001685972 | 0.002577507 | 0.513041092 | 0.530559568 |
| rs3943093 | -0.002139724 | 0.002575267 | 0.406044619 | 0.528108983 |
| rs4130477 | -0.002008689 | 0.00257114 | 0.434658673 | 0.528108983 |
| rs4146675 | -0.001691301 | 0.002571186 | 0.510672875 | 0.530559568 |
| rs417968 | -0.002355259 | 0.002575974 | 0.360549861 | 0.528108983 |
| rs42210 | -0.001968092 | 0.002571243 | 0.44401877 | 0.528108983 |
| rs4246167 | -0.002110248 | 0.002573619 | 0.412242589 | 0.528108983 |
| rs4700393 | -0.001864145 | 0.002585355 | 0.470884702 | 0.528108983 |
| rs4726070 | -0.001854563 | 0.002572691 | 0.470992659 | 0.528108983 |
| rs4731992 | -0.00198417 | 0.002575794 | 0.441113576 | 0.528108983 |
| rs4757957 | -0.001898002 | 0.002572374 | 0.46061116 | 0.528108983 |
| rs4780563 | -0.001933922 | 0.002571572 | 0.452027649 | 0.528108983 |
| rs4808766 | -0.001855193 | 0.002571071 | 0.470562452 | 0.528108983 |
| rs4958568 | -0.001932566 | 0.002571805 | 0.452385824 | 0.528108983 |
| rs55800473 | -0.002122243 | 0.002572433 | 0.409374769 | 0.528108983 |
| rs55842281 | -0.002129975 | 0.002572217 | 0.407631138 | 0.528108983 |
| rs55859553 | -0.001858903 | 0.002571454 | 0.469741469 | 0.528108983 |
| rs55872852 | -0.002133124 | 0.002571314 | 0.406773425 | 0.528108983 |
| rs56118554 | -0.001977148 | 0.002575212 | 0.442629061 | 0.528108983 |
| rs575113 | -0.002151347 | 0.002571183 | 0.402752896 | 0.528108983 |
| rs59123361 | -0.001945246 | 0.002573509 | 0.449725352 | 0.528108983 |
| rs6071573 | -0.002025404 | 0.002573376 | 0.431246223 | 0.528108983 |
| rs613872 | -0.001892628 | 0.002573912 | 0.462149629 | 0.528108983 |
| rs61787087 | -0.001944422 | 0.002570997 | 0.449474722 | 0.528108983 |
| rs61787785 | -0.002218248 | 0.002572487 | 0.388524158 | 0.528108983 |
| rs61868084 | -0.001877395 | 0.002571747 | 0.465385505 | 0.528108983 |
| rs62018215 | -0.002218298 | 0.00257119 | 0.38827417 | 0.528108983 |
| rs62182125 | -0.002018237 | 0.002571106 | 0.432472052 | 0.528108983 |
| rs62184483 | -0.002103812 | 0.002576315 | 0.414158531 | 0.528108983 |
| rs62253608 | -0.002133819 | 0.002572095 | 0.406762943 | 0.528108983 |
| rs62389638 | -0.001814496 | 0.002573584 | 0.480781427 | 0.528108983 |
| rs6429911 | -0.001972955 | 0.002572275 | 0.443076742 | 0.528108983 |
| rs6556982 | -0.002002393 | 0.002571172 | 0.436105556 | 0.528108983 |
| rs660001 | -0.002193433 | 0.002573471 | 0.394033809 | 0.528108983 |
| rs6682095 | -0.001885899 | 0.002573053 | 0.463593728 | 0.528108983 |
| rs66844142 | -0.00201178 | 0.002571273 | 0.433975958 | 0.528108983 |
| rs6760772 | -0.002213236 | 0.002571355 | 0.389388175 | 0.528108983 |
| rs67651814 | -0.001996006 | 0.002572727 | 0.43784757 | 0.528108983 |
| rs6779254 | -0.002134688 | 0.002573408 | 0.406811375 | 0.528108983 |
| rs6789699 | -0.002219536 | 0.002572076 | 0.388172733 | 0.528108983 |
| rs67944653 | -0.002036026 | 0.002571761 | 0.428544185 | 0.528108983 |
| rs6935954 | -0.001804617 | 0.002582274 | 0.484646889 | 0.528108983 |
| rs6959579 | -0.002158249 | 0.002571324 | 0.401271186 | 0.528108983 |
| rs702606 | -0.002154804 | 0.002571463 | 0.402048595 | 0.528108983 |
| rs7031698 | -0.001999559 | 0.00257142 | 0.436799519 | 0.528108983 |
| rs7070693 | -0.001928896 | 0.002574731 | 0.453758329 | 0.528108983 |
| rs711793 | -0.001859648 | 0.002571477 | 0.46956748 | 0.528108983 |
| rs71646142 | -0.001978841 | 0.002571642 | 0.441605135 | 0.528108983 |
| rs7195278 | -0.002049028 | 0.002573796 | 0.425967359 | 0.528108983 |
| rs7233920 | -0.002032582 | 0.002573456 | 0.429629659 | 0.528108983 |
| rs72674898 | -0.002280129 | 0.002571514 | 0.375247183 | 0.528108983 |
| rs72807818 | -0.002156747 | 0.002571844 | 0.401694163 | 0.528108983 |
| rs72828517 | -0.002063936 | 0.002575676 | 0.422947317 | 0.528108983 |
| rs72977992 | -0.001911655 | 0.002571204 | 0.45718732 | 0.528108983 |
| rs73040036 | -0.001951947 | 0.002571314 | 0.447778204 | 0.528108983 |
| rs73499064 | -0.001773726 | 0.002572137 | 0.490450634 | 0.528108983 |
| rs75033012 | -0.002026615 | 0.002572274 | 0.430773296 | 0.528108983 |
| rs7526112 | -0.002064447 | 0.002574082 | 0.42254561 | 0.528108983 |
| rs7531271 | -0.001946509 | 0.002576222 | 0.449908646 | 0.528108983 |
| rs75433564 | -0.00213478 | 0.002571949 | 0.406525088 | 0.528108983 |
| rs7548936 | -0.002234144 | 0.002576338 | 0.385844517 | 0.528108983 |
| rs7580304 | -0.002144249 | 0.002571051 | 0.40428258 | 0.528108983 |
| rs7583473 | -0.001931639 | 0.002572185 | 0.452669436 | 0.528108983 |
| rs7598246 | -0.001732383 | 0.002572429 | 0.500665774 | 0.528108983 |
| rs7629643 | -0.001966506 | 0.002571207 | 0.444379766 | 0.528108983 |
| rs76608582 | -0.001986073 | 0.002572102 | 0.440020101 | 0.528108983 |
| rs7675394 | -0.001470097 | 0.00257486 | 0.568038597 | 0.570730723 |
| rs76878669 | -0.002216741 | 0.002571568 | 0.388676966 | 0.528108983 |
| rs77025239 | -0.001845566 | 0.002571688 | 0.472974486 | 0.528108983 |
| rs7758776 | -0.001943598 | 0.002571805 | 0.449809259 | 0.528108983 |
| rs77675579 | -0.0020621 | 0.002572218 | 0.422737107 | 0.528108983 |
| rs7768116 | -0.002480224 | 0.002571465 | 0.334786332 | 0.528108983 |
| rs781289 | -0.001887797 | 0.002573125 | 0.463156531 | 0.528108983 |
| rs78452560 | -0.002079049 | 0.002572821 | 0.419043694 | 0.528108983 |
| rs7868164 | -0.001919918 | 0.002571046 | 0.455216907 | 0.528108983 |
| rs7868984 | -0.002092436 | 0.002585272 | 0.418303518 | 0.528108983 |
| rs7873964 | -0.001859636 | 0.002572168 | 0.469689838 | 0.528108983 |
| rs7966054 | -0.001737811 | 0.002571903 | 0.499237153 | 0.528108983 |
| rs7977614 | -0.001814498 | 0.002572111 | 0.480529644 | 0.528108983 |
| rs7987170 | -0.001899562 | 0.002571884 | 0.460157331 | 0.528108983 |
| rs7988201 | -0.001808688 | 0.002572535 | 0.482008256 | 0.528108983 |
| rs7988627 | -0.001931789 | 0.00257186 | 0.452577231 | 0.528108983 |
| rs79937071 | -0.002042649 | 0.00257141 | 0.426980384 | 0.528108983 |
| rs8008382 | -0.001917638 | 0.002571498 | 0.455831773 | 0.528108983 |
| rs8020034 | -0.002003515 | 0.002573691 | 0.436297862 | 0.528108983 |
| rs8057808 | -0.001895289 | 0.002572463 | 0.461268023 | 0.528108983 |
| rs807478 | -0.002084 | 0.002571529 | 0.417702995 | 0.528108983 |
| rs837065 | -0.002118996 | 0.002573549 | 0.410294907 | 0.528108983 |
| rs868698 | -0.002011924 | 0.002572449 | 0.434153172 | 0.528108983 |
| rs879394 | -0.00221838 | 0.002571392 | 0.388293814 | 0.528108983 |
| rs9372625 | -0.001235247 | 0.002584478 | 0.632687106 | 0.632687106 |
| rs9643120 | -0.001907022 | 0.002572713 | 0.458542185 | 0.528108983 |
| rs9797233 | -0.001881357 | 0.002571173 | 0.464344531 | 0.528108983 |
| rs9888796 | -0.002009132 | 0.002572188 | 0.434744658 | 0.528108983 |
| All | -0.001998001 | 0.002567039 | 0.436375102 | 0.528108983 |
