# Supplementary material for: Causal roles of educational duration in bone mineral density and risk factors for osteoporosis: a Mendelian randomization study
Source: BMC Musculoskelet Disord. 2024 May 2;25:345. doi: 10.1186/s12891-024-07428-8 (PMC11064366; doi:10.1186/s12891-024-07428-8)
Supplement: Supplementary file 1 — Supplementary Material 1. [file 12891_2024_7428_MOESM1_ESM.zip › IVs of Educational attainment on physical activity.docx]

| SNP | b | se | P.value | adjust P.value |
| --- | --- | --- | --- | --- |
| rs10058365 | 0.14916244 | 0.058073416 | 0.010213529 | 0.01722063 |
| rs10066409 | 0.139140689 | 0.057751133 | 0.015982482 | 0.01727694 |
| rs1010334 | 0.146583849 | 0.058052419 | 0.011568984 | 0.01722063 |
| rs10189857 | 0.146909962 | 0.058166644 | 0.011547669 | 0.01722063 |
| rs10215082 | 0.149713011 | 0.057825885 | 0.009624642 | 0.01722063 |
| rs10511592 | 0.137974911 | 0.057604258 | 0.016610364 | 0.01727694 |
| rs10518019 | 0.144223742 | 0.05816923 | 0.013161026 | 0.01722063 |
| rs10745789 | 0.147861815 | 0.05797511 | 0.010758823 | 0.01722063 |
| rs10760023 | 0.145892861 | 0.05807157 | 0.011994909 | 0.01722063 |
| rs10765775 | 0.147582198 | 0.058141908 | 0.01113892 | 0.01722063 |
| rs10844179 | 0.144828111 | 0.058100683 | 0.01267726 | 0.01722063 |
| rs10854884 | 0.139425652 | 0.057991088 | 0.016205244 | 0.01727694 |
| rs10994777 | 0.148810057 | 0.058020751 | 0.010324332 | 0.01722063 |
| rs11138947 | 0.14617149 | 0.058075601 | 0.011838658 | 0.01722063 |
| rs11155821 | 0.145493322 | 0.058141084 | 0.012334803 | 0.01722063 |
| rs11214468 | 0.149703832 | 0.057860545 | 0.00967253 | 0.01722063 |
| rs11243838 | 0.1467794 | 0.05805451 | 0.011461534 | 0.01722063 |
| rs11249939 | 0.143597146 | 0.058167819 | 0.013561625 | 0.01722063 |
| rs11572842 | 0.143606298 | 0.058063184 | 0.013388058 | 0.01722063 |
| rs115877304 | 0.147954851 | 0.05799849 | 0.010741059 | 0.01722063 |
| rs11604034 | 0.140890414 | 0.057992663 | 0.01512165 | 0.01722063 |
| rs11635966 | 0.146031071 | 0.058099901 | 0.011955745 | 0.01722063 |
| rs11661305 | 0.139906657 | 0.057895644 | 0.015669164 | 0.01727694 |
| rs11678980 | 0.146411936 | 0.05821653 | 0.011904768 | 0.01722063 |
| rs11690224 | 0.143873359 | 0.058081039 | 0.013244957 | 0.01722063 |
| rs11693764 | 0.145279795 | 0.058089681 | 0.012385821 | 0.01722063 |
| rs11714679 | 0.144121264 | 0.058085856 | 0.01309495 | 0.01722063 |
| rs11720121 | 0.145672288 | 0.058160161 | 0.012256404 | 0.01722063 |
| rs11732657 | 0.143023202 | 0.058047044 | 0.013742725 | 0.01722063 |
| rs11736863 | 0.142926528 | 0.058119239 | 0.013924906 | 0.01722063 |
| rs11764590 | 0.143655266 | 0.058116081 | 0.013440922 | 0.01722063 |
| rs117799466 | 0.143267012 | 0.058068657 | 0.013617413 | 0.01722063 |
| rs118083122 | 0.14407506 | 0.058092135 | 0.013134075 | 0.01722063 |
| rs11871429 | 0.147483532 | 0.058033019 | 0.011041814 | 0.01722063 |
| rs11915747 | 0.140994107 | 0.058130191 | 0.015287801 | 0.01722063 |
| rs12029988 | 0.142034271 | 0.058029325 | 0.014379955 | 0.01722063 |
| rs12076635 | 0.149332821 | 0.058145602 | 0.010221148 | 0.01722063 |
| rs12132451 | 0.145025336 | 0.058142791 | 0.012620721 | 0.01722063 |
| rs12468040 | 0.148892844 | 0.058097586 | 0.010383003 | 0.01722063 |
| rs12474895 | 0.143669631 | 0.058083676 | 0.0133799 | 0.01722063 |
| rs12503522 | 0.148782081 | 0.057880174 | 0.010154628 | 0.01722063 |
| rs12532494 | 0.147190968 | 0.058143474 | 0.011357156 | 0.01722063 |
| rs12574281 | 0.142102152 | 0.057996968 | 0.014279069 | 0.01722063 |
| rs12663818 | 0.145757746 | 0.058080015 | 0.012086716 | 0.01722063 |
| rs12735232 | 0.144431025 | 0.058107256 | 0.012933557 | 0.01722063 |
| rs12804787 | 0.141321357 | 0.057917884 | 0.014686054 | 0.01722063 |
| rs12921005 | 0.146149019 | 0.058065683 | 0.011837216 | 0.01722063 |
| rs12967855 | 0.152418823 | 0.058166331 | 0.008782771 | 0.01722063 |
| rs1334297 | 0.139170522 | 0.058147444 | 0.016692696 | 0.01727694 |
| rs13409451 | 0.147669525 | 0.058147178 | 0.011098504 | 0.01722063 |
| rs1369128 | 0.142039292 | 0.058034138 | 0.014384604 | 0.01722063 |
| rs1381247 | 0.143958359 | 0.058074828 | 0.013180945 | 0.01722063 |
| rs1391438 | 0.144990261 | 0.058206173 | 0.012739223 | 0.01722063 |
| rs1452075 | 0.150024326 | 0.057766899 | 0.009402444 | 0.01722063 |
| rs145590108 | 0.14688704 | 0.058073687 | 0.011428321 | 0.01722063 |
| rs1566085 | 0.147751856 | 0.058192001 | 0.011115691 | 0.01722063 |
| rs1569266 | 0.142260837 | 0.058032901 | 0.014230865 | 0.01722063 |
| rs1620977 | 0.141346818 | 0.058194276 | 0.015145613 | 0.01722063 |
| rs1689510 | 0.152831081 | 0.057712681 | 0.008093673 | 0.01722063 |
| rs17489649 | 0.152680067 | 0.057387054 | 0.007801743 | 0.01722063 |
| rs17513684 | 0.141170389 | 0.057939727 | 0.014829997 | 0.01722063 |
| rs175325 | 0.13545488 | 0.057144724 | 0.01776967 | 0.018169313 |
| rs17563464 | 0.144182298 | 0.058143891 | 0.013147463 | 0.01722063 |
| rs17628095 | 0.148435872 | 0.057969548 | 0.010449652 | 0.01722063 |
| rs1788783 | 0.13855176 | 0.0578279 | 0.016578132 | 0.01727694 |
| rs1812587 | 0.138829527 | 0.057672606 | 0.01607534 | 0.01727694 |
| rs1835340 | 0.142695255 | 0.058049946 | 0.01396563 | 0.01722063 |
| rs185291 | 0.164218862 | 0.056994263 | 0.0039601 | 0.01722063 |
| rs1869165 | 0.147527726 | 0.058003109 | 0.010976529 | 0.01722063 |
| rs1880692 | 0.144548836 | 0.058085311 | 0.012826125 | 0.01722063 |
| rs1892417 | 0.1447519 | 0.058137495 | 0.012780779 | 0.01722063 |
| rs1917008 | 0.148055797 | 0.057954066 | 0.010627648 | 0.01722063 |
| rs192436652 | 0.141608493 | 0.057996355 | 0.014619131 | 0.01722063 |
| rs1964927 | 0.141858241 | 0.058023678 | 0.014491896 | 0.01722063 |
| rs1980251 | 0.14677565 | 0.058207273 | 0.011682139 | 0.01722063 |
| rs2145265 | 0.147768567 | 0.057985528 | 0.010822768 | 0.01722063 |
| rs215632 | 0.147413781 | 0.058013657 | 0.011053004 | 0.01722063 |
| rs2175420 | 0.142773794 | 0.058060612 | 0.013930625 | 0.01722063 |
| rs2182398 | 0.146028925 | 0.058067502 | 0.011909544 | 0.01722063 |
| rs2190872 | 0.142584574 | 0.058024052 | 0.013997182 | 0.01722063 |
| rs2287838 | 0.138925939 | 0.057653712 | 0.015967321 | 0.01727694 |
| rs2299098 | 0.150570001 | 0.05795873 | 0.009380044 | 0.01722063 |
| rs2309812 | 0.13977194 | 0.058147485 | 0.016227927 | 0.01727694 |
| rs2411453 | 0.145124988 | 0.05820491 | 0.012654541 | 0.01722063 |
| rs2559509 | 0.141698544 | 0.058001121 | 0.014564481 | 0.01722063 |
| rs2570497 | 0.144115093 | 0.058117411 | 0.01314844 | 0.01722063 |
| rs2604541 | 0.145216962 | 0.05808408 | 0.01241525 | 0.01722063 |
| rs2706762 | 0.14977794 | 0.057839382 | 0.009610162 | 0.01722063 |
| rs2725371 | 0.140652918 | 0.058000545 | 0.015307227 | 0.01722063 |
| rs2735421 | 0.146322946 | 0.058215389 | 0.011954813 | 0.01722063 |
| rs2820313 | 0.143795285 | 0.058078702 | 0.013291215 | 0.01722063 |
| rs2834011 | 0.146542554 | 0.058068064 | 0.011614871 | 0.01722063 |
| rs2974312 | 0.138392973 | 0.057794077 | 0.016639074 | 0.01727694 |
| rs2998309 | 0.146141931 | 0.058061576 | 0.011835336 | 0.01722063 |
| rs324801 | 0.14319824 | 0.05805929 | 0.013647353 | 0.01722063 |
| rs333078 | 0.146088291 | 0.058072217 | 0.011881939 | 0.01722063 |
| rs34042385 | 0.142541548 | 0.058020851 | 0.014020814 | 0.01722063 |
| rs34192341 | 0.141391849 | 0.057961944 | 0.014712037 | 0.01722063 |
| rs34364916 | 0.144952348 | 0.058089587 | 0.012584213 | 0.01722063 |
| rs34470581 | 0.144004351 | 0.058117623 | 0.013219201 | 0.01722063 |
| rs34945223 | 0.142625992 | 0.058030086 | 0.013979338 | 0.01722063 |
| rs35039375 | 0.146452668 | 0.05809662 | 0.011707287 | 0.01722063 |
| rs35091253 | 0.141206594 | 0.058075117 | 0.01503863 | 0.01722063 |
| rs35811586 | 0.143717137 | 0.058072735 | 0.013331895 | 0.01722063 |
| rs35917528 | 0.143471761 | 0.058073681 | 0.013491956 | 0.01722063 |
| rs35999162 | 0.15533527 | 0.058279488 | 0.007690809 | 0.01722063 |
| rs363096 | 0.136520995 | 0.057463413 | 0.017511432 | 0.018034161 |
| rs3747631 | 0.143079624 | 0.058218501 | 0.013985659 | 0.01722063 |
| rs3788556 | 0.147375069 | 0.058065905 | 0.011146713 | 0.01722063 |
| rs3794620 | 0.14734378 | 0.058056819 | 0.011151227 | 0.01722063 |
| rs3800925 | 0.145437633 | 0.058149201 | 0.012380421 | 0.01722063 |
| rs3825083 | 0.14510815 | 0.05811752 | 0.012531763 | 0.01722063 |
| rs3827531 | 0.144768688 | 0.05808766 | 0.01269384 | 0.01722063 |
| rs3847225 | 0.142739989 | 0.058208982 | 0.014198625 | 0.01722063 |
| rs3943093 | 0.145859922 | 0.058172747 | 0.012163733 | 0.01722063 |
| rs4130477 | 0.14346401 | 0.058060704 | 0.013476171 | 0.01722063 |
| rs4146675 | 0.141493378 | 0.057940509 | 0.014604294 | 0.01722063 |
| rs417968 | 0.163170003 | 0.05614339 | 0.003657208 | 0.01722063 |
| rs42210 | 0.143126824 | 0.05804972 | 0.01367877 | 0.01722063 |
| rs4246167 | 0.147813858 | 0.058072002 | 0.01091667 | 0.01722063 |
| rs4700393 | 0.141740797 | 0.058382911 | 0.015191604 | 0.01722063 |
| rs4726070 | 0.140789759 | 0.057965111 | 0.015145934 | 0.01722063 |
| rs4731992 | 0.154672667 | 0.057601546 | 0.007248267 | 0.01722063 |
| rs4757957 | 0.153227761 | 0.057405883 | 0.007603225 | 0.01722063 |
| rs4780563 | 0.142547977 | 0.058022273 | 0.01401884 | 0.01722063 |
| rs4808766 | 0.142192242 | 0.05799054 | 0.014206849 | 0.01722063 |
| rs4958568 | 0.134419996 | 0.056881216 | 0.018119344 | 0.018385805 |
| rs55800473 | 0.144529785 | 0.058115673 | 0.012884967 | 0.01722063 |
| rs55859553 | 0.14474153 | 0.058093061 | 0.012718852 | 0.01722063 |
| rs55872852 | 0.145325172 | 0.058088826 | 0.012357243 | 0.01722063 |
| rs56118554 | 0.150515622 | 0.057967988 | 0.009417068 | 0.01722063 |
| rs575113 | 0.148941575 | 0.057871055 | 0.01006233 | 0.01722063 |
| rs59123361 | 0.153200104 | 0.057572256 | 0.007790763 | 0.01722063 |
| rs6071573 | 0.142998728 | 0.058107784 | 0.013858047 | 0.01722063 |
| rs613872 | 0.136504677 | 0.057612122 | 0.017818215 | 0.018169313 |
| rs61787087 | 0.147407048 | 0.057993268 | 0.011028457 | 0.01722063 |
| rs61787785 | 0.146494358 | 0.058089998 | 0.011673893 | 0.01722063 |
| rs61868084 | 0.14538063 | 0.058096464 | 0.012335423 | 0.01722063 |
| rs62018215 | 0.14366354 | 0.058068987 | 0.013360412 | 0.01722063 |
| rs62182125 | 0.145474957 | 0.058080468 | 0.012255047 | 0.01722063 |
| rs62184483 | 0.139581387 | 0.058046258 | 0.016187616 | 0.01727694 |
| rs62253608 | 0.148619441 | 0.057959391 | 0.010341408 | 0.01722063 |
| rs62389638 | 0.143820937 | 0.058133767 | 0.013362258 | 0.01722063 |
| rs6429911 | 0.147001802 | 0.058061895 | 0.011347481 | 0.01722063 |
| rs6556982 | 0.148026831 | 0.057956321 | 0.010645955 | 0.01722063 |
| rs660001 | 0.145962643 | 0.058131698 | 0.01204252 | 0.01722063 |
| rs6682095 | 0.145323325 | 0.058128488 | 0.012418061 | 0.01722063 |
| rs66844142 | 0.146953413 | 0.058031187 | 0.01133112 | 0.01722063 |
| rs6760772 | 0.144136563 | 0.058083851 | 0.013082131 | 0.01722063 |
| rs67651814 | 0.146182079 | 0.058104987 | 0.011875355 | 0.01722063 |
| rs6779254 | 0.146176692 | 0.05812364 | 0.011905714 | 0.01722063 |
| rs6789699 | 0.149940562 | 0.057831531 | 0.00952222 | 0.01722063 |
| rs67944653 | 0.141691866 | 0.05798904 | 0.014548596 | 0.01722063 |
| rs6935954 | 0.133765694 | 0.057910121 | 0.020894546 | 0.020894546 |
| rs6959579 | 0.145515204 | 0.058084424 | 0.012236963 | 0.01722063 |
| rs702606 | 0.141902137 | 0.057987487 | 0.014400428 | 0.01722063 |
| rs7031698 | 0.14488525 | 0.05809222 | 0.012629263 | 0.01722063 |
| rs7070693 | 0.140078417 | 0.058002501 | 0.01573344 | 0.01727694 |
| rs711793 | 0.142706775 | 0.058039437 | 0.013940622 | 0.01722063 |
| rs71646142 | 0.148658497 | 0.057928493 | 0.010280725 | 0.01722063 |
| rs7195278 | 0.142355663 | 0.058097945 | 0.014274904 | 0.01722063 |
| rs7233920 | 0.147097909 | 0.058093779 | 0.011338916 | 0.01722063 |
| rs72674898 | 0.139241392 | 0.057715585 | 0.015841623 | 0.01727694 |
| rs72807818 | 0.15240277 | 0.057455539 | 0.007989009 | 0.01722063 |
| rs72828517 | 0.144273372 | 0.058187891 | 0.013158895 | 0.01722063 |
| rs72977992 | 0.144452528 | 0.058083672 | 0.012883515 | 0.01722063 |
| rs73040036 | 0.146359246 | 0.058060811 | 0.011709095 | 0.01722063 |
| rs73499064 | 0.146028354 | 0.058093642 | 0.011948154 | 0.01722063 |
| rs75033012 | 0.150156596 | 0.05782046 | 0.009405678 | 0.01722063 |
| rs7526112 | 0.138527173 | 0.057856813 | 0.016651647 | 0.01727694 |
| rs7531271 | 0.141646031 | 0.058143145 | 0.014844097 | 0.01722063 |
| rs75433564 | 0.14659056 | 0.058070962 | 0.011591741 | 0.01722063 |
| rs7548936 | 0.134859026 | 0.057627739 | 0.019274678 | 0.019368244 |
| rs7580304 | 0.146754857 | 0.058036586 | 0.011449853 | 0.01722063 |
| rs7583473 | 0.141896708 | 0.058016871 | 0.014453776 | 0.01722063 |
| rs7598246 | 0.140810146 | 0.057953281 | 0.015110562 | 0.01722063 |
| rs7629643 | 0.140292492 | 0.05778364 | 0.015186825 | 0.01722063 |
| rs76608582 | 0.141846423 | 0.058013723 | 0.014483228 | 0.01722063 |
| rs7675394 | 0.152462082 | 0.057775449 | 0.008318209 | 0.01722063 |
| rs76878669 | 0.138177061 | 0.05756148 | 0.016372125 | 0.01727694 |
| rs77025239 | 0.144747903 | 0.058097252 | 0.012721359 | 0.01722063 |
| rs7758776 | 0.141264169 | 0.057957389 | 0.014794131 | 0.01722063 |
| rs77675579 | 0.141206404 | 0.057972014 | 0.014860193 | 0.01722063 |
| rs7768116 | 0.138879069 | 0.057652381 | 0.016000493 | 0.01727694 |
| rs781289 | 0.144260404 | 0.058128292 | 0.013073571 | 0.01722063 |
| rs78452560 | 0.144415325 | 0.058125002 | 0.012970995 | 0.01722063 |
| rs7868164 | 0.147205394 | 0.05801179 | 0.011164475 | 0.01722063 |
| rs7868984 | 0.154738229 | 0.058128659 | 0.007767892 | 0.01722063 |
| rs7873964 | 0.14740333 | 0.05804091 | 0.011096491 | 0.01722063 |
| rs7966054 | 0.142382154 | 0.058036155 | 0.014153829 | 0.01722063 |
| rs7977614 | 0.145379106 | 0.058105638 | 0.012350113 | 0.01722063 |
| rs7987170 | 0.146126669 | 0.058087329 | 0.01188173 | 0.01722063 |
| rs7988201 | 0.14334837 | 0.058096026 | 0.01360836 | 0.01722063 |
| rs7988627 | 0.143219631 | 0.058073721 | 0.013656675 | 0.01722063 |
| rs79937071 | 0.14283271 | 0.05804122 | 0.013859517 | 0.01722063 |
| rs8008382 | 0.144186902 | 0.058088595 | 0.01305778 | 0.01722063 |
| rs8020034 | 0.152377478 | 0.057694926 | 0.008263982 | 0.01722063 |
| rs8057808 | 0.145125117 | 0.058115998 | 0.012519141 | 0.01722063 |
| rs807478 | 0.14591412 | 0.058080367 | 0.011995402 | 0.01722063 |
| rs837065 | 0.149896533 | 0.057930775 | 0.009667203 | 0.01722063 |
| rs868698 | 0.143762609 | 0.058102973 | 0.013350769 | 0.01722063 |
| rs879394 | 0.140552905 | 0.057864603 | 0.015140689 | 0.01722063 |
| rs9372625 | 0.13684059 | 0.05819618 | 0.018704565 | 0.018887048 |
| rs9643120 | 0.1523894 | 0.057583634 | 0.008135326 | 0.01722063 |
| rs9797233 | 0.141571477 | 0.05794706 | 0.014560995 | 0.01722063 |
| rs9888796 | 0.145934829 | 0.058097557 | 0.01200856 | 0.01722063 |
| All | 0.144853841 | 0.0578498 | 0.012281038 | 0.01722063 |
