# Supplementary material for: Causal roles of educational duration in bone mineral density and risk factors for osteoporosis: a Mendelian randomization study
Source: BMC Musculoskelet Disord. 2024 May 2;25:345. doi: 10.1186/s12891-024-07428-8 (PMC11064366; doi:10.1186/s12891-024-07428-8)
Supplement: Supplementary file 1 — Supplementary Material 1. [file 12891_2024_7428_MOESM1_ESM.zip › IVs of Educational attainment on salt intake.docx]

| SNP | b | se | P.value | adjust P.value |
| --- | --- | --- | --- | --- |
| rs10058365 | -0.035118394 | 0.0033889 | 3.66E-25 | 4.74E-25 |
| rs10066409 | -0.035283325 | 0.003381887 | 1.75E-25 | 4.74E-25 |
| rs1010334 | -0.035294671 | 0.003379988 | 1.59E-25 | 4.74E-25 |
| rs10189857 | -0.035113322 | 0.003389272 | 3.76E-25 | 4.74E-25 |
| rs10215082 | -0.035081746 | 0.003382731 | 3.37E-25 | 4.74E-25 |
| rs1050847 | -0.034943821 | 0.003374697 | 3.99E-25 | 4.74E-25 |
| rs10511592 | -0.035103037 | 0.003383995 | 3.28E-25 | 4.74E-25 |
| rs10518019 | -0.035922633 | 0.003321595 | 2.93E-27 | 3.10E-25 |
| rs10745789 | -0.035524694 | 0.003355777 | 3.46E-26 | 4.74E-25 |
| rs10760023 | -0.035015035 | 0.003378425 | 3.60E-25 | 4.74E-25 |
| rs10765775 | -0.034954348 | 0.003384474 | 5.27E-25 | 5.59E-25 |
| rs10844179 | -0.035246297 | 0.003382909 | 2.03E-25 | 4.74E-25 |
| rs10854884 | -0.03560521 | 0.003367037 | 3.91E-26 | 4.74E-25 |
| rs10994777 | -0.034723587 | 0.003359905 | 4.91E-25 | 5.35E-25 |
| rs11138947 | -0.035104297 | 0.003383151 | 3.18E-25 | 4.74E-25 |
| rs11155821 | -0.035271918 | 0.00338512 | 2.02E-25 | 4.74E-25 |
| rs11214468 | -0.035350164 | 0.003378463 | 1.27E-25 | 4.74E-25 |
| rs11243838 | -0.035115141 | 0.003383472 | 3.11E-25 | 4.74E-25 |
| rs11249939 | -0.03493238 | 0.003382481 | 5.30E-25 | 5.59E-25 |
| rs11572842 | -0.035184294 | 0.003383158 | 2.48E-25 | 4.74E-25 |
| rs115877304 | -0.035419984 | 0.003371856 | 8.23E-26 | 4.74E-25 |
| rs11604034 | -0.035057792 | 0.003384244 | 3.80E-25 | 4.74E-25 |
| rs11635966 | -0.035349247 | 0.003379107 | 1.30E-25 | 4.74E-25 |
| rs11661305 | -0.035164499 | 0.003385488 | 2.85E-25 | 4.74E-25 |
| rs11678980 | -0.034965651 | 0.003388198 | 5.73E-25 | 6.02E-25 |
| rs11690224 | -0.035013599 | 0.00337898 | 3.68E-25 | 4.74E-25 |
| rs11693764 | -0.035351362 | 0.003376453 | 1.19E-25 | 4.74E-25 |
| rs11714679 | -0.035208779 | 0.003383354 | 2.32E-25 | 4.74E-25 |
| rs11720121 | -0.035007404 | 0.003384882 | 4.54E-25 | 5.01E-25 |
| rs11732657 | -0.035118926 | 0.00338307 | 3.03E-25 | 4.74E-25 |
| rs11736863 | -0.035472324 | 0.003374301 | 7.57E-26 | 4.74E-25 |
| rs11764590 | -0.034595753 | 0.003335604 | 3.34E-25 | 4.74E-25 |
| rs117799466 | -0.035140577 | 0.003383882 | 2.91E-25 | 4.74E-25 |
| rs118083122 | -0.035347201 | 0.003377448 | 1.24E-25 | 4.74E-25 |
| rs11871429 | -0.034965682 | 0.003377414 | 4.06E-25 | 4.74E-25 |
| rs11915747 | -0.03416465 | 0.003294319 | 3.37E-25 | 4.74E-25 |
| rs12029988 | -0.035160327 | 0.003384807 | 2.82E-25 | 4.74E-25 |
| rs12076635 | -0.035211646 | 0.003392323 | 3.06E-25 | 4.74E-25 |
| rs12132451 | -0.035131049 | 0.003386444 | 3.26E-25 | 4.74E-25 |
| rs12468040 | -0.035444959 | 0.00338144 | 1.04E-25 | 4.74E-25 |
| rs12474895 | -0.035271473 | 0.003381939 | 1.82E-25 | 4.74E-25 |
| rs12503522 | -0.035126548 | 0.003382977 | 2.95E-25 | 4.74E-25 |
| rs12532494 | -0.034980463 | 0.003384987 | 4.95E-25 | 5.35E-25 |
| rs12574281 | -0.035238473 | 0.003382448 | 2.05E-25 | 4.74E-25 |
| rs12663818 | -0.035125407 | 0.003383348 | 3.00E-25 | 4.74E-25 |
| rs12735232 | -0.035214886 | 0.00338423 | 2.34E-25 | 4.74E-25 |
| rs12804787 | -0.035465517 | 0.003362879 | 5.29E-26 | 4.74E-25 |
| rs12921005 | -0.035211695 | 0.003382917 | 2.26E-25 | 4.74E-25 |
| rs12967855 | -0.03441286 | 0.003365822 | 1.54E-24 | 1.55E-24 |
| rs1334297 | -0.034918299 | 0.003390454 | 7.12E-25 | 7.36E-25 |
| rs13409451 | -0.035391274 | 0.003384272 | 1.35E-25 | 4.74E-25 |
| rs1363862 | -0.034901871 | 0.003368043 | 3.67E-25 | 4.74E-25 |
| rs1369128 | -0.035296835 | 0.003382017 | 1.69E-25 | 4.74E-25 |
| rs1381247 | -0.034972837 | 0.003375208 | 3.70E-25 | 4.74E-25 |
| rs1391438 | -0.035342347 | 0.003387015 | 1.72E-25 | 4.74E-25 |
| rs1452075 | -0.035392407 | 0.003372939 | 9.30E-26 | 4.74E-25 |
| rs145590108 | -0.035180013 | 0.00338491 | 2.66E-25 | 4.74E-25 |
| rs1566085 | -0.035676402 | 0.003369181 | 3.35E-26 | 4.74E-25 |
| rs1569266 | -0.035393333 | 0.003374811 | 9.86E-26 | 4.74E-25 |
| rs1620977 | -0.034784582 | 0.00338109 | 7.98E-25 | 8.18E-25 |
| rs1689510 | -0.035244858 | 0.003387115 | 2.34E-25 | 4.74E-25 |
| rs17489649 | -0.03508121 | 0.003382629 | 3.36E-25 | 4.74E-25 |
| rs17513684 | -0.03526324 | 0.003381991 | 1.87E-25 | 4.74E-25 |
| rs175325 | -0.035307986 | 0.003380625 | 1.56E-25 | 4.74E-25 |
| rs17563464 | -0.035197998 | 0.003386737 | 2.67E-25 | 4.74E-25 |
| rs17628095 | -0.034935685 | 0.003375008 | 4.13E-25 | 4.76E-25 |
| rs1788783 | -0.035251756 | 0.003385748 | 2.19E-25 | 4.74E-25 |
| rs1812587 | -0.035175835 | 0.003383946 | 2.62E-25 | 4.74E-25 |
| rs1835340 | -0.03517302 | 0.003383566 | 2.61E-25 | 4.74E-25 |
| rs185291 | -0.034776411 | 0.003388615 | 1.04E-24 | 1.06E-24 |
| rs1869165 | -0.035274631 | 0.003381161 | 1.76E-25 | 4.74E-25 |
| rs1880692 | -0.035030688 | 0.003379534 | 3.56E-25 | 4.74E-25 |
| rs1892417 | -0.03484004 | 0.003371405 | 4.95E-25 | 5.35E-25 |
| rs1917008 | -0.035309553 | 0.003378808 | 1.46E-25 | 4.74E-25 |
| rs192436652 | -0.035003539 | 0.003380034 | 3.93E-25 | 4.74E-25 |
| rs1964927 | -0.034888747 | 0.0033717 | 4.29E-25 | 4.87E-25 |
| rs1980251 | -0.035635444 | 0.00337201 | 4.19E-26 | 4.74E-25 |
| rs2145265 | -0.035045701 | 0.003380681 | 3.52E-25 | 4.74E-25 |
| rs215632 | -0.034889985 | 0.003368318 | 3.84E-25 | 4.74E-25 |
| rs2175420 | -0.035138709 | 0.003384401 | 2.98E-25 | 4.74E-25 |
| rs2182398 | -0.035111995 | 0.00338274 | 3.07E-25 | 4.74E-25 |
| rs2190872 | -0.035239467 | 0.00338238 | 2.04E-25 | 4.74E-25 |
| rs2287838 | -0.034836602 | 0.00336055 | 3.53E-25 | 4.74E-25 |
| rs2299098 | -0.035727707 | 0.003352032 | 1.59E-26 | 4.74E-25 |
| rs2309812 | -0.035401436 | 0.003389193 | 1.54E-25 | 4.74E-25 |
| rs2332818 | -0.034786051 | 0.00335205 | 3.14E-25 | 4.74E-25 |
| rs2411453 | -0.036154107 | 0.003293282 | 4.87E-28 | 1.03E-25 |
| rs2559509 | -0.035131653 | 0.003384354 | 3.04E-25 | 4.74E-25 |
| rs2570497 | -0.0348336 | 0.003367837 | 4.50E-25 | 5.01E-25 |
| rs2604541 | -0.035050289 | 0.003380515 | 3.46E-25 | 4.74E-25 |
| rs2706762 | -0.035647737 | 0.00334085 | 1.40E-26 | 4.74E-25 |
| rs2725371 | -0.035169173 | 0.003386666 | 2.91E-25 | 4.74E-25 |
| rs2735421 | -0.035078362 | 0.003390777 | 4.40E-25 | 4.93E-25 |
| rs281324 | -0.035241413 | 0.003382498 | 2.04E-25 | 4.74E-25 |
| rs2820313 | -0.034986863 | 0.003377298 | 3.79E-25 | 4.74E-25 |
| rs2834011 | -0.035313643 | 0.003379806 | 1.49E-25 | 4.74E-25 |
| rs2974312 | -0.035108634 | 0.003386039 | 3.44E-25 | 4.74E-25 |
| rs2998309 | -0.03510632 | 0.003382447 | 3.09E-25 | 4.74E-25 |
| rs324801 | -0.035221757 | 0.003383019 | 2.20E-25 | 4.74E-25 |
| rs333078 | -0.035006579 | 0.003378419 | 3.70E-25 | 4.74E-25 |
| rs34042385 | -0.035269552 | 0.003381121 | 1.78E-25 | 4.74E-25 |
| rs34192341 | -0.035248951 | 0.00338267 | 2.00E-25 | 4.74E-25 |
| rs34364916 | -0.035336564 | 0.003377248 | 1.28E-25 | 4.74E-25 |
| rs34470581 | -0.035355305 | 0.003379804 | 1.31E-25 | 4.74E-25 |
| rs34945223 | -0.035241126 | 0.003382447 | 2.03E-25 | 4.74E-25 |
| rs35039375 | -0.035293398 | 0.003382736 | 1.75E-25 | 4.74E-25 |
| rs35091253 | -0.035338257 | 0.003384282 | 1.60E-25 | 4.74E-25 |
| rs35811586 | -0.035565484 | 0.003348415 | 2.37E-26 | 4.74E-25 |
| rs35917528 | -0.035089367 | 0.003382858 | 3.30E-25 | 4.74E-25 |
| rs35999162 | -0.036215599 | 0.003364993 | 5.18E-27 | 3.66E-25 |
| rs363096 | -0.035437649 | 0.003373375 | 8.18E-26 | 4.74E-25 |
| rs3747631 | -0.034812935 | 0.003381119 | 7.32E-25 | 7.54E-25 |
| rs3788556 | -0.03527863 | 0.003383468 | 1.87E-25 | 4.74E-25 |
| rs3794620 | -0.035056596 | 0.003383257 | 3.70E-25 | 4.74E-25 |
| rs3800925 | -0.035186072 | 0.00338713 | 2.81E-25 | 4.74E-25 |
| rs3825083 | -0.035161799 | 0.003385174 | 2.84E-25 | 4.74E-25 |
| rs3827531 | -0.035298367 | 0.003379532 | 1.55E-25 | 4.74E-25 |
| rs3847225 | -0.035579392 | 0.003376673 | 5.84E-26 | 4.74E-25 |
| rs3943093 | -0.035168883 | 0.003388809 | 3.12E-25 | 4.74E-25 |
| rs4130477 | -0.035009762 | 0.003378092 | 3.62E-25 | 4.74E-25 |
| rs4146675 | -0.035184192 | 0.003383329 | 2.50E-25 | 4.74E-25 |
| rs417968 | -0.035352448 | 0.003386081 | 1.62E-25 | 4.74E-25 |
| rs42210 | -0.035107066 | 0.003382715 | 3.11E-25 | 4.74E-25 |
| rs4246167 | -0.034869467 | 0.003374613 | 5.00E-25 | 5.38E-25 |
| rs4700393 | -0.034615059 | 0.003387 | 1.61E-24 | 1.61E-24 |
| rs4726070 | -0.035417405 | 0.003375058 | 9.22E-26 | 4.74E-25 |
| rs4731992 | -0.035398793 | 0.003383877 | 1.30E-25 | 4.74E-25 |
| rs4757957 | -0.03492843 | 0.003375484 | 4.29E-25 | 4.87E-25 |
| rs4780563 | -0.03517748 | 0.003383869 | 2.60E-25 | 4.74E-25 |
| rs4808766 | -0.035140225 | 0.003383173 | 2.85E-25 | 4.74E-25 |
| rs4958568 | -0.034993273 | 0.003378774 | 3.90E-25 | 4.74E-25 |
| rs55800473 | -0.035279831 | 0.003382836 | 1.83E-25 | 4.74E-25 |
| rs55842281 | -0.03514138 | 0.003384636 | 2.98E-25 | 4.74E-25 |
| rs55859553 | -0.035046233 | 0.003380869 | 3.54E-25 | 4.74E-25 |
| rs55872852 | -0.035136535 | 0.003383467 | 2.91E-25 | 4.74E-25 |
| rs56118554 | -0.035292171 | 0.00338681 | 2.00E-25 | 4.74E-25 |
| rs575113 | -0.035079143 | 0.003381797 | 3.29E-25 | 4.74E-25 |
| rs59123361 | -0.035274467 | 0.003384959 | 1.99E-25 | 4.74E-25 |
| rs6071573 | -0.035037587 | 0.003383968 | 4.01E-25 | 4.74E-25 |
| rs613872 | -0.035262663 | 0.003385644 | 2.11E-25 | 4.74E-25 |
| rs61787087 | -0.035156946 | 0.00338315 | 2.70E-25 | 4.74E-25 |
| rs61787785 | -0.035005867 | 0.003381025 | 4.03E-25 | 4.74E-25 |
| rs61868084 | -0.034760796 | 0.003352589 | 3.45E-25 | 4.74E-25 |
| rs62018215 | -0.035062089 | 0.003381082 | 3.39E-25 | 4.74E-25 |
| rs62182125 | -0.035226466 | 0.003382464 | 2.13E-25 | 4.74E-25 |
| rs62184483 | -0.034534294 | 0.003351097 | 6.66E-25 | 6.96E-25 |
| rs62253608 | -0.035258874 | 0.003382991 | 1.96E-25 | 4.74E-25 |
| rs62389638 | -0.03518232 | 0.003386436 | 2.78E-25 | 4.74E-25 |
| rs6429911 | -0.035250607 | 0.003383585 | 2.05E-25 | 4.74E-25 |
| rs6556982 | -0.035095593 | 0.003382362 | 3.19E-25 | 4.74E-25 |
| rs660001 | -0.035163518 | 0.003386511 | 2.95E-25 | 4.74E-25 |
| rs6682095 | -0.035262979 | 0.003384333 | 2.02E-25 | 4.74E-25 |
| rs66844142 | -0.035049613 | 0.003380537 | 3.46E-25 | 4.74E-25 |
| rs6760772 | -0.034948946 | 0.003373851 | 3.82E-25 | 4.74E-25 |
| rs67651814 | -0.035244973 | 0.003384386 | 2.14E-25 | 4.74E-25 |
| rs6779254 | -0.035596404 | 0.003359576 | 3.13E-26 | 4.74E-25 |
| rs6789699 | -0.03517349 | 0.003384587 | 2.69E-25 | 4.74E-25 |
| rs67944653 | -0.035059807 | 0.003382073 | 3.53E-25 | 4.74E-25 |
| rs6935954 | -0.034666338 | 0.003383354 | 1.23E-24 | 1.24E-24 |
| rs6959579 | -0.035023599 | 0.003379371 | 3.62E-25 | 4.74E-25 |
| rs702606 | -0.035041208 | 0.003380699 | 3.57E-25 | 4.74E-25 |
| rs7031698 | -0.035273938 | 0.003381253 | 1.77E-25 | 4.74E-25 |
| rs7070693 | -0.035010952 | 0.003385125 | 4.52E-25 | 5.01E-25 |
| rs711793 | -0.03518473 | 0.003383716 | 2.53E-25 | 4.74E-25 |
| rs71646142 | -0.035244613 | 0.003382688 | 2.03E-25 | 4.74E-25 |
| rs7195278 | -0.035217468 | 0.003386455 | 2.49E-25 | 4.74E-25 |
| rs7233920 | -0.034989396 | 0.003382034 | 4.38E-25 | 4.93E-25 |
| rs72674898 | -0.035173988 | 0.003383773 | 2.62E-25 | 4.74E-25 |
| rs72807818 | -0.035011045 | 0.003379686 | 3.80E-25 | 4.74E-25 |
| rs72828517 | -0.034954115 | 0.003384512 | 5.28E-25 | 5.59E-25 |
| rs72977992 | -0.035253203 | 0.003381686 | 1.91E-25 | 4.74E-25 |
| rs73040036 | -0.035379293 | 0.003373769 | 9.96E-26 | 4.74E-25 |
| rs73499064 | -0.035392208 | 0.003375355 | 1.01E-25 | 4.74E-25 |
| rs75033012 | -0.035492273 | 0.003366441 | 5.47E-26 | 4.74E-25 |
| rs7526112 | -0.034677624 | 0.003356784 | 5.12E-25 | 5.49E-25 |
| rs7531271 | -0.035343986 | 0.003386762 | 1.70E-25 | 4.74E-25 |
| rs75433564 | -0.03505729 | 0.003382264 | 3.58E-25 | 4.74E-25 |
| rs7548936 | -0.0345525 | 0.003353297 | 6.76E-25 | 7.02E-25 |
| rs7580304 | -0.035176199 | 0.003383185 | 2.55E-25 | 4.74E-25 |
| rs7583473 | -0.035120356 | 0.003384356 | 3.15E-25 | 4.74E-25 |
| rs7598246 | -0.034808686 | 0.003363776 | 4.27E-25 | 4.87E-25 |
| rs7629643 | -0.035162576 | 0.003383464 | 2.68E-25 | 4.74E-25 |
| rs76608582 | -0.035201326 | 0.00338425 | 2.44E-25 | 4.74E-25 |
| rs7675394 | -0.035215699 | 0.003387953 | 2.63E-25 | 4.74E-25 |
| rs76878669 | -0.035205938 | 0.003383511 | 2.35E-25 | 4.74E-25 |
| rs77025239 | -0.035136001 | 0.003383928 | 2.96E-25 | 4.74E-25 |
| rs7758776 | -0.034917492 | 0.003372589 | 4.04E-25 | 4.74E-25 |
| rs77675579 | -0.035197366 | 0.003384506 | 2.49E-25 | 4.74E-25 |
| rs7768116 | -0.035361977 | 0.003375677 | 1.12E-25 | 4.74E-25 |
| rs781289 | -0.035027643 | 0.003383458 | 4.07E-25 | 4.74E-25 |
| rs78452560 | -0.03528234 | 0.003383581 | 1.86E-25 | 4.74E-25 |
| rs7868164 | -0.035342509 | 0.003375993 | 1.20E-25 | 4.74E-25 |
| rs7868984 | -0.035823654 | 0.003380172 | 3.04E-26 | 4.74E-25 |
| rs7873964 | -0.035336738 | 0.003379416 | 1.37E-25 | 4.74E-25 |
| rs7966054 | -0.035377383 | 0.003375803 | 1.07E-25 | 4.74E-25 |
| rs7977614 | -0.034845058 | 0.003366231 | 4.13E-25 | 4.76E-25 |
| rs7987170 | -0.0355825 | 0.003351582 | 2.49E-26 | 4.74E-25 |
| rs7988201 | -0.035241426 | 0.003384193 | 2.15E-25 | 4.74E-25 |
| rs7988627 | -0.034937554 | 0.00337459 | 4.05E-25 | 4.74E-25 |
| rs79937071 | -0.035113562 | 0.003383207 | 3.10E-25 | 4.74E-25 |
| rs8008382 | -0.034933582 | 0.003372992 | 3.90E-25 | 4.74E-25 |
| rs8020034 | -0.034718764 | 0.003359183 | 4.87E-25 | 5.35E-25 |
| rs8057808 | -0.035021333 | 0.003381574 | 3.91E-25 | 4.74E-25 |
| rs807478 | -0.035148187 | 0.00338378 | 2.83E-25 | 4.74E-25 |
| rs837065 | -0.035339003 | 0.003382174 | 1.49E-25 | 4.74E-25 |
| rs868698 | -0.035180332 | 0.003384936 | 2.66E-25 | 4.74E-25 |
| rs879394 | -0.035329674 | 0.003377988 | 1.34E-25 | 4.74E-25 |
| rs9372625 | -0.034792856 | 0.003393612 | 1.15E-24 | 1.17E-24 |
| rs9643120 | -0.035594929 | 0.003355623 | 2.75E-26 | 4.74E-25 |
| rs9797233 | -0.035334963 | 0.003376996 | 1.27E-25 | 4.74E-25 |
| rs9888796 | -0.035104546 | 0.003384067 | 3.27E-25 | 4.74E-25 |
| All | -0.035164254 | 0.003369895 | 1.72E-25 | 4.74E-25 |
