# Supplementary material for: Causal roles of educational duration in bone mineral density and risk factors for osteoporosis: a Mendelian randomization study
Source: BMC Musculoskelet Disord. 2024 May 2;25:345. doi: 10.1186/s12891-024-07428-8 (PMC11064366; doi:10.1186/s12891-024-07428-8)
Supplement: Supplementary file 1 — Supplementary Material 1. [file 12891_2024_7428_MOESM1_ESM.zip › IVs of Educational attainment on smoking.docx]

| SNP | b | se | P.value | adjust P.value |
| --- | --- | --- | --- | --- |
| rs10058365 | -0.030540455 | 0.002957663 | 5.38E-25 | 8.33E-25 |
| rs10066409 | -0.030429856 | 0.002951884 | 6.44E-25 | 8.33E-25 |
| rs1010334 | -0.030623183 | 0.002950379 | 3.08E-25 | 8.33E-25 |
| rs10189857 | -0.03089813 | 0.002941354 | 8.22E-26 | 8.33E-25 |
| rs10215082 | -0.030483527 | 0.002952955 | 5.54E-25 | 8.33E-25 |
| rs1050847 | -0.030169065 | 0.002925574 | 6.21E-25 | 8.33E-25 |
| rs10511592 | -0.030480041 | 0.002953436 | 5.71E-25 | 8.33E-25 |
| rs10518019 | -0.030741436 | 0.002950694 | 2.04E-25 | 8.33E-25 |
| rs10745789 | -0.030476382 | 0.00295234 | 5.56E-25 | 8.33E-25 |
| rs10760023 | -0.030570146 | 0.002952048 | 3.95E-25 | 8.33E-25 |
| rs10765775 | -0.030341181 | 0.002953804 | 9.43E-25 | 9.85E-25 |
| rs10844179 | -0.030608454 | 0.002951749 | 3.41E-25 | 8.33E-25 |
| rs10854884 | -0.030680348 | 0.002954127 | 2.88E-25 | 8.33E-25 |
| rs10994777 | -0.030363842 | 0.002951685 | 8.07E-25 | 8.96E-25 |
| rs11138947 | -0.03052558 | 0.002953109 | 4.80E-25 | 8.33E-25 |
| rs11155821 | -0.03053162 | 0.002955561 | 5.14E-25 | 8.33E-25 |
| rs11214468 | -0.029671885 | 0.002802861 | 3.45E-26 | 8.33E-25 |
| rs11243838 | -0.03055921 | 0.00295286 | 4.23E-25 | 8.33E-25 |
| rs11249939 | -0.030403645 | 0.002949834 | 6.56E-25 | 8.33E-25 |
| rs11572842 | -0.030656264 | 0.002947863 | 2.49E-25 | 8.33E-25 |
| rs115877304 | -0.030477611 | 0.002953055 | 5.68E-25 | 8.33E-25 |
| rs11604034 | -0.030219621 | 0.002939323 | 8.57E-25 | 9.33E-25 |
| rs11635966 | -0.030579663 | 0.002953401 | 4.01E-25 | 8.33E-25 |
| rs11661305 | -0.030452215 | 0.002953644 | 6.35E-25 | 8.33E-25 |
| rs11678980 | -0.030009085 | 0.002933641 | 1.47E-24 | 1.47E-24 |
| rs11690224 | -0.030511757 | 0.002952944 | 5.02E-25 | 8.33E-25 |
| rs11693764 | -0.030626581 | 0.002950365 | 3.04E-25 | 8.33E-25 |
| rs11714679 | -0.030518488 | 0.002953008 | 4.91E-25 | 8.33E-25 |
| rs11720121 | -0.030318942 | 0.002950964 | 9.21E-25 | 9.72E-25 |
| rs11732657 | -0.030529049 | 0.002952778 | 4.69E-25 | 8.33E-25 |
| rs11736863 | -0.030633342 | 0.002953909 | 3.38E-25 | 8.33E-25 |
| rs11764590 | -0.030587848 | 0.002953947 | 3.98E-25 | 8.33E-25 |
| rs117799466 | -0.030588554 | 0.002952196 | 3.72E-25 | 8.33E-25 |
| rs118083122 | -0.03062609 | 0.002950818 | 3.09E-25 | 8.33E-25 |
| rs11871429 | -0.030699926 | 0.00294712 | 2.08E-25 | 8.33E-25 |
| rs11915747 | -0.029747771 | 0.002893602 | 8.63E-25 | 9.33E-25 |
| rs12029988 | -0.030486318 | 0.002953702 | 5.64E-25 | 8.33E-25 |
| rs12076635 | -0.030169432 | 0.002949002 | 1.45E-24 | 1.46E-24 |
| rs12132451 | -0.030695039 | 0.002950759 | 2.42E-25 | 8.33E-25 |
| rs12468040 | -0.030582521 | 0.002957688 | 4.64E-25 | 8.33E-25 |
| rs12474895 | -0.030743802 | 0.002942512 | 1.49E-25 | 8.33E-25 |
| rs12503522 | -0.030237364 | 0.00293195 | 6.15E-25 | 8.33E-25 |
| rs12532494 | -0.03097538 | 0.002931198 | 4.22E-26 | 8.33E-25 |
| rs12574281 | -0.030600015 | 0.002951396 | 3.47E-25 | 8.33E-25 |
| rs12663818 | -0.030611057 | 0.002951005 | 3.29E-25 | 8.33E-25 |
| rs12735232 | -0.030722115 | 0.002945684 | 1.82E-25 | 8.33E-25 |
| rs12804787 | -0.030390326 | 0.00294822 | 6.48E-25 | 8.33E-25 |
| rs12921005 | -0.030791923 | 0.002934372 | 9.25E-26 | 8.33E-25 |
| rs12967855 | -0.030480365 | 0.002965951 | 8.97E-25 | 9.56E-25 |
| rs1334297 | -0.030429364 | 0.002961966 | 9.29E-25 | 9.75E-25 |
| rs13409451 | -0.030417362 | 0.002956871 | 8.06E-25 | 8.96E-25 |
| rs1363862 | -0.030634335 | 0.00294941 | 2.85E-25 | 8.33E-25 |
| rs1369128 | -0.030502851 | 0.002954037 | 5.39E-25 | 8.33E-25 |
| rs1381247 | -0.030591644 | 0.002951396 | 3.57E-25 | 8.33E-25 |
| rs1391438 | -0.031091535 | 0.002921924 | 1.93E-26 | 8.33E-25 |
| rs1452075 | -0.030577958 | 0.002952237 | 3.87E-25 | 8.33E-25 |
| rs145590108 | -0.030493269 | 0.002953906 | 5.54E-25 | 8.33E-25 |
| rs1566085 | -0.03072221 | 0.002956161 | 2.68E-25 | 8.33E-25 |
| rs1569266 | -0.030663583 | 0.002949407 | 2.57E-25 | 8.33E-25 |
| rs1620977 | -0.030608806 | 0.002960229 | 4.64E-25 | 8.33E-25 |
| rs1689510 | -0.030123186 | 0.002934792 | 1.02E-24 | 1.06E-24 |
| rs17489649 | -0.030300875 | 0.002942216 | 7.15E-25 | 8.41E-25 |
| rs17513684 | -0.030479 | 0.002952766 | 5.59E-25 | 8.33E-25 |
| rs175325 | -0.030585722 | 0.002952724 | 3.83E-25 | 8.33E-25 |
| rs17563464 | -0.03046372 | 0.002955257 | 6.46E-25 | 8.33E-25 |
| rs17628095 | -0.030445331 | 0.002952485 | 6.23E-25 | 8.33E-25 |
| rs1788783 | -0.030782883 | 0.002945029 | 1.43E-25 | 8.33E-25 |
| rs1812587 | -0.030417247 | 0.002950735 | 6.46E-25 | 8.33E-25 |
| rs1835340 | -0.030476402 | 0.002952355 | 5.56E-25 | 8.33E-25 |
| rs185291 | -0.030304462 | 0.002961994 | 1.44E-24 | 1.46E-24 |
| rs1869165 | -0.030501363 | 0.00295286 | 5.19E-25 | 8.33E-25 |
| rs1880692 | -0.030665996 | 0.00294753 | 2.38E-25 | 8.33E-25 |
| rs1892417 | -0.031078516 | 0.002904779 | 1.03E-26 | 8.33E-25 |
| rs1917008 | -0.030490608 | 0.002952463 | 5.31E-25 | 8.33E-25 |
| rs192436652 | -0.030493683 | 0.002953635 | 5.48E-25 | 8.33E-25 |
| rs1964927 | -0.030440401 | 0.002952668 | 6.39E-25 | 8.33E-25 |
| rs1980251 | -0.030781603 | 0.002953036 | 1.93E-25 | 8.33E-25 |
| rs2145265 | -0.030616572 | 0.002950717 | 3.19E-25 | 8.33E-25 |
| rs215632 | -0.030358639 | 0.002946818 | 6.89E-25 | 8.33E-25 |
| rs2175420 | -0.030580969 | 0.002952945 | 3.93E-25 | 8.33E-25 |
| rs2182398 | -0.030614017 | 0.002950482 | 3.19E-25 | 8.33E-25 |
| rs2190872 | -0.030776257 | 0.002937033 | 1.08E-25 | 8.33E-25 |
| rs2287838 | -0.030658432 | 0.002948247 | 2.51E-25 | 8.33E-25 |
| rs2299098 | -0.030529077 | 0.002957086 | 5.49E-25 | 8.33E-25 |
| rs2309812 | -0.030380574 | 0.002959864 | 1.02E-24 | 1.06E-24 |
| rs2332818 | -0.030316794 | 0.00294224 | 6.76E-25 | 8.33E-25 |
| rs2411453 | -0.030736102 | 0.002953548 | 2.32E-25 | 8.33E-25 |
| rs2559509 | -0.030460612 | 0.002952935 | 6.00E-25 | 8.33E-25 |
| rs2570497 | -0.030212265 | 0.002936973 | 8.08E-25 | 8.96E-25 |
| rs2604541 | -0.030744409 | 0.002940141 | 1.36E-25 | 8.33E-25 |
| rs2706762 | -0.030682335 | 0.002948155 | 2.30E-25 | 8.33E-25 |
| rs2725371 | -0.03042591 | 0.002954134 | 7.09E-25 | 8.39E-25 |
| rs2735421 | -0.030275908 | 0.002953625 | 1.18E-24 | 1.21E-24 |
| rs281324 | -0.03002668 | 0.00289458 | 3.28E-25 | 8.33E-25 |
| rs2820313 | -0.030391147 | 0.002949031 | 6.66E-25 | 8.33E-25 |
| rs2834011 | -0.030718299 | 0.002944747 | 1.78E-25 | 8.33E-25 |
| rs2974312 | -0.030327697 | 0.002949368 | 8.43E-25 | 9.30E-25 |
| rs2998309 | -0.030573639 | 0.002951823 | 3.87E-25 | 8.33E-25 |
| rs324801 | -0.030275762 | 0.00293859 | 6.84E-25 | 8.33E-25 |
| rs333078 | -0.030354167 | 0.00294623 | 6.85E-25 | 8.33E-25 |
| rs34042385 | -0.030286885 | 0.002939253 | 6.74E-25 | 8.33E-25 |
| rs34192341 | -0.030670103 | 0.002948344 | 2.42E-25 | 8.33E-25 |
| rs34364916 | -0.030685442 | 0.002946322 | 2.12E-25 | 8.33E-25 |
| rs34470581 | -0.029999722 | 0.002905371 | 5.40E-25 | 8.33E-25 |
| rs34945223 | -0.030241397 | 0.002934169 | 6.58E-25 | 8.33E-25 |
| rs35039375 | -0.030493055 | 0.002954337 | 5.64E-25 | 8.33E-25 |
| rs35091253 | -0.030435173 | 0.002955643 | 7.25E-25 | 8.44E-25 |
| rs35811586 | -0.030609262 | 0.002950864 | 3.29E-25 | 8.33E-25 |
| rs35917528 | -0.030520251 | 0.002953191 | 4.91E-25 | 8.33E-25 |
| rs35999162 | -0.030442224 | 0.002974521 | 1.39E-24 | 1.42E-24 |
| rs363096 | -0.030677248 | 0.002950006 | 2.50E-25 | 8.33E-25 |
| rs3747631 | -0.030422841 | 0.002959145 | 8.59E-25 | 9.33E-25 |
| rs3788556 | -0.03068892 | 0.002949491 | 2.36E-25 | 8.33E-25 |
| rs3794620 | -0.030427462 | 0.002952542 | 6.65E-25 | 8.33E-25 |
| rs3800925 | -0.030735695 | 0.002949237 | 1.98E-25 | 8.33E-25 |
| rs3825083 | -0.0304957 | 0.002954127 | 5.54E-25 | 8.33E-25 |
| rs3827531 | -0.03057751 | 0.002951971 | 3.84E-25 | 8.33E-25 |
| rs3847225 | -0.03085209 | 0.002949128 | 1.30E-25 | 8.33E-25 |
| rs3943093 | -0.030817978 | 0.002946386 | 1.32E-25 | 8.33E-25 |
| rs4130477 | -0.030495594 | 0.002952499 | 5.22E-25 | 8.33E-25 |
| rs4146675 | -0.030654692 | 0.002948287 | 2.55E-25 | 8.33E-25 |
| rs417968 | -0.0310499 | 0.002925342 | 2.56E-26 | 8.33E-25 |
| rs42210 | -0.030394951 | 0.00294873 | 6.50E-25 | 8.33E-25 |
| rs4246167 | -0.030291436 | 0.002947183 | 8.85E-25 | 9.52E-25 |
| rs4700393 | -0.030244573 | 0.002964705 | 1.95E-24 | 1.95E-24 |
| rs4726070 | -0.030571207 | 0.002953989 | 4.22E-25 | 8.33E-25 |
| rs4731992 | -0.030361016 | 0.00295508 | 9.21E-25 | 9.72E-25 |
| rs4757957 | -0.03031044 | 0.002945416 | 7.76E-25 | 8.80E-25 |
| rs4780563 | -0.03065327 | 0.002949144 | 2.64E-25 | 8.33E-25 |
| rs4808766 | -0.030629956 | 0.002949594 | 2.92E-25 | 8.33E-25 |
| rs4958568 | -0.030780148 | 0.00293906 | 1.15E-25 | 8.33E-25 |
| rs55800473 | -0.030587981 | 0.002953312 | 3.88E-25 | 8.33E-25 |
| rs55842281 | -0.030800294 | 0.002938191 | 1.04E-25 | 8.33E-25 |
| rs55859553 | -0.030510724 | 0.002952975 | 5.04E-25 | 8.33E-25 |
| rs55872852 | -0.030520586 | 0.002952896 | 4.85E-25 | 8.33E-25 |
| rs56118554 | -0.030782657 | 0.002948572 | 1.63E-25 | 8.33E-25 |
| rs575113 | -0.03069215 | 0.002945399 | 2.00E-25 | 8.33E-25 |
| rs59123361 | -0.030600707 | 0.002954567 | 3.89E-25 | 8.33E-25 |
| rs6071573 | -0.030894116 | 0.002932342 | 5.92E-26 | 8.33E-25 |
| rs613872 | -0.030447302 | 0.00295495 | 6.77E-25 | 8.33E-25 |
| rs61787087 | -0.030822828 | 0.00292871 | 6.67E-26 | 8.33E-25 |
| rs61787785 | -0.030399494 | 0.002951467 | 7.06E-25 | 8.39E-25 |
| rs61868084 | -0.03039821 | 0.002950014 | 6.73E-25 | 8.33E-25 |
| rs62018215 | -0.03050631 | 0.002952617 | 5.05E-25 | 8.33E-25 |
| rs62182125 | -0.030566151 | 0.00295213 | 4.02E-25 | 8.33E-25 |
| rs62184483 | -0.030552472 | 0.002958553 | 5.33E-25 | 8.33E-25 |
| rs62253608 | -0.030369866 | 0.002949041 | 7.18E-25 | 8.41E-25 |
| rs62389638 | -0.030441933 | 0.002954354 | 6.75E-25 | 8.33E-25 |
| rs6429911 | -0.03059801 | 0.00295285 | 3.68E-25 | 8.33E-25 |
| rs6556982 | -0.030594346 | 0.002951367 | 3.53E-25 | 8.33E-25 |
| rs660001 | -0.030748815 | 0.002947187 | 1.75E-25 | 8.33E-25 |
| rs6682095 | -0.030573793 | 0.00295435 | 4.24E-25 | 8.33E-25 |
| rs66844142 | -0.030658386 | 0.002948003 | 2.49E-25 | 8.33E-25 |
| rs6760772 | -0.030528095 | 0.002952897 | 4.73E-25 | 8.33E-25 |
| rs67651814 | -0.030385686 | 0.002951083 | 7.31E-25 | 8.47E-25 |
| rs6779254 | -0.030483286 | 0.002955033 | 5.98E-25 | 8.33E-25 |
| rs6789699 | -0.03049429 | 0.002953607 | 5.46E-25 | 8.33E-25 |
| rs67944653 | -0.030475581 | 0.002952892 | 5.69E-25 | 8.33E-25 |
| rs6935954 | -0.030433154 | 0.002961266 | 8.94E-25 | 9.56E-25 |
| rs6959579 | -0.030565197 | 0.00295239 | 4.07E-25 | 8.33E-25 |
| rs702606 | -0.030443875 | 0.002951637 | 6.08E-25 | 8.33E-25 |
| rs7031698 | -0.030367925 | 0.00294744 | 6.82E-25 | 8.33E-25 |
| rs7070693 | -0.030361958 | 0.002953187 | 8.58E-25 | 9.33E-25 |
| rs711793 | -0.030718132 | 0.002944051 | 1.73E-25 | 8.33E-25 |
| rs71646142 | -0.030655891 | 0.002949125 | 2.62E-25 | 8.33E-25 |
| rs7195278 | -0.030693754 | 0.002951136 | 2.46E-25 | 8.33E-25 |
| rs7233920 | -0.03043509 | 0.002954095 | 6.85E-25 | 8.33E-25 |
| rs72674898 | -0.030319749 | 0.00294352 | 7.01E-25 | 8.39E-25 |
| rs72807818 | -0.030401237 | 0.002950244 | 6.71E-25 | 8.33E-25 |
| rs72828517 | -0.030918 | 0.002938663 | 6.91E-26 | 8.33E-25 |
| rs72977992 | -0.030610138 | 0.002950731 | 3.26E-25 | 8.33E-25 |
| rs73040036 | -0.030460418 | 0.002951967 | 5.80E-25 | 8.33E-25 |
| rs73499064 | -0.030566244 | 0.002953367 | 4.20E-25 | 8.33E-25 |
| rs75033012 | -0.030713781 | 0.002946845 | 1.96E-25 | 8.33E-25 |
| rs7526112 | -0.030799112 | 0.002944865 | 1.34E-25 | 8.33E-25 |
| rs7531271 | -0.03056041 | 0.002958319 | 5.14E-25 | 8.33E-25 |
| rs75433564 | -0.030308007 | 0.002943885 | 7.40E-25 | 8.51E-25 |
| rs7548936 | -0.030040784 | 0.002932816 | 1.27E-24 | 1.30E-24 |
| rs7580304 | -0.030535328 | 0.002952492 | 4.54E-25 | 8.33E-25 |
| rs7583473 | -0.03010912 | 0.002919349 | 6.11E-25 | 8.33E-25 |
| rs7598246 | -0.030463098 | 0.002953526 | 6.08E-25 | 8.33E-25 |
| rs7629643 | -0.030436217 | 0.002950941 | 6.09E-25 | 8.33E-25 |
| rs76608582 | -0.030017034 | 0.002900233 | 4.19E-25 | 8.33E-25 |
| rs7675394 | -0.030810323 | 0.002945914 | 1.34E-25 | 8.33E-25 |
| rs76878669 | -0.030670201 | 0.002948 | 2.38E-25 | 8.33E-25 |
| rs77025239 | -0.030506797 | 0.002953251 | 5.16E-25 | 8.33E-25 |
| rs7758776 | -0.030492305 | 0.002953224 | 5.43E-25 | 8.33E-25 |
| rs77675579 | -0.030620006 | 0.002951853 | 3.29E-25 | 8.33E-25 |
| rs7768116 | -0.030392011 | 0.002949037 | 6.64E-25 | 8.33E-25 |
| rs781289 | -0.030701797 | 0.002949738 | 2.27E-25 | 8.33E-25 |
| rs78452560 | -0.030376782 | 0.002951056 | 7.54E-25 | 8.59E-25 |
| rs7868164 | -0.030440527 | 0.00295083 | 5.97E-25 | 8.33E-25 |
| rs7868984 | -0.030997173 | 0.002956062 | 1.00E-25 | 8.33E-25 |
| rs7873964 | -0.030407204 | 0.002951194 | 6.80E-25 | 8.33E-25 |
| rs7966054 | -0.030477363 | 0.002953095 | 5.69E-25 | 8.33E-25 |
| rs7977614 | -0.030236052 | 0.002936996 | 7.43E-25 | 8.51E-25 |
| rs7987170 | -0.030583259 | 0.002952717 | 3.86E-25 | 8.33E-25 |
| rs7988201 | -0.030283697 | 0.002943541 | 7.97E-25 | 8.96E-25 |
| rs7988627 | -0.030468639 | 0.002952877 | 5.83E-25 | 8.33E-25 |
| rs79937071 | -0.030452501 | 0.002951876 | 5.95E-25 | 8.33E-25 |
| rs8008382 | -0.0306854 | 0.00294683 | 2.16E-25 | 8.33E-25 |
| rs8020034 | -0.030471298 | 0.002955123 | 6.26E-25 | 8.33E-25 |
| rs8057808 | -0.030523021 | 0.002954116 | 5.03E-25 | 8.33E-25 |
| rs807478 | -0.030356727 | 0.002946736 | 6.91E-25 | 8.33E-25 |
| rs837065 | -0.030565545 | 0.002955074 | 4.48E-25 | 8.33E-25 |
| rs868698 | -0.030520301 | 0.002954063 | 5.07E-25 | 8.33E-25 |
| rs879394 | -0.030505867 | 0.002952902 | 5.11E-25 | 8.33E-25 |
| rs9372625 | -0.031075598 | 0.002949543 | 5.91E-26 | 8.33E-25 |
| rs9643120 | -0.030434428 | 0.002953082 | 6.62E-25 | 8.33E-25 |
| rs9797233 | -0.030500339 | 0.002952582 | 5.15E-25 | 8.33E-25 |
| rs9888796 | -0.030636075 | 0.00295117 | 3.03E-25 | 8.33E-25 |
| All | -0.0305215 | 0.002940883 | 3.11E-25 | 8.33E-25 |
