# Supplementary material for: Causal roles of educational duration in bone mineral density and risk factors for osteoporosis: a Mendelian randomization study
Source: BMC Musculoskelet Disord. 2024 May 2;25:345. doi: 10.1186/s12891-024-07428-8 (PMC11064366; doi:10.1186/s12891-024-07428-8)
Supplement: Supplementary file 1 — Supplementary Material 1. [file 12891_2024_7428_MOESM1_ESM.zip › IVs of Educational attainment on sunbath.docx]

| SNP | b | se | P.value | adjust P.value |
| --- | --- | --- | --- | --- |
| rs10058365 | -0.012711656 | 0.000973584 | 5.83E-39 | 8.73E-39 |
| rs10066409 | -0.012737102 | 0.000971639 | 2.93E-39 | 8.73E-39 |
| rs1010334 | -0.012729037 | 0.000971609 | 3.25E-39 | 8.73E-39 |
| rs10189857 | -0.012690361 | 0.000973628 | 7.83E-39 | 9.17E-39 |
| rs10215082 | -0.012725881 | 0.00097187 | 3.55E-39 | 8.73E-39 |
| rs1050847 | -0.012679011 | 0.000971677 | 6.47E-39 | 8.73E-39 |
| rs10511592 | -0.012754432 | 0.00097085 | 2.01E-39 | 8.69E-39 |
| rs10518019 | -0.012773811 | 0.000971525 | 1.74E-39 | 8.54E-39 |
| rs10745789 | -0.012702322 | 0.000971993 | 4.99E-39 | 8.73E-39 |
| rs10760023 | -0.012668672 | 0.000970875 | 6.46E-39 | 8.73E-39 |
| rs10765775 | -0.012720261 | 0.000973534 | 5.15E-39 | 8.73E-39 |
| rs10844179 | -0.012659961 | 0.000970778 | 7.15E-39 | 8.82E-39 |
| rs10854884 | -0.012986506 | 0.000942553 | 3.46E-43 | 7.33E-41 |
| rs10994777 | -0.012667633 | 0.000972195 | 8.27E-39 | 9.36E-39 |
| rs11138947 | -0.012673461 | 0.000971356 | 6.59E-39 | 8.73E-39 |
| rs11155821 | -0.012539753 | 0.000959682 | 5.11E-39 | 8.73E-39 |
| rs11214468 | -0.012656233 | 0.000970777 | 7.51E-39 | 9.00E-39 |
| rs11243838 | -0.012700683 | 0.000972095 | 5.20E-39 | 8.73E-39 |
| rs11249939 | -0.012540673 | 0.000962659 | 8.58E-39 | 9.57E-39 |
| rs11572842 | -0.012748326 | 0.00097049 | 2.05E-39 | 8.69E-39 |
| rs115877304 | -0.012635691 | 0.000968942 | 7.18E-39 | 8.82E-39 |
| rs11604034 | -0.012686798 | 0.000972495 | 6.73E-39 | 8.73E-39 |
| rs11635966 | -0.012692772 | 0.000972302 | 6.00E-39 | 8.73E-39 |
| rs11661305 | -0.012719806 | 0.000972448 | 4.27E-39 | 8.73E-39 |
| rs11678980 | -0.012582248 | 0.000969647 | 1.67E-38 | 1.70E-38 |
| rs11690224 | -0.012676773 | 0.000971425 | 6.38E-39 | 8.73E-39 |
| rs11693764 | -0.012723218 | 0.000971826 | 3.65E-39 | 8.73E-39 |
| rs11714679 | -0.012698845 | 0.000972022 | 5.26E-39 | 8.73E-39 |
| rs11720121 | -0.012620131 | 0.000970138 | 1.09E-38 | 1.16E-38 |
| rs11732657 | -0.012656619 | 0.000970168 | 6.71E-39 | 8.73E-39 |
| rs11736863 | -0.012629285 | 0.000970251 | 9.86E-39 | 1.07E-38 |
| rs11764590 | -0.01271141 | 0.000972603 | 4.92E-39 | 8.73E-39 |
| rs117799466 | -0.012800636 | 0.000965926 | 4.38E-40 | 7.02E-39 |
| rs118083122 | -0.012781183 | 0.000968224 | 8.70E-40 | 7.50E-39 |
| rs11871429 | -0.012715775 | 0.000972227 | 4.34E-39 | 8.73E-39 |
| rs11915747 | -0.012610003 | 0.000970968 | 1.45E-38 | 1.48E-38 |
| rs12029988 | -0.012815669 | 0.000965004 | 3.01E-40 | 7.02E-39 |
| rs12076635 | -0.012530479 | 0.0009657 | 1.68E-38 | 1.71E-38 |
| rs12132451 | -0.01272832 | 0.000972627 | 3.93E-39 | 8.73E-39 |
| rs12468040 | -0.012546989 | 0.000964607 | 1.11E-38 | 1.17E-38 |
| rs12474895 | -0.0126775 | 0.000971626 | 6.55E-39 | 8.73E-39 |
| rs12503522 | -0.012735118 | 0.000971255 | 2.81E-39 | 8.73E-39 |
| rs12532494 | -0.012717476 | 0.000973437 | 5.25E-39 | 8.73E-39 |
| rs12574281 | -0.012735231 | 0.000971379 | 2.87E-39 | 8.73E-39 |
| rs12663818 | -0.012700207 | 0.000972006 | 5.15E-39 | 8.73E-39 |
| rs12735232 | -0.012678774 | 0.00097187 | 6.72E-39 | 8.73E-39 |
| rs12804787 | -0.012750679 | 0.000970349 | 1.93E-39 | 8.54E-39 |
| rs12921005 | -0.01268711 | 0.00097169 | 5.82E-39 | 8.73E-39 |
| rs12967855 | -0.012689387 | 0.00097629 | 1.26E-38 | 1.31E-38 |
| rs1334297 | -0.012775275 | 0.000974024 | 2.67E-39 | 8.73E-39 |
| rs13409451 | -0.012685303 | 0.000973574 | 8.30E-39 | 9.36E-39 |
| rs1363862 | -0.012577074 | 0.000959055 | 2.74E-39 | 8.73E-39 |
| rs1369128 | -0.012684604 | 0.000972146 | 6.52E-39 | 8.73E-39 |
| rs1381247 | -0.012740413 | 0.000971016 | 2.51E-39 | 8.73E-39 |
| rs1391438 | -0.012833723 | 0.000968264 | 4.26E-40 | 7.02E-39 |
| rs1452075 | -0.012719904 | 0.000971906 | 3.88E-39 | 8.73E-39 |
| rs145590108 | -0.01269692 | 0.000972353 | 5.73E-39 | 8.73E-39 |
| rs1566085 | -0.012754297 | 0.000973684 | 3.34E-39 | 8.73E-39 |
| rs1569266 | -0.012661463 | 0.000971 | 7.28E-39 | 8.82E-39 |
| rs1620977 | -0.012825953 | 0.000970562 | 7.19E-40 | 7.50E-39 |
| rs1689510 | -0.012615677 | 0.000969829 | 1.10E-38 | 1.17E-38 |
| rs17489649 | -0.012642365 | 0.00096934 | 7.04E-39 | 8.82E-39 |
| rs17513684 | -0.012695891 | 0.000972041 | 5.50E-39 | 8.73E-39 |
| rs175325 | -0.012788602 | 0.000967851 | 7.34E-40 | 7.50E-39 |
| rs17563464 | -0.012785816 | 0.000969969 | 1.12E-39 | 8.22E-39 |
| rs17628095 | -0.012706944 | 0.000972278 | 4.93E-39 | 8.73E-39 |
| rs1788783 | -0.01273522 | 0.000972522 | 3.51E-39 | 8.73E-39 |
| rs1812587 | -0.012677724 | 0.000971571 | 6.46E-39 | 8.73E-39 |
| rs1835340 | -0.012700013 | 0.000971979 | 5.14E-39 | 8.73E-39 |
| rs185291 | -0.012894726 | 0.000968514 | 1.92E-40 | 7.02E-39 |
| rs1869165 | -0.012688677 | 0.000971825 | 5.83E-39 | 8.73E-39 |
| rs1880692 | -0.01276987 | 0.000968844 | 1.14E-39 | 8.22E-39 |
| rs1892417 | -0.012698393 | 0.000972779 | 6.05E-39 | 8.73E-39 |
| rs1917008 | -0.012779833 | 0.000967794 | 8.20E-40 | 7.50E-39 |
| rs192436652 | -0.012724971 | 0.00097209 | 3.74E-39 | 8.73E-39 |
| rs1964927 | -0.012714147 | 0.000972335 | 4.52E-39 | 8.73E-39 |
| rs1980251 | -0.012841525 | 0.000968655 | 4.11E-40 | 7.02E-39 |
| rs2145265 | -0.012737962 | 0.000971269 | 2.71E-39 | 8.73E-39 |
| rs215632 | -0.012615147 | 0.000966171 | 5.81E-39 | 8.73E-39 |
| rs2175420 | -0.012755926 | 0.000970684 | 1.91E-39 | 8.54E-39 |
| rs2182398 | -0.012794903 | 0.000965801 | 4.64E-40 | 7.02E-39 |
| rs2190872 | -0.012800771 | 0.00096532 | 3.91E-40 | 7.02E-39 |
| rs2287838 | -0.012746631 | 0.000970765 | 2.20E-39 | 8.73E-39 |
| rs2299098 | -0.012823671 | 0.00096788 | 4.56E-40 | 7.02E-39 |
| rs2309812 | -0.01265744 | 0.00097423 | 1.35E-38 | 1.39E-38 |
| rs2332818 | -0.012704355 | 0.000971961 | 4.83E-39 | 8.73E-39 |
| rs2411453 | -0.012817946 | 0.000969649 | 6.80E-40 | 7.50E-39 |
| rs2559509 | -0.012694391 | 0.0009722 | 5.77E-39 | 8.73E-39 |
| rs2570497 | -0.01268117 | 0.000972188 | 6.88E-39 | 8.73E-39 |
| rs2604541 | -0.01269551 | 0.000971865 | 5.35E-39 | 8.73E-39 |
| rs2706762 | -0.012580148 | 0.000961986 | 4.44E-39 | 8.73E-39 |
| rs2725371 | -0.01277822 | 0.000970388 | 1.34E-39 | 8.35E-39 |
| rs2735421 | -0.01268318 | 0.0009741 | 9.37E-39 | 1.02E-38 |
| rs281324 | -0.012619138 | 0.000966619 | 5.96E-39 | 8.73E-39 |
| rs2820313 | -0.012668487 | 0.000971061 | 6.69E-39 | 8.73E-39 |
| rs2834011 | -0.012770992 | 0.000969305 | 1.22E-39 | 8.22E-39 |
| rs2974312 | -0.012620581 | 0.000969281 | 9.35E-39 | 1.02E-38 |
| rs2998309 | -0.012619491 | 0.000965987 | 5.30E-39 | 8.73E-39 |
| rs324801 | -0.012657878 | 0.000970375 | 6.85E-39 | 8.73E-39 |
| rs333078 | -0.012693914 | 0.00097192 | 5.52E-39 | 8.73E-39 |
| rs34042385 | -0.012667238 | 0.000970867 | 6.58E-39 | 8.73E-39 |
| rs34192341 | -0.012697452 | 0.0009721 | 5.44E-39 | 8.73E-39 |
| rs34364916 | -0.012766211 | 0.000969304 | 1.30E-39 | 8.34E-39 |
| rs34470581 | -0.012596936 | 0.000966046 | 7.28E-39 | 8.82E-39 |
| rs34945223 | -0.012579971 | 0.000960459 | 3.39E-39 | 8.73E-39 |
| rs35039375 | -0.012712754 | 0.000972505 | 4.75E-39 | 8.73E-39 |
| rs35091253 | -0.012703783 | 0.000973252 | 6.12E-39 | 8.73E-39 |
| rs35811586 | -0.012715322 | 0.000971905 | 4.12E-39 | 8.73E-39 |
| rs35917528 | -0.012699016 | 0.000972084 | 5.31E-39 | 8.73E-39 |
| rs35999162 | -0.012662459 | 0.000978932 | 2.86E-38 | 2.88E-38 |
| rs363096 | -0.01274849 | 0.000971511 | 2.45E-39 | 8.73E-39 |
| rs3747631 | -0.012679121 | 0.000974155 | 9.99E-39 | 1.07E-38 |
| rs3788556 | -0.012604463 | 0.000966913 | 7.66E-39 | 9.12E-39 |
| rs3794620 | -0.012673587 | 0.000971865 | 7.20E-39 | 8.82E-39 |
| rs3800925 | -0.012659888 | 0.000972086 | 9.00E-39 | 9.99E-39 |
| rs3825083 | -0.012762818 | 0.000970593 | 1.71E-39 | 8.54E-39 |
| rs3827531 | -0.012759375 | 0.000969793 | 1.56E-39 | 8.54E-39 |
| rs3847225 | -0.012720841 | 0.000974276 | 5.82E-39 | 8.73E-39 |
| rs3943093 | -0.012846763 | 0.000965848 | 2.28E-40 | 7.02E-39 |
| rs4130477 | -0.01268023 | 0.000971439 | 6.11E-39 | 8.73E-39 |
| rs4146675 | -0.012645958 | 0.00096922 | 6.56E-39 | 8.73E-39 |
| rs417968 | -0.012807177 | 0.000970115 | 8.58E-40 | 7.50E-39 |
| rs42210 | -0.012673719 | 0.000971177 | 6.37E-39 | 8.73E-39 |
| rs4246167 | -0.012734078 | 0.0009725 | 3.56E-39 | 8.73E-39 |
| rs4700393 | -0.012568981 | 0.00097406 | 4.29E-38 | 4.29E-38 |
| rs4726070 | -0.012769556 | 0.000970253 | 1.47E-39 | 8.54E-39 |
| rs4731992 | -0.012623138 | 0.000971263 | 1.28E-38 | 1.32E-38 |
| rs4757957 | -0.012644208 | 0.000970154 | 7.92E-39 | 9.23E-39 |
| rs4780563 | -0.012780115 | 0.000968235 | 8.84E-40 | 7.50E-39 |
| rs4808766 | -0.012786087 | 0.000966915 | 6.41E-40 | 7.50E-39 |
| rs4958568 | -0.012579044 | 0.000961584 | 4.20E-39 | 8.73E-39 |
| rs55800473 | -0.012720013 | 0.00097232 | 4.17E-39 | 8.73E-39 |
| rs55842281 | -0.012759544 | 0.000970562 | 1.78E-39 | 8.54E-39 |
| rs55859553 | -0.012641603 | 0.00096908 | 6.79E-39 | 8.73E-39 |
| rs55872852 | -0.012679161 | 0.000971495 | 6.26E-39 | 8.73E-39 |
| rs56118554 | -0.012825286 | 0.000967891 | 4.47E-40 | 7.02E-39 |
| rs575113 | -0.01268973 | 0.00097175 | 5.67E-39 | 8.73E-39 |
| rs59123361 | -0.012667288 | 0.00097217 | 8.27E-39 | 9.36E-39 |
| rs6071573 | -0.012763582 | 0.000971117 | 1.86E-39 | 8.54E-39 |
| rs613872 | -0.012694052 | 0.000972905 | 6.56E-39 | 8.73E-39 |
| rs61787087 | -0.012703792 | 0.000971881 | 4.80E-39 | 8.73E-39 |
| rs61787785 | -0.012631141 | 0.000969232 | 8.03E-39 | 9.26E-39 |
| rs61868084 | -0.012701483 | 0.000972156 | 5.20E-39 | 8.73E-39 |
| rs62018215 | -0.012710778 | 0.000971932 | 4.41E-39 | 8.73E-39 |
| rs62182125 | -0.012711287 | 0.000971906 | 4.36E-39 | 8.73E-39 |
| rs62184483 | -0.012640057 | 0.000972422 | 1.25E-38 | 1.30E-38 |
| rs62253608 | -0.012852405 | 0.000958749 | 5.62E-41 | 5.96E-39 |
| rs62389638 | -0.012616828 | 0.000968955 | 9.29E-39 | 1.02E-38 |
| rs6429911 | -0.01265354 | 0.000970727 | 7.73E-39 | 9.15E-39 |
| rs6556982 | -0.012715142 | 0.000971886 | 4.12E-39 | 8.73E-39 |
| rs660001 | -0.012663786 | 0.000971982 | 8.39E-39 | 9.41E-39 |
| rs6682095 | -0.012663312 | 0.000971685 | 8.01E-39 | 9.26E-39 |
| rs66844142 | -0.012746096 | 0.000970721 | 2.20E-39 | 8.73E-39 |
| rs6760772 | -0.0126802 | 0.000971535 | 6.21E-39 | 8.73E-39 |
| rs67651814 | -0.012715524 | 0.000972487 | 4.56E-39 | 8.73E-39 |
| rs6779254 | -0.012731209 | 0.000972478 | 3.68E-39 | 8.73E-39 |
| rs6789699 | -0.012707682 | 0.000972296 | 4.90E-39 | 8.73E-39 |
| rs67944653 | -0.012661645 | 0.000970868 | 7.10E-39 | 8.82E-39 |
| rs6935954 | -0.012589235 | 0.000973362 | 2.90E-38 | 2.92E-38 |
| rs6959579 | -0.012690196 | 0.000971823 | 5.71E-39 | 8.73E-39 |
| rs702606 | -0.012604696 | 0.000964746 | 5.20E-39 | 8.73E-39 |
| rs7031698 | -0.012668061 | 0.000971026 | 6.69E-39 | 8.73E-39 |
| rs7070693 | -0.012641481 | 0.000971527 | 1.05E-38 | 1.12E-38 |
| rs711793 | -0.01269918 | 0.000972033 | 5.25E-39 | 8.73E-39 |
| rs71646142 | -0.012783282 | 0.00096796 | 8.06E-40 | 7.50E-39 |
| rs7195278 | -0.012771264 | 0.000970936 | 1.62E-39 | 8.54E-39 |
| rs7233920 | -0.01258368 | 0.000965412 | 7.79E-39 | 9.17E-39 |
| rs72674898 | -0.012661484 | 0.00097066 | 6.86E-39 | 8.73E-39 |
| rs72807818 | -0.012626147 | 0.000967914 | 6.81E-39 | 8.73E-39 |
| rs72828517 | -0.012798518 | 0.000970434 | 1.02E-39 | 8.22E-39 |
| rs72977992 | -0.01274126 | 0.000971002 | 2.47E-39 | 8.73E-39 |
| rs73040036 | -0.01265126 | 0.000969807 | 6.77E-39 | 8.73E-39 |
| rs73499064 | -0.012759098 | 0.000970552 | 1.79E-39 | 8.54E-39 |
| rs75033012 | -0.012767077 | 0.000970156 | 1.49E-39 | 8.54E-39 |
| rs7526112 | -0.012717019 | 0.000973014 | 4.90E-39 | 8.73E-39 |
| rs7531271 | -0.012721333 | 0.000973761 | 5.28E-39 | 8.73E-39 |
| rs75433564 | -0.012693537 | 0.00097214 | 5.77E-39 | 8.73E-39 |
| rs7548936 | -0.012644098 | 0.000972594 | 1.22E-38 | 1.28E-38 |
| rs7580304 | -0.012659604 | 0.000970201 | 6.49E-39 | 8.73E-39 |
| rs7583473 | -0.012797228 | 0.000967147 | 5.74E-40 | 7.50E-39 |
| rs7598246 | -0.012703168 | 0.000972428 | 5.33E-39 | 8.73E-39 |
| rs7629643 | -0.012671488 | 0.000971078 | 6.45E-39 | 8.73E-39 |
| rs76608582 | -0.012691564 | 0.000972139 | 5.93E-39 | 8.73E-39 |
| rs7675394 | -0.012747692 | 0.00097264 | 3.03E-39 | 8.73E-39 |
| rs76878669 | -0.012780033 | 0.000968201 | 8.80E-40 | 7.50E-39 |
| rs77025239 | -0.012721536 | 0.000971982 | 3.84E-39 | 8.73E-39 |
| rs7758776 | -0.01266851 | 0.000971261 | 6.93E-39 | 8.75E-39 |
| rs77675579 | -0.012827307 | 0.000963202 | 1.83E-40 | 7.02E-39 |
| rs7768116 | -0.012763713 | 0.000969645 | 1.43E-39 | 8.54E-39 |
| rs781289 | -0.012768145 | 0.00097078 | 1.65E-39 | 8.54E-39 |
| rs78452560 | -0.012706692 | 0.00097262 | 5.26E-39 | 8.73E-39 |
| rs7868164 | -0.012732321 | 0.000971347 | 2.97E-39 | 8.73E-39 |
| rs7868984 | -0.012770082 | 0.000976572 | 4.49E-39 | 8.73E-39 |
| rs7873964 | -0.012712623 | 0.000972299 | 4.59E-39 | 8.73E-39 |
| rs7966054 | -0.0126763 | 0.000971651 | 6.69E-39 | 8.73E-39 |
| rs7977614 | -0.012774582 | 0.00096934 | 1.16E-39 | 8.22E-39 |
| rs7987170 | -0.012756799 | 0.000970549 | 1.84E-39 | 8.54E-39 |
| rs7988201 | -0.01263783 | 0.000969801 | 8.11E-39 | 9.30E-39 |
| rs7988627 | -0.012706458 | 0.000972212 | 4.91E-39 | 8.73E-39 |
| rs79937071 | -0.01273773 | 0.000971322 | 2.74E-39 | 8.73E-39 |
| rs8008382 | -0.012741518 | 0.000971179 | 2.54E-39 | 8.73E-39 |
| rs8020034 | -0.012751622 | 0.000971879 | 2.51E-39 | 8.73E-39 |
| rs8057808 | -0.012666735 | 0.00097152 | 7.43E-39 | 8.95E-39 |
| rs807478 | -0.012616616 | 0.000966427 | 5.96E-39 | 8.73E-39 |
| rs837065 | -0.012634813 | 0.000970367 | 9.34E-39 | 1.02E-38 |
| rs868698 | -0.012774067 | 0.000969651 | 1.24E-39 | 8.22E-39 |
| rs879394 | -0.012691144 | 0.000971881 | 5.70E-39 | 8.73E-39 |
| rs9372625 | -0.012802715 | 0.000975253 | 2.29E-39 | 8.73E-39 |
| rs9643120 | -0.012702239 | 0.000972532 | 5.50E-39 | 8.73E-39 |
| rs9797233 | -0.012825197 | 0.000961016 | 1.26E-40 | 7.02E-39 |
| rs9888796 | -0.012778453 | 0.000969076 | 1.05E-39 | 8.22E-39 |
| All | -0.012705858 | 0.000968074 | 2.37E-39 | 8.73E-39 |
