# Supplementary material for: Causal roles of educational duration in bone mineral density and risk factors for osteoporosis: a Mendelian randomization study
Source: BMC Musculoskelet Disord. 2024 May 2;25:345. doi: 10.1186/s12891-024-07428-8 (PMC11064366; doi:10.1186/s12891-024-07428-8)
Supplement: Supplementary file 1 — Supplementary Material 1. [file 12891_2024_7428_MOESM1_ESM.zip › IVs of Educational attainment on time for light physical activity.docx]

| SNP | b | se | P.value | adjust P.value |
| --- | --- | --- | --- | --- |
| rs10058365 | -0.505171736 | 0.498764682 | 0.311133799 | 0.40889387 |
| rs10066409 | -0.478345441 | 0.498501898 | 0.337273719 | 0.40889387 |
| rs1010334 | -0.496671427 | 0.497963175 | 0.318567511 | 0.40889387 |
| rs10189857 | -0.459867967 | 0.498962492 | 0.356712019 | 0.40889387 |
| rs10215082 | -0.479113794 | 0.498437332 | 0.336435651 | 0.40889387 |
| rs1050847 | -0.478615026 | 0.498456111 | 0.336957123 | 0.40889387 |
| rs10511592 | -0.530935817 | 0.495447588 | 0.283886871 | 0.40889387 |
| rs10518019 | -0.483326835 | 0.499077227 | 0.332824186 | 0.40889387 |
| rs10745789 | -0.477306502 | 0.498357459 | 0.338184135 | 0.40889387 |
| rs10760023 | -0.489883612 | 0.498211267 | 0.325467243 | 0.40889387 |
| rs10765775 | -0.468641749 | 0.499100074 | 0.347744345 | 0.40889387 |
| rs10844179 | -0.523156313 | 0.496074281 | 0.291611646 | 0.40889387 |
| rs10854884 | -0.482533958 | 0.499137781 | 0.333676531 | 0.40889387 |
| rs10994777 | -0.570207294 | 0.491649573 | 0.246136772 | 0.40889387 |
| rs11138947 | -0.497874483 | 0.497998244 | 0.31743079 | 0.40889387 |
| rs11155821 | -0.534193767 | 0.496122512 | 0.281597506 | 0.40889387 |
| rs11214468 | -0.468397795 | 0.498354671 | 0.347274787 | 0.40889387 |
| rs11243838 | -0.51367707 | 0.496933163 | 0.301279012 | 0.40889387 |
| rs11249939 | -0.462231186 | 0.498853287 | 0.354140783 | 0.40889387 |
| rs11572842 | -0.453938072 | 0.497243176 | 0.361290085 | 0.40889387 |
| rs115877304 | -0.531609531 | 0.495129196 | 0.282966838 | 0.40889387 |
| rs11604034 | -0.533278234 | 0.495917573 | 0.282224131 | 0.40889387 |
| rs11635966 | -0.456639843 | 0.497934692 | 0.359107145 | 0.40889387 |
| rs11661305 | -0.458222657 | 0.498141644 | 0.357643738 | 0.40889387 |
| rs11678980 | -0.513364821 | 0.498907867 | 0.303490388 | 0.40889387 |
| rs11690224 | -0.480781987 | 0.498388047 | 0.334708126 | 0.40889387 |
| rs11693764 | -0.479575571 | 0.498391588 | 0.335925786 | 0.40889387 |
| rs11714679 | -0.496286082 | 0.498038102 | 0.319015933 | 0.40889387 |
| rs11720121 | -0.493361283 | 0.498866187 | 0.322680183 | 0.40889387 |
| rs11732657 | -0.435707287 | 0.495436603 | 0.379162182 | 0.40889387 |
| rs11736863 | -0.5216021 | 0.497330152 | 0.294268107 | 0.40889387 |
| rs11764590 | -0.479693998 | 0.498707403 | 0.336112585 | 0.40889387 |
| rs117799466 | -0.481064548 | 0.498437406 | 0.334472011 | 0.40889387 |
| rs118083122 | -0.440665974 | 0.496381546 | 0.374671714 | 0.40889387 |
| rs11871429 | -0.475375894 | 0.498491845 | 0.340271862 | 0.40889387 |
| rs11915747 | -0.483132524 | 0.499397083 | 0.333328314 | 0.40889387 |
| rs12029988 | -0.462797115 | 0.498184401 | 0.352905932 | 0.40889387 |
| rs12076635 | -0.470277523 | 0.499625288 | 0.346571405 | 0.40889387 |
| rs12132451 | -0.481281131 | 0.498823066 | 0.334628288 | 0.40889387 |
| rs12468040 | -0.439093899 | 0.498052635 | 0.377981538 | 0.40889387 |
| rs12474895 | -0.510207649 | 0.497310979 | 0.304923257 | 0.40889387 |
| rs12503522 | -0.486270685 | 0.498281067 | 0.329115821 | 0.40889387 |
| rs12532494 | -0.445496035 | 0.498220435 | 0.371228505 | 0.40889387 |
| rs12574281 | -0.47669252 | 0.498359608 | 0.338807976 | 0.40889387 |
| rs12663818 | -0.416530282 | 0.492526185 | 0.397719117 | 0.413315946 |
| rs12735232 | -0.506018475 | 0.497739053 | 0.309327544 | 0.40889387 |
| rs12804787 | -0.524872569 | 0.495292891 | 0.289271279 | 0.40889387 |
| rs12921005 | -0.479446296 | 0.498350887 | 0.336016601 | 0.40889387 |
| rs12967855 | -0.533280113 | 0.499485097 | 0.28567404 | 0.40889387 |
| rs1334297 | -0.503131954 | 0.499761305 | 0.314057563 | 0.40889387 |
| rs13409451 | -0.48041227 | 0.499250436 | 0.33591545 | 0.40889387 |
| rs1363862 | -0.524782744 | 0.495340464 | 0.289400137 | 0.40889387 |
| rs1369128 | -0.520832795 | 0.496639634 | 0.294309901 | 0.40889387 |
| rs1381247 | -0.490080734 | 0.498203455 | 0.32526502 | 0.40889387 |
| rs1391438 | -0.495637205 | 0.499200183 | 0.320776904 | 0.40889387 |
| rs1452075 | -0.447701045 | 0.49688576 | 0.367580856 | 0.40889387 |
| rs145590108 | -0.506094449 | 0.497788694 | 0.30930316 | 0.40889387 |
| rs1566085 | -0.510303526 | 0.499085886 | 0.306555491 | 0.40889387 |
| rs1569266 | -0.533119942 | 0.495062859 | 0.281536902 | 0.40889387 |
| rs1620977 | -0.503961449 | 0.499442426 | 0.312951549 | 0.40889387 |
| rs1689510 | -0.510416994 | 0.498277085 | 0.305663487 | 0.40889387 |
| rs17489649 | -0.433029769 | 0.495382458 | 0.382046252 | 0.40889387 |
| rs17513684 | -0.465793508 | 0.498153751 | 0.349767867 | 0.40889387 |
| rs175325 | -0.452577077 | 0.497524848 | 0.363003305 | 0.40889387 |
| rs17563464 | -0.430696068 | 0.496614454 | 0.385797123 | 0.40894495 |
| rs17628095 | -0.49861818 | 0.498098888 | 0.316806238 | 0.40889387 |
| rs1788783 | -0.453661194 | 0.498191797 | 0.362497978 | 0.40889387 |
| rs1812587 | -0.473433589 | 0.498369493 | 0.342130022 | 0.40889387 |
| rs1835340 | -0.48897459 | 0.498268799 | 0.326421654 | 0.40889387 |
| rs185291 | -0.410432308 | 0.498437742 | 0.410259269 | 0.416148158 |
| rs1869165 | -0.488106195 | 0.498302483 | 0.327314245 | 0.40889387 |
| rs1880692 | -0.508706956 | 0.497173247 | 0.306213968 | 0.40889387 |
| rs1892417 | -0.440002894 | 0.497201154 | 0.376178465 | 0.40889387 |
| rs1917008 | -0.480101435 | 0.498356815 | 0.33536245 | 0.40889387 |
| rs192436652 | -0.532342294 | 0.495245727 | 0.282417095 | 0.40889387 |
| rs1964927 | -0.453619461 | 0.497727888 | 0.362094957 | 0.40889387 |
| rs1980251 | -0.517803545 | 0.498751106 | 0.299176816 | 0.40889387 |
| rs2145265 | -0.462175692 | 0.497909543 | 0.353287113 | 0.40889387 |
| rs215632 | -0.450627359 | 0.497160183 | 0.364722716 | 0.40889387 |
| rs2175420 | -0.478538322 | 0.498519197 | 0.337095707 | 0.40889387 |
| rs2182398 | -0.482230657 | 0.498337431 | 0.333204719 | 0.40889387 |
| rs2190872 | -0.493182213 | 0.498140552 | 0.322151486 | 0.40889387 |
| rs2287838 | -0.459961267 | 0.497768605 | 0.355462179 | 0.40889387 |
| rs2299098 | -0.492501419 | 0.498971205 | 0.323626094 | 0.40889387 |
| rs2309812 | -0.482360996 | 0.499844934 | 0.334534155 | 0.40889387 |
| rs2332818 | -0.461661282 | 0.497832497 | 0.353748682 | 0.40889387 |
| rs2411453 | -0.442279208 | 0.498379766 | 0.374845221 | 0.40889387 |
| rs2559509 | -0.490684379 | 0.498392701 | 0.3248532 | 0.40889387 |
| rs2570497 | -0.481109371 | 0.498653046 | 0.334636043 | 0.40889387 |
| rs2604541 | -0.488934655 | 0.498238858 | 0.326432094 | 0.40889387 |
| rs2706762 | -0.519328757 | 0.496598248 | 0.295666112 | 0.40889387 |
| rs2725371 | -0.479056683 | 0.49882382 | 0.336867769 | 0.40889387 |
| rs2735421 | -0.400734134 | 0.495690538 | 0.418839567 | 0.422828516 |
| rs281324 | -0.468375217 | 0.498193264 | 0.34714184 | 0.40889387 |
| rs2820313 | -0.462269126 | 0.497919563 | 0.353199489 | 0.40889387 |
| rs2834011 | -0.480465811 | 0.498461085 | 0.335096898 | 0.40889387 |
| rs2974312 | -0.442750254 | 0.497441845 | 0.373436729 | 0.40889387 |
| rs2998309 | -0.53462646 | 0.49377051 | 0.278922605 | 0.40889387 |
| rs324801 | -0.484048478 | 0.498380922 | 0.331427789 | 0.40889387 |
| rs333078 | -0.430970957 | 0.494868892 | 0.383820189 | 0.40889387 |
| rs34042385 | -0.455263768 | 0.497438534 | 0.36007818 | 0.40889387 |
| rs34192341 | -0.524161135 | 0.495958922 | 0.290573655 | 0.40889387 |
| rs34364916 | -0.425400957 | 0.493908331 | 0.389075387 | 0.41036807 |
| rs34470581 | -0.45935572 | 0.498197981 | 0.356510587 | 0.40889387 |
| rs34945223 | -0.442286683 | 0.496300309 | 0.372839342 | 0.40889387 |
| rs35039375 | -0.508766402 | 0.497726785 | 0.30669568 | 0.40889387 |
| rs35091253 | -0.485854116 | 0.498993294 | 0.330221099 | 0.40889387 |
| rs35811586 | -0.472242488 | 0.498248601 | 0.343228763 | 0.40889387 |
| rs35917528 | -0.468226721 | 0.498224995 | 0.347325441 | 0.40889387 |
| rs35999162 | -0.344351053 | 0.497164219 | 0.488541526 | 0.488541526 |
| rs363096 | -0.460109713 | 0.498186733 | 0.355710876 | 0.40889387 |
| rs3747631 | -0.533485439 | 0.497971311 | 0.284026641 | 0.40889387 |
| rs3788556 | -0.503420026 | 0.498100036 | 0.312169341 | 0.40889387 |
| rs3794620 | -0.477209231 | 0.498600309 | 0.338517922 | 0.40889387 |
| rs3800925 | -0.466096645 | 0.498708227 | 0.349990409 | 0.40889387 |
| rs3825083 | -0.497757843 | 0.498273865 | 0.317811946 | 0.40889387 |
| rs3827531 | -0.470366547 | 0.498211496 | 0.345113346 | 0.40889387 |
| rs3847225 | -0.477762117 | 0.49957039 | 0.338897485 | 0.40889387 |
| rs3943093 | -0.504101522 | 0.498719574 | 0.312116218 | 0.40889387 |
| rs4130477 | -0.523149015 | 0.495585456 | 0.291142693 | 0.40889387 |
| rs4146675 | -0.488290502 | 0.498257554 | 0.327088002 | 0.40889387 |
| rs417968 | -0.500254279 | 0.499005351 | 0.316100798 | 0.40889387 |
| rs42210 | -0.457631425 | 0.497595476 | 0.357737047 | 0.40889387 |
| rs4246167 | -0.411704037 | 0.49436397 | 0.404960133 | 0.414741779 |
| rs4700393 | -0.47129015 | 0.501067593 | 0.346924234 | 0.40889387 |
| rs4726070 | -0.443896282 | 0.49717947 | 0.371948718 | 0.40889387 |
| rs4731992 | -0.547863207 | 0.4960219 | 0.269370169 | 0.40889387 |
| rs4757957 | -0.478051538 | 0.498572881 | 0.337639439 | 0.40889387 |
| rs4780563 | -0.521512397 | 0.496120404 | 0.293175417 | 0.40889387 |
| rs4808766 | -0.525961699 | 0.495143727 | 0.288126538 | 0.40889387 |
| rs4958568 | -0.527228147 | 0.495623792 | 0.287434345 | 0.40889387 |
| rs55800473 | -0.475577791 | 0.498563942 | 0.340136653 | 0.40889387 |
| rs55842281 | -0.47423939 | 0.498503252 | 0.341438622 | 0.40889387 |
| rs55859553 | -0.532685192 | 0.494567118 | 0.281447381 | 0.40889387 |
| rs55872852 | -0.493161192 | 0.498140278 | 0.322171846 | 0.40889387 |
| rs56118554 | -0.552988932 | 0.495133 | 0.264058776 | 0.40889387 |
| rs575113 | -0.45159396 | 0.497106729 | 0.363643332 | 0.40889387 |
| rs59123361 | -0.423956888 | 0.495742623 | 0.392442897 | 0.41163661 |
| rs6071573 | -0.494026884 | 0.498593675 | 0.321763393 | 0.40889387 |
| rs613872 | -0.471859534 | 0.498811503 | 0.344165098 | 0.40889387 |
| rs61787087 | -0.496922157 | 0.497888477 | 0.31825067 | 0.40889387 |
| rs61787785 | -0.443704306 | 0.497068912 | 0.372049223 | 0.40889387 |
| rs61868084 | -0.473978448 | 0.498403319 | 0.341607542 | 0.40889387 |
| rs62018215 | -0.439783593 | 0.495884171 | 0.3751502 | 0.40889387 |
| rs62182125 | -0.465142713 | 0.497978305 | 0.350271807 | 0.40889387 |
| rs62184483 | -0.489675624 | 0.49928673 | 0.326715879 | 0.40889387 |
| rs62253608 | -0.531000293 | 0.495391657 | 0.28377404 | 0.40889387 |
| rs62389638 | -0.463753276 | 0.498551101 | 0.35226646 | 0.40889387 |
| rs6429911 | -0.445698918 | 0.49713422 | 0.369966355 | 0.40889387 |
| rs6556982 | -0.482150761 | 0.498342185 | 0.333289432 | 0.40889387 |
| rs660001 | -0.361510657 | 0.49161079 | 0.462120589 | 0.464310734 |
| rs6682095 | -0.488473733 | 0.498644752 | 0.327282284 | 0.40889387 |
| rs66844142 | -0.470789794 | 0.498230243 | 0.344697596 | 0.40889387 |
| rs6760772 | -0.465675458 | 0.498070197 | 0.349809176 | 0.40889387 |
| rs67651814 | -0.408823408 | 0.493044922 | 0.407002078 | 0.414829041 |
| rs6779254 | -0.433654517 | 0.496651674 | 0.382577913 | 0.40889387 |
| rs6789699 | -0.451284156 | 0.497477904 | 0.364330588 | 0.40889387 |
| rs67944653 | -0.510221943 | 0.497299182 | 0.304898236 | 0.40889387 |
| rs6935954 | -0.497275948 | 0.500383122 | 0.320324915 | 0.40889387 |
| rs6959579 | -0.470249265 | 0.498226352 | 0.345248026 | 0.40889387 |
| rs702606 | -0.437891176 | 0.49586987 | 0.37719478 | 0.40889387 |
| rs7031698 | -0.459681094 | 0.497786798 | 0.355772876 | 0.40889387 |
| rs7070693 | -0.468045794 | 0.498912961 | 0.348177011 | 0.40889387 |
| rs711793 | -0.480349474 | 0.498405812 | 0.335160341 | 0.40889387 |
| rs71646142 | -0.472802436 | 0.4983593 | 0.342764059 | 0.40889387 |
| rs7195278 | -0.488954089 | 0.498788887 | 0.326946619 | 0.40889387 |
| rs7233920 | -0.454542292 | 0.498143113 | 0.361519616 | 0.40889387 |
| rs72674898 | -0.552001471 | 0.491293195 | 0.261196201 | 0.40889387 |
| rs72807818 | -0.447654562 | 0.497092967 | 0.367830314 | 0.40889387 |
| rs72828517 | -0.512437432 | 0.49848631 | 0.303955956 | 0.40889387 |
| rs72977992 | -0.499506042 | 0.497813249 | 0.315667684 | 0.40889387 |
| rs73040036 | -0.513532355 | 0.496788908 | 0.30127487 | 0.40889387 |
| rs73499064 | -0.496224144 | 0.498231805 | 0.319264513 | 0.40889387 |
| rs75033012 | -0.452952091 | 0.497667266 | 0.362743167 | 0.40889387 |
| rs7526112 | -0.49506879 | 0.49872272 | 0.32086913 | 0.40889387 |
| rs7531271 | -0.42361568 | 0.497146179 | 0.394161471 | 0.41163661 |
| rs75433564 | -0.499911069 | 0.498019911 | 0.3154763 | 0.40889387 |
| rs7548936 | -0.414763978 | 0.496471532 | 0.403479312 | 0.414741779 |
| rs7580304 | -0.447655824 | 0.496665813 | 0.36741714 | 0.40889387 |
| rs7583473 | -0.472986318 | 0.498476637 | 0.342690008 | 0.40889387 |
| rs7598246 | -0.547477895 | 0.493412156 | 0.26718212 | 0.40889387 |
| rs7629643 | -0.513396517 | 0.496740954 | 0.30135609 | 0.40889387 |
| rs76608582 | -0.520126726 | 0.496601556 | 0.29492789 | 0.40889387 |
| rs7675394 | -0.455615637 | 0.498574137 | 0.36080235 | 0.40889387 |
| rs76878669 | -0.460658702 | 0.497889202 | 0.354849753 | 0.40889387 |
| rs77025239 | -0.478566954 | 0.49844191 | 0.336991889 | 0.40889387 |
| rs7758776 | -0.481551562 | 0.498467779 | 0.334012372 | 0.40889387 |
| rs77675579 | -0.411454846 | 0.492839617 | 0.403793381 | 0.414741779 |
| rs7768116 | -0.523555444 | 0.495802345 | 0.290979118 | 0.40889387 |
| rs781289 | -0.493309094 | 0.498556961 | 0.322431341 | 0.40889387 |
| rs78452560 | -0.436481342 | 0.496597745 | 0.379431931 | 0.40889387 |
| rs7868164 | -0.490724482 | 0.498159109 | 0.32458684 | 0.40889387 |
| rs7868984 | -0.513605573 | 0.500705611 | 0.305003044 | 0.40889387 |
| rs7873964 | -0.447488408 | 0.497230513 | 0.368140644 | 0.40889387 |
| rs7966054 | -0.449541331 | 0.49727421 | 0.365989595 | 0.40889387 |
| rs7977614 | -0.480373346 | 0.498528474 | 0.335255251 | 0.40889387 |
| rs7987170 | -0.519732272 | 0.496512155 | 0.295207303 | 0.40889387 |
| rs7988201 | -0.48293028 | 0.498603718 | 0.332762103 | 0.40889387 |
| rs7988627 | -0.476642659 | 0.4984615 | 0.338957243 | 0.40889387 |
| rs79937071 | -0.456412801 | 0.497576088 | 0.358999928 | 0.40889387 |
| rs8008382 | -0.46153597 | 0.497914603 | 0.353958722 | 0.40889387 |
| rs8020034 | -0.521189858 | 0.497289762 | 0.29461064 | 0.40889387 |
| rs8057808 | -0.532225752 | 0.495532586 | 0.282801196 | 0.40889387 |
| rs807478 | -0.485808265 | 0.498376233 | 0.329668324 | 0.40889387 |
| rs837065 | -0.505032651 | 0.498232931 | 0.310750902 | 0.40889387 |
| rs868698 | -0.519815237 | 0.496819962 | 0.295429504 | 0.40889387 |
| rs879394 | -0.471835916 | 0.498284162 | 0.343678852 | 0.40889387 |
| rs9372625 | -0.480983135 | 0.500925646 | 0.336960262 | 0.40889387 |
| rs9643120 | -0.472151375 | 0.498570393 | 0.343633451 | 0.40889387 |
| rs9797233 | -0.455957997 | 0.497450227 | 0.359357429 | 0.40889387 |
| rs9888796 | -0.531715237 | 0.495379428 | 0.283114356 | 0.40889387 |
| All | -0.480439664 | 0.496359491 | 0.333080919 | 0.40889387 |
