# Supplementary material for: Causal roles of educational duration in bone mineral density and risk factors for osteoporosis: a Mendelian randomization study
Source: BMC Musculoskelet Disord. 2024 May 2;25:345. doi: 10.1186/s12891-024-07428-8 (PMC11064366; doi:10.1186/s12891-024-07428-8)
Supplement: Supplementary file 1 — Supplementary Material 1. [file 12891_2024_7428_MOESM1_ESM.zip › IVs of Educational attainment on time for moderate physical activity.docx]

| SNP | b | se | P.value | adjust P.value |
| --- | --- | --- | --- | --- |
| rs10058365 | -0.002111567 | 0.009442923 | 0.823057872 | 0.970516319 |
| rs10066409 | -0.001346525 | 0.009406222 | 0.886169662 | 0.970516319 |
| rs1010334 | -0.001721425 | 0.009423447 | 0.855053273 | 0.970516319 |
| rs10189857 | -0.00254963 | 0.009431775 | 0.786911404 | 0.970516319 |
| rs10215082 | -0.001670479 | 0.009423617 | 0.859300151 | 0.970516319 |
| rs1050847 | -0.002309588 | 0.009421058 | 0.806339012 | 0.970516319 |
| rs10511592 | -0.001242244 | 0.009398684 | 0.89484812 | 0.970516319 |
| rs10518019 | -0.001791205 | 0.009440435 | 0.849514813 | 0.970516319 |
| rs10745789 | -0.002373357 | 0.009414666 | 0.800970391 | 0.970516319 |
| rs10760023 | -0.00081399 | 0.009323883 | 0.93043175 | 0.970516319 |
| rs10765775 | -0.000768404 | 0.009389701 | 0.934778079 | 0.970516319 |
| rs10844179 | -0.001829837 | 0.009428701 | 0.84612034 | 0.970516319 |
| rs10854884 | -0.001643824 | 0.009438774 | 0.861742486 | 0.970516319 |
| rs10994777 | -0.002407189 | 0.009426708 | 0.798446286 | 0.970516319 |
| rs11138947 | -0.00235273 | 0.009417423 | 0.802720874 | 0.970516319 |
| rs11155821 | -0.001972359 | 0.009436689 | 0.834440687 | 0.970516319 |
| rs11214468 | -0.001783642 | 0.009429189 | 0.849966033 | 0.970516319 |
| rs11243838 | -0.001207765 | 0.009389132 | 0.897646934 | 0.970516319 |
| rs11249939 | -0.002361147 | 0.009435309 | 0.80239722 | 0.970516319 |
| rs11572842 | -0.001414977 | 0.009403426 | 0.880390126 | 0.970516319 |
| rs115877304 | -0.002542863 | 0.009406846 | 0.786913717 | 0.970516319 |
| rs11604034 | -0.001530905 | 0.009425366 | 0.870972056 | 0.970516319 |
| rs11635966 | -0.002620247 | 0.009405208 | 0.780555379 | 0.970516319 |
| rs11661305 | -0.003381311 | 0.009318318 | 0.716704193 | 0.970516319 |
| rs11678980 | -0.001922853 | 0.009450421 | 0.838769736 | 0.970516319 |
| rs11690224 | -0.001219157 | 0.009387608 | 0.896670299 | 0.970516319 |
| rs11693764 | -0.002251804 | 0.009421893 | 0.811107885 | 0.970516319 |
| rs11714679 | -0.001981995 | 0.00942842 | 0.833499942 | 0.970516319 |
| rs11720121 | -0.001203569 | 0.009415453 | 0.89828419 | 0.970516319 |
| rs11732657 | -0.002558811 | 0.009399805 | 0.785453143 | 0.970516319 |
| rs11736863 | -0.00135823 | 0.009420371 | 0.88535827 | 0.970516319 |
| rs11764590 | -0.002476119 | 0.009419646 | 0.792652684 | 0.970516319 |
| rs117799466 | -0.002074813 | 0.009428309 | 0.825822797 | 0.970516319 |
| rs118083122 | -0.001794608 | 0.009427936 | 0.849034864 | 0.970516319 |
| rs11871429 | -0.001972162 | 0.009430916 | 0.83435715 | 0.970516319 |
| rs11915747 | -0.003122236 | 0.00940097 | 0.73979978 | 0.970516319 |
| rs12029988 | -0.0016697 | 0.009426406 | 0.859406223 | 0.970516319 |
| rs12076635 | -0.003439192 | 0.009387388 | 0.714094236 | 0.970516319 |
| rs12132451 | -0.002266178 | 0.009431834 | 0.810121643 | 0.970516319 |
| rs12468040 | -0.000387633 | 0.009353175 | 0.966941932 | 0.971524595 |
| rs12474895 | -0.002249128 | 0.009423901 | 0.811367596 | 0.970516319 |
| rs12503522 | -0.002411834 | 0.009410557 | 0.797726735 | 0.970516319 |
| rs12532494 | -0.000861603 | 0.009394947 | 0.926929055 | 0.970516319 |
| rs12574281 | -0.001498082 | 0.009412354 | 0.873541871 | 0.970516319 |
| rs12663818 | -0.001923499 | 0.009428246 | 0.838342167 | 0.970516319 |
| rs12735232 | -0.001924205 | 0.009431062 | 0.83833124 | 0.970516319 |
| rs12804787 | -0.002154235 | 0.009424013 | 0.819187639 | 0.970516319 |
| rs12921005 | -0.002979712 | 0.009345015 | 0.749835896 | 0.970516319 |
| rs12967855 | -0.00382635 | 0.009396709 | 0.683860333 | 0.970516319 |
| rs1334297 | -0.000923895 | 0.009431718 | 0.921967117 | 0.970516319 |
| rs13409451 | -0.00181765 | 0.009443991 | 0.847376977 | 0.970516319 |
| rs1363862 | -0.002121625 | 0.009425137 | 0.821899284 | 0.970516319 |
| rs1369128 | -0.002826847 | 0.009384316 | 0.763238354 | 0.970516319 |
| rs1381247 | -0.002234523 | 0.009421332 | 0.81251947 | 0.970516319 |
| rs1391438 | -0.001800869 | 0.009445803 | 0.848797641 | 0.970516319 |
| rs1452075 | -0.001630014 | 0.009420668 | 0.86263153 | 0.970516319 |
| rs145590108 | -0.00228655 | 0.009424869 | 0.808308955 | 0.970516319 |
| rs1566085 | -0.00067275 | 0.009400671 | 0.942948817 | 0.970516319 |
| rs1569266 | -0.001355294 | 0.009407136 | 0.885444534 | 0.970516319 |
| rs1620977 | -0.001268063 | 0.009440422 | 0.893147419 | 0.970516319 |
| rs1689510 | -0.001456566 | 0.009429432 | 0.877239037 | 0.970516319 |
| rs17489649 | -0.001963257 | 0.009429186 | 0.835064448 | 0.970516319 |
| rs17513684 | -0.001704665 | 0.009424935 | 0.856471505 | 0.970516319 |
| rs175325 | -0.002295867 | 0.009422793 | 0.80750164 | 0.970516319 |
| rs17563464 | -0.002746556 | 0.009407314 | 0.770317457 | 0.970516319 |
| rs17628095 | -0.001365465 | 0.009407813 | 0.884599072 | 0.970516319 |
| rs1788783 | -0.002177681 | 0.009434725 | 0.817458129 | 0.970516319 |
| rs1812587 | -0.001182596 | 0.00938673 | 0.899743046 | 0.970516319 |
| rs1835340 | -0.001870844 | 0.009427571 | 0.84269774 | 0.970516319 |
| rs185291 | -0.002040732 | 0.009467405 | 0.829335832 | 0.970516319 |
| rs1869165 | -0.0018147 | 0.009426857 | 0.847348101 | 0.970516319 |
| rs1880692 | -0.001679738 | 0.009421832 | 0.858501914 | 0.970516319 |
| rs1892417 | -0.000461571 | 0.00932119 | 0.960506108 | 0.971524595 |
| rs1917008 | -0.002290586 | 0.009419017 | 0.807860264 | 0.970516319 |
| rs192436652 | -0.00305053 | 0.009353684 | 0.744324939 | 0.970516319 |
| rs1964927 | -0.001824853 | 0.009430762 | 0.846567343 | 0.970516319 |
| rs1980251 | -0.001732222 | 0.009449297 | 0.854548881 | 0.970516319 |
| rs2145265 | -0.001098865 | 0.009372898 | 0.906670998 | 0.970516319 |
| rs215632 | -0.002217834 | 0.009423568 | 0.813937287 | 0.970516319 |
| rs2175420 | -0.001356977 | 0.009407753 | 0.885310741 | 0.970516319 |
| rs2182398 | -0.001946129 | 0.009427506 | 0.836454288 | 0.970516319 |
| rs2190872 | -0.002087785 | 0.00942679 | 0.824723708 | 0.970516319 |
| rs2287838 | -0.003216362 | 0.009305925 | 0.729624427 | 0.970516319 |
| rs2299098 | -0.002293854 | 0.009436836 | 0.80794767 | 0.970516319 |
| rs2309812 | -0.002430347 | 0.009449758 | 0.797035034 | 0.970516319 |
| rs2332818 | -0.00199273 | 0.009427635 | 0.832597675 | 0.970516319 |
| rs2411453 | -0.000590217 | 0.009380859 | 0.94983246 | 0.970516319 |
| rs2559509 | -0.003056344 | 0.009351939 | 0.743808597 | 0.970516319 |
| rs2570497 | -0.001001411 | 0.009381284 | 0.914990814 | 0.970516319 |
| rs2604541 | -0.000555726 | 0.009271621 | 0.95220469 | 0.970516319 |
| rs2706762 | -0.001477444 | 0.009415338 | 0.875308783 | 0.970516319 |
| rs2725371 | -0.001205167 | 0.009408791 | 0.898078138 | 0.970516319 |
| rs2735421 | -0.002018038 | 0.009449936 | 0.830897671 | 0.970516319 |
| rs281324 | -0.001963402 | 0.009428558 | 0.835041686 | 0.970516319 |
| rs2820313 | -0.002158298 | 0.009425296 | 0.818876743 | 0.970516319 |
| rs2834011 | -0.002772115 | 0.009383512 | 0.767669994 | 0.970516319 |
| rs2974312 | -0.002572168 | 0.009416941 | 0.784743613 | 0.970516319 |
| rs2998309 | -0.002938591 | 0.009347915 | 0.753249189 | 0.970516319 |
| rs324801 | -0.002082595 | 0.009427386 | 0.825163243 | 0.970516319 |
| rs333078 | -0.00154235 | 0.009415375 | 0.869879253 | 0.970516319 |
| rs34042385 | -0.001909384 | 0.009427783 | 0.839504507 | 0.970516319 |
| rs34192341 | -0.002492757 | 0.009409809 | 0.791078406 | 0.970516319 |
| rs34364916 | -0.001632853 | 0.009419864 | 0.862383016 | 0.970516319 |
| rs34470581 | -0.002270534 | 0.00942814 | 0.809690596 | 0.970516319 |
| rs34945223 | -0.00171569 | 0.009423965 | 0.85553876 | 0.970516319 |
| rs35039375 | -0.001634809 | 0.009426745 | 0.862319374 | 0.970516319 |
| rs35091253 | -0.001849011 | 0.009439794 | 0.84470869 | 0.970516319 |
| rs35811586 | -0.001546824 | 0.009414487 | 0.869492975 | 0.970516319 |
| rs35917528 | -0.0023036 | 0.009420412 | 0.806818134 | 0.970516319 |
| rs35999162 | -0.001717935 | 0.009497589 | 0.856460827 | 0.970516319 |
| rs363096 | -0.001494642 | 0.009420854 | 0.873942641 | 0.970516319 |
| rs3747631 | -0.003754262 | 0.009351566 | 0.688082908 | 0.970516319 |
| rs3788556 | -0.001771829 | 0.00943172 | 0.850987589 | 0.970516319 |
| rs3794620 | -0.001201055 | 0.009398797 | 0.898316629 | 0.970516319 |
| rs3800925 | -0.002937568 | 0.009392394 | 0.754462808 | 0.970516319 |
| rs3825083 | -0.00201095 | 0.009432412 | 0.831174326 | 0.970516319 |
| rs3827531 | -0.002161396 | 0.00942456 | 0.81860736 | 0.970516319 |
| rs3847225 | -0.002182085 | 0.009449219 | 0.817371098 | 0.970516319 |
| rs3943093 | -0.002570212 | 0.009427585 | 0.785140151 | 0.970516319 |
| rs4130477 | -0.001769818 | 0.009424741 | 0.851045803 | 0.970516319 |
| rs4146675 | -0.001741286 | 0.009424043 | 0.853409001 | 0.970516319 |
| rs417968 | -0.000651397 | 0.009382795 | 0.944651606 | 0.970516319 |
| rs42210 | -0.002019289 | 0.009427536 | 0.830398438 | 0.970516319 |
| rs4246167 | -0.001785758 | 0.009435142 | 0.849883825 | 0.970516319 |
| rs4700393 | -0.004493856 | 0.009363703 | 0.631282197 | 0.970516319 |
| rs4726070 | -0.002251233 | 0.009428105 | 0.811277011 | 0.970516319 |
| rs4731992 | -0.000440378 | 0.009358499 | 0.962468197 | 0.971524595 |
| rs4757957 | -0.001814957 | 0.009430795 | 0.847389668 | 0.970516319 |
| rs4780563 | -0.002548496 | 0.009403678 | 0.786382932 | 0.970516319 |
| rs4808766 | -0.001759809 | 0.009424138 | 0.851869034 | 0.970516319 |
| rs4958568 | -0.001962166 | 0.009429912 | 0.835167338 | 0.970516319 |
| rs55800473 | -0.001717851 | 0.009428807 | 0.855432266 | 0.970516319 |
| rs55842281 | -0.001174919 | 0.009392943 | 0.900456039 | 0.970516319 |
| rs55859553 | -0.001294014 | 0.00939624 | 0.890464793 | 0.970516319 |
| rs55872852 | -0.00204329 | 0.009427524 | 0.828413597 | 0.970516319 |
| rs56118554 | -0.002701148 | 0.009420138 | 0.774310002 | 0.970516319 |
| rs575113 | -0.001601733 | 0.009417786 | 0.864950855 | 0.970516319 |
| rs59123361 | -0.001710072 | 0.009433129 | 0.856144978 | 0.970516319 |
| rs6071573 | -0.002030075 | 0.009435385 | 0.829646057 | 0.970516319 |
| rs613872 | -0.002256499 | 0.009433327 | 0.810946661 | 0.970516319 |
| rs61787087 | -0.001953658 | 0.009426954 | 0.835821087 | 0.970516319 |
| rs61787785 | -0.001490004 | 0.00941944 | 0.874311842 | 0.970516319 |
| rs61868084 | -0.001982208 | 0.009429654 | 0.833503701 | 0.970516319 |
| rs62018215 | -0.002489771 | 0.009405155 | 0.791222014 | 0.970516319 |
| rs62182125 | -0.001818012 | 0.009425842 | 0.847056676 | 0.970516319 |
| rs62184483 | -0.001283099 | 0.009430528 | 0.891775448 | 0.970516319 |
| rs62253608 | -0.001913607 | 0.009430867 | 0.839206253 | 0.970516319 |
| rs62389638 | -0.002735428 | 0.009406006 | 0.771191133 | 0.970516319 |
| rs6429911 | -0.003318388 | 0.009315018 | 0.721660382 | 0.970516319 |
| rs6556982 | -0.001408168 | 0.009403921 | 0.880967669 | 0.970516319 |
| rs660001 | -0.002204481 | 0.009432869 | 0.815216325 | 0.970516319 |
| rs6682095 | -0.0024301 | 0.009422238 | 0.796475472 | 0.970516319 |
| rs66844142 | -0.001924559 | 0.009427892 | 0.838248348 | 0.970516319 |
| rs6760772 | -0.00127751 | 0.009393466 | 0.891821382 | 0.970516319 |
| rs67651814 | -0.00278019 | 0.00939413 | 0.767268521 | 0.970516319 |
| rs6779254 | -0.001453076 | 0.009422855 | 0.877446016 | 0.970516319 |
| rs6789699 | -0.002729743 | 0.009391935 | 0.771320902 | 0.970516319 |
| rs67944653 | -0.001605321 | 0.009421283 | 0.864701039 | 0.970516319 |
| rs6935954 | -0.001273143 | 0.009458301 | 0.892923476 | 0.970516319 |
| rs6959579 | -0.001391655 | 0.009403924 | 0.882353276 | 0.970516319 |
| rs702606 | -0.001430581 | 0.009408335 | 0.879143871 | 0.970516319 |
| rs7031698 | -0.001822285 | 0.009427188 | 0.846723225 | 0.970516319 |
| rs7070693 | -0.002341829 | 0.009434293 | 0.803960194 | 0.970516319 |
| rs711793 | -0.001897776 | 0.009428473 | 0.840478574 | 0.970516319 |
| rs71646142 | -0.002201844 | 0.009425003 | 0.815282067 | 0.970516319 |
| rs7195278 | -0.002586017 | 0.009417959 | 0.783636142 | 0.970516319 |
| rs7233920 | -0.002564888 | 0.009417031 | 0.785339891 | 0.970516319 |
| rs72674898 | -0.001692667 | 0.009423818 | 0.857454199 | 0.970516319 |
| rs72807818 | -0.002319662 | 0.009421028 | 0.805510614 | 0.970516319 |
| rs72828517 | -0.000228492 | 0.009330623 | 0.980463016 | 0.980463016 |
| rs72977992 | -0.002001399 | 0.009427543 | 0.83187864 | 0.970516319 |
| rs73040036 | -0.002702177 | 0.0093854 | 0.773413487 | 0.970516319 |
| rs73499064 | -0.001464275 | 0.009415686 | 0.87641581 | 0.970516319 |
| rs75033012 | -0.002437622 | 0.009417097 | 0.795750416 | 0.970516319 |
| rs7526112 | -0.00136855 | 0.009422291 | 0.884516634 | 0.970516319 |
| rs7531271 | -0.00228291 | 0.009442289 | 0.808954491 | 0.970516319 |
| rs75433564 | -0.003252477 | 0.009317843 | 0.727045044 | 0.970516319 |
| rs7548936 | -0.00192567 | 0.009446509 | 0.838470769 | 0.970516319 |
| rs7580304 | -0.00261042 | 0.009392153 | 0.781061252 | 0.970516319 |
| rs7583473 | -0.001945095 | 0.00943131 | 0.836604954 | 0.970516319 |
| rs7598246 | -0.00238667 | 0.009420919 | 0.800007612 | 0.970516319 |
| rs7629643 | -0.002264765 | 0.009420214 | 0.810008238 | 0.970516319 |
| rs76608582 | -0.002641952 | 0.009400542 | 0.778677832 | 0.970516319 |
| rs7675394 | -0.001206692 | 0.009417679 | 0.898045687 | 0.970516319 |
| rs76878669 | -0.002775941 | 0.009380345 | 0.767282303 | 0.970516319 |
| rs77025239 | -0.001621261 | 0.009421652 | 0.863376051 | 0.970516319 |
| rs7758776 | -0.002155623 | 0.009427165 | 0.819132571 | 0.970516319 |
| rs77675579 | -0.00109908 | 0.00938509 | 0.90677358 | 0.970516319 |
| rs7768116 | -0.003133253 | 0.009325729 | 0.736886361 | 0.970516319 |
| rs781289 | -0.003194759 | 0.009351938 | 0.732640905 | 0.970516319 |
| rs78452560 | -0.001854138 | 0.009433068 | 0.844174025 | 0.970516319 |
| rs7868164 | -0.002192461 | 0.009422546 | 0.816007972 | 0.970516319 |
| rs7868984 | -0.000593183 | 0.009445924 | 0.94992755 | 0.970516319 |
| rs7873964 | -0.001529901 | 0.009419728 | 0.870979401 | 0.970516319 |
| rs7966054 | -0.001612305 | 0.009422375 | 0.864133768 | 0.970516319 |
| rs7977614 | -0.001139483 | 0.009388087 | 0.903393717 | 0.970516319 |
| rs7987170 | -0.000897456 | 0.009354491 | 0.923569409 | 0.970516319 |
| rs7988201 | -0.000864436 | 0.009361618 | 0.926429214 | 0.970516319 |
| rs7988627 | -0.001741782 | 0.009427032 | 0.853413673 | 0.970516319 |
| rs79937071 | -0.00327686 | 0.009297064 | 0.724491974 | 0.970516319 |
| rs8008382 | -0.001909882 | 0.00942864 | 0.839477541 | 0.970516319 |
| rs8020034 | -0.001756286 | 0.009434886 | 0.852328628 | 0.970516319 |
| rs8057808 | -0.002632534 | 0.009404663 | 0.77954047 | 0.970516319 |
| rs807478 | -0.002085349 | 0.009427675 | 0.824941075 | 0.970516319 |
| rs837065 | -0.001804111 | 0.009435165 | 0.848359978 | 0.970516319 |
| rs868698 | -0.001959033 | 0.009432271 | 0.835467352 | 0.970516319 |
| rs879394 | -0.001873726 | 0.009427905 | 0.842464154 | 0.970516319 |
| rs9372625 | -0.002319965 | 0.009473887 | 0.806549346 | 0.970516319 |
| rs9643120 | -0.002137593 | 0.009431329 | 0.820697403 | 0.970516319 |
| rs9797233 | -0.002032816 | 0.009427118 | 0.829272339 | 0.970516319 |
| rs9888796 | -0.001951837 | 0.009431321 | 0.836046847 | 0.970516319 |
| All | -0.001955216 | 0.009390004 | 0.835054713 | 0.970516319 |
