# Supplementary material for: Causal roles of educational duration in bone mineral density and risk factors for osteoporosis: a Mendelian randomization study
Source: BMC Musculoskelet Disord. 2024 May 2;25:345. doi: 10.1186/s12891-024-07428-8 (PMC11064366; doi:10.1186/s12891-024-07428-8)
Supplement: Supplementary file 1 — Supplementary Material 1. [file 12891_2024_7428_MOESM1_ESM.zip › IVs of Educational attainment on time for vigorous physical activity.docx]

| SNP | b | se | P.value | adjust P.value |
| --- | --- | --- | --- | --- |
| rs10058365 | -0.045441805 | 0.008825465 | 2.62E-07 | 4.63E-07 |
| rs10066409 | -0.045387525 | 0.008812574 | 2.60E-07 | 4.63E-07 |
| rs1010334 | -0.044432439 | 0.00877113 | 4.07E-07 | 4.63E-07 |
| rs10189857 | -0.044738925 | 0.008823377 | 3.97E-07 | 4.63E-07 |
| rs10215082 | -0.044239476 | 0.008752964 | 4.32E-07 | 4.78E-07 |
| rs1050847 | -0.045361938 | 0.008812399 | 2.64E-07 | 4.63E-07 |
| rs10511592 | -0.044144636 | 0.008748366 | 4.51E-07 | 4.83E-07 |
| rs10518019 | -0.045591116 | 0.008818149 | 2.34E-07 | 4.63E-07 |
| rs10745789 | -0.045610905 | 0.008796576 | 2.16E-07 | 4.63E-07 |
| rs10760023 | -0.044610847 | 0.008789456 | 3.86E-07 | 4.63E-07 |
| rs10765775 | -0.044526067 | 0.008813158 | 4.37E-07 | 4.78E-07 |
| rs10844179 | -0.046032837 | 0.008757683 | 1.47E-07 | 4.63E-07 |
| rs10854884 | -0.046441472 | 0.00875638 | 1.13E-07 | 4.63E-07 |
| rs10994777 | -0.045869388 | 0.008794837 | 1.83E-07 | 4.63E-07 |
| rs11138947 | -0.045524327 | 0.008803948 | 2.33E-07 | 4.63E-07 |
| rs11155821 | -0.044753708 | 0.008814184 | 3.83E-07 | 4.63E-07 |
| rs11214468 | -0.045673259 | 0.008797994 | 2.09E-07 | 4.63E-07 |
| rs11243838 | -0.044577019 | 0.008790449 | 3.96E-07 | 4.63E-07 |
| rs11249939 | -0.044917558 | 0.008825007 | 3.58E-07 | 4.63E-07 |
| rs11572842 | -0.04496547 | 0.008810575 | 3.33E-07 | 4.63E-07 |
| rs115877304 | -0.044511479 | 0.008787081 | 4.07E-07 | 4.63E-07 |
| rs11604034 | -0.045408346 | 0.008816794 | 2.60E-07 | 4.63E-07 |
| rs11635966 | -0.044797471 | 0.008810136 | 3.68E-07 | 4.63E-07 |
| rs11661305 | -0.045665425 | 0.008803256 | 2.13E-07 | 4.63E-07 |
| rs11678980 | -0.044903721 | 0.008833202 | 3.71E-07 | 4.63E-07 |
| rs11690224 | -0.04510567 | 0.008814422 | 3.10E-07 | 4.63E-07 |
| rs11693764 | -0.045261907 | 0.00881357 | 2.81E-07 | 4.63E-07 |
| rs11714679 | -0.044390213 | 0.008768837 | 4.14E-07 | 4.65E-07 |
| rs11720121 | -0.044961794 | 0.008823852 | 3.48E-07 | 4.63E-07 |
| rs11732657 | -0.045594926 | 0.008797549 | 2.19E-07 | 4.63E-07 |
| rs11736863 | -0.04556465 | 0.008814074 | 2.35E-07 | 4.63E-07 |
| rs11764590 | -0.045082168 | 0.008819971 | 3.20E-07 | 4.63E-07 |
| rs117799466 | -0.045257968 | 0.008814491 | 2.83E-07 | 4.63E-07 |
| rs118083122 | -0.045059965 | 0.008815255 | 3.19E-07 | 4.63E-07 |
| rs11871429 | -0.045152717 | 0.00881694 | 3.04E-07 | 4.63E-07 |
| rs11915747 | -0.044850878 | 0.008829293 | 3.78E-07 | 4.63E-07 |
| rs12029988 | -0.045043454 | 0.008816778 | 3.24E-07 | 4.63E-07 |
| rs12076635 | -0.044424771 | 0.008820932 | 4.75E-07 | 5.01E-07 |
| rs12132451 | -0.045709318 | 0.008805463 | 2.09E-07 | 4.63E-07 |
| rs12468040 | -0.043137469 | 0.008668267 | 6.48E-07 | 6.51E-07 |
| rs12474895 | -0.044616803 | 0.008795253 | 3.92E-07 | 4.63E-07 |
| rs12503522 | -0.044533353 | 0.008781192 | 3.95E-07 | 4.63E-07 |
| rs12532494 | -0.044791273 | 0.008822474 | 3.84E-07 | 4.63E-07 |
| rs12574281 | -0.044914064 | 0.008810021 | 3.43E-07 | 4.63E-07 |
| rs12663818 | -0.044982681 | 0.008812335 | 3.32E-07 | 4.63E-07 |
| rs12735232 | -0.046112099 | 0.008752966 | 1.38E-07 | 4.63E-07 |
| rs12804787 | -0.04474202 | 0.008799287 | 3.68E-07 | 4.63E-07 |
| rs12921005 | -0.045600791 | 0.00879659 | 2.17E-07 | 4.63E-07 |
| rs12967855 | -0.045695229 | 0.008846735 | 2.40E-07 | 4.63E-07 |
| rs1334297 | -0.044141453 | 0.008815448 | 5.52E-07 | 5.68E-07 |
| rs13409451 | -0.043290486 | 0.008691838 | 6.34E-07 | 6.40E-07 |
| rs1363862 | -0.045516838 | 0.008801822 | 2.32E-07 | 4.63E-07 |
| rs1369128 | -0.045525565 | 0.008808086 | 2.36E-07 | 4.63E-07 |
| rs1381247 | -0.044420802 | 0.008768938 | 4.07E-07 | 4.63E-07 |
| rs1391438 | -0.045374084 | 0.008829684 | 2.76E-07 | 4.63E-07 |
| rs1452075 | -0.045409578 | 0.008809192 | 2.54E-07 | 4.63E-07 |
| rs145590108 | -0.045770304 | 0.008791737 | 1.93E-07 | 4.63E-07 |
| rs1566085 | -0.044896034 | 0.008834016 | 3.73E-07 | 4.63E-07 |
| rs1569266 | -0.044873171 | 0.008811516 | 3.53E-07 | 4.63E-07 |
| rs1620977 | -0.04573503 | 0.008827856 | 2.21E-07 | 4.63E-07 |
| rs1689510 | -0.045065612 | 0.008825283 | 3.28E-07 | 4.63E-07 |
| rs17489649 | -0.045778483 | 0.008784725 | 1.88E-07 | 4.63E-07 |
| rs17513684 | -0.045257911 | 0.008814545 | 2.83E-07 | 4.63E-07 |
| rs175325 | -0.045916589 | 0.008774356 | 1.67E-07 | 4.63E-07 |
| rs17563464 | -0.045520934 | 0.008815796 | 2.42E-07 | 4.63E-07 |
| rs17628095 | -0.045153164 | 0.008816697 | 3.03E-07 | 4.63E-07 |
| rs1788783 | -0.045189594 | 0.008822634 | 3.02E-07 | 4.63E-07 |
| rs1812587 | -0.045156332 | 0.008815385 | 3.02E-07 | 4.63E-07 |
| rs1835340 | -0.045112507 | 0.008814211 | 3.09E-07 | 4.63E-07 |
| rs185291 | -0.044400795 | 0.008838237 | 5.07E-07 | 5.29E-07 |
| rs1869165 | -0.044671237 | 0.008796312 | 3.81E-07 | 4.63E-07 |
| rs1880692 | -0.044732602 | 0.00879965 | 3.71E-07 | 4.63E-07 |
| rs1892417 | -0.044687363 | 0.008810035 | 3.93E-07 | 4.63E-07 |
| rs1917008 | -0.045203289 | 0.008813735 | 2.92E-07 | 4.63E-07 |
| rs192436652 | -0.045015021 | 0.008815886 | 3.29E-07 | 4.63E-07 |
| rs1964927 | -0.044967424 | 0.008815642 | 3.38E-07 | 4.63E-07 |
| rs1980251 | -0.045220492 | 0.00883538 | 3.09E-07 | 4.63E-07 |
| rs2145265 | -0.044747928 | 0.008801688 | 3.70E-07 | 4.63E-07 |
| rs215632 | -0.045944747 | 0.008764362 | 1.59E-07 | 4.63E-07 |
| rs2175420 | -0.045107324 | 0.008816844 | 3.12E-07 | 4.63E-07 |
| rs2182398 | -0.04500917 | 0.00881214 | 3.26E-07 | 4.63E-07 |
| rs2190872 | -0.04612941 | 0.008735114 | 1.29E-07 | 4.63E-07 |
| rs2287838 | -0.045243035 | 0.008813552 | 2.85E-07 | 4.63E-07 |
| rs2299098 | -0.044533864 | 0.008810336 | 4.31E-07 | 4.78E-07 |
| rs2309812 | -0.045843537 | 0.00882603 | 2.06E-07 | 4.63E-07 |
| rs2332818 | -0.045518062 | 0.0088024 | 2.33E-07 | 4.63E-07 |
| rs2411453 | -0.04349847 | 0.008728879 | 6.25E-07 | 6.34E-07 |
| rs2559509 | -0.04566339 | 0.008798359 | 2.10E-07 | 4.63E-07 |
| rs2570497 | -0.044592694 | 0.008800485 | 4.04E-07 | 4.63E-07 |
| rs2604541 | -0.045084633 | 0.008813499 | 3.13E-07 | 4.63E-07 |
| rs2706762 | -0.045102618 | 0.00881639 | 3.12E-07 | 4.63E-07 |
| rs2725371 | -0.044383693 | 0.008791456 | 4.45E-07 | 4.81E-07 |
| rs2735421 | -0.045172733 | 0.0088348 | 3.17E-07 | 4.63E-07 |
| rs281324 | -0.045352594 | 0.008811349 | 2.65E-07 | 4.63E-07 |
| rs2820313 | -0.045436484 | 0.00880781 | 2.49E-07 | 4.63E-07 |
| rs2834011 | -0.045887447 | 0.008775152 | 1.70E-07 | 4.63E-07 |
| rs2974312 | -0.045748848 | 0.008802231 | 2.02E-07 | 4.63E-07 |
| rs2998309 | -0.045347742 | 0.00880984 | 2.64E-07 | 4.63E-07 |
| rs324801 | -0.045339808 | 0.008811787 | 2.67E-07 | 4.63E-07 |
| rs333078 | -0.044892093 | 0.008809216 | 3.47E-07 | 4.63E-07 |
| rs34042385 | -0.046360507 | 0.008691584 | 9.61E-08 | 4.63E-07 |
| rs34192341 | -0.04527209 | 0.008814696 | 2.81E-07 | 4.63E-07 |
| rs34364916 | -0.045259779 | 0.008813052 | 2.81E-07 | 4.63E-07 |
| rs34470581 | -0.045861003 | 0.008788798 | 1.81E-07 | 4.63E-07 |
| rs34945223 | -0.044571153 | 0.008787702 | 3.94E-07 | 4.63E-07 |
| rs35039375 | -0.045143025 | 0.008818673 | 3.07E-07 | 4.63E-07 |
| rs35091253 | -0.045082503 | 0.008825488 | 3.25E-07 | 4.63E-07 |
| rs35811586 | -0.044411934 | 0.008768268 | 4.08E-07 | 4.63E-07 |
| rs35917528 | -0.045584215 | 0.008800532 | 2.22E-07 | 4.63E-07 |
| rs35999162 | -0.043733425 | 0.008850074 | 7.75E-07 | 7.75E-07 |
| rs363096 | -0.045096373 | 0.008818844 | 3.16E-07 | 4.63E-07 |
| rs3747631 | -0.046180601 | 0.00880095 | 1.54E-07 | 4.63E-07 |
| rs3788556 | -0.044605373 | 0.008801727 | 4.02E-07 | 4.63E-07 |
| rs3794620 | -0.044169397 | 0.008757693 | 4.57E-07 | 4.85E-07 |
| rs3800925 | -0.045783071 | 0.008803041 | 1.98E-07 | 4.63E-07 |
| rs3825083 | -0.044830354 | 0.008812144 | 3.63E-07 | 4.63E-07 |
| rs3827531 | -0.045684497 | 0.008789998 | 2.02E-07 | 4.63E-07 |
| rs3847225 | -0.045921244 | 0.008815465 | 1.90E-07 | 4.63E-07 |
| rs3943093 | -0.045327573 | 0.008826467 | 2.82E-07 | 4.63E-07 |
| rs4130477 | -0.044742195 | 0.008799779 | 3.69E-07 | 4.63E-07 |
| rs4146675 | -0.045377587 | 0.008809371 | 2.59E-07 | 4.63E-07 |
| rs417968 | -0.043676324 | 0.00874529 | 5.91E-07 | 6.02E-07 |
| rs42210 | -0.044857827 | 0.008807138 | 3.52E-07 | 4.63E-07 |
| rs4246167 | -0.046087913 | 0.008775018 | 1.50E-07 | 4.63E-07 |
| rs4700393 | -0.047600212 | 0.008746591 | 5.26E-08 | 4.63E-07 |
| rs4726070 | -0.044338401 | 0.008778584 | 4.40E-07 | 4.78E-07 |
| rs4731992 | -0.043799701 | 0.008756947 | 5.68E-07 | 5.82E-07 |
| rs4757957 | -0.04535135 | 0.00881519 | 2.68E-07 | 4.63E-07 |
| rs4780563 | -0.046232398 | 0.008723878 | 1.16E-07 | 4.63E-07 |
| rs4808766 | -0.045483093 | 0.00880366 | 2.39E-07 | 4.63E-07 |
| rs4958568 | -0.045680802 | 0.008795045 | 2.06E-07 | 4.63E-07 |
| rs55800473 | -0.045507032 | 0.008809718 | 2.40E-07 | 4.63E-07 |
| rs55842281 | -0.044201901 | 0.008757 | 4.47E-07 | 4.81E-07 |
| rs55859553 | -0.044786617 | 0.008804534 | 3.64E-07 | 4.63E-07 |
| rs55872852 | -0.044493488 | 0.008779401 | 4.02E-07 | 4.63E-07 |
| rs56118554 | -0.045961234 | 0.008799237 | 1.76E-07 | 4.63E-07 |
| rs575113 | -0.044769658 | 0.00880188 | 3.65E-07 | 4.63E-07 |
| rs59123361 | -0.045214193 | 0.008821582 | 2.97E-07 | 4.63E-07 |
| rs6071573 | -0.045627035 | 0.008808632 | 2.22E-07 | 4.63E-07 |
| rs613872 | -0.045797085 | 0.008801668 | 1.96E-07 | 4.63E-07 |
| rs61787087 | -0.045015397 | 0.008811703 | 3.25E-07 | 4.63E-07 |
| rs61787785 | -0.044742648 | 0.008807867 | 3.78E-07 | 4.63E-07 |
| rs61868084 | -0.045455196 | 0.008808719 | 2.47E-07 | 4.63E-07 |
| rs62018215 | -0.044756782 | 0.008801089 | 3.67E-07 | 4.63E-07 |
| rs62182125 | -0.044520978 | 0.00877993 | 3.96E-07 | 4.63E-07 |
| rs62184483 | -0.045170269 | 0.008831431 | 3.14E-07 | 4.63E-07 |
| rs62253608 | -0.045006611 | 0.00881563 | 3.30E-07 | 4.63E-07 |
| rs62389638 | -0.045853173 | 0.008795385 | 1.85E-07 | 4.63E-07 |
| rs6429911 | -0.046428828 | 0.008707205 | 9.70E-08 | 4.63E-07 |
| rs6556982 | -0.044412267 | 0.008768147 | 4.08E-07 | 4.63E-07 |
| rs660001 | -0.045701482 | 0.008804957 | 2.10E-07 | 4.63E-07 |
| rs6682095 | -0.046265024 | 0.008747289 | 1.23E-07 | 4.63E-07 |
| rs66844142 | -0.045612821 | 0.008796206 | 2.15E-07 | 4.63E-07 |
| rs6760772 | -0.0451519 | 0.008814447 | 3.02E-07 | 4.63E-07 |
| rs67651814 | -0.044969603 | 0.008817225 | 3.39E-07 | 4.63E-07 |
| rs6779254 | -0.043775354 | 0.008717744 | 5.13E-07 | 5.33E-07 |
| rs6789699 | -0.045987848 | 0.008767689 | 1.56E-07 | 4.63E-07 |
| rs67944653 | -0.045039202 | 0.008814987 | 3.23E-07 | 4.63E-07 |
| rs6935954 | -0.044858451 | 0.008849966 | 4.00E-07 | 4.63E-07 |
| rs6959579 | -0.044820222 | 0.008805661 | 3.58E-07 | 4.63E-07 |
| rs702606 | -0.045051454 | 0.008814109 | 3.20E-07 | 4.63E-07 |
| rs7031698 | -0.044504504 | 0.008781751 | 4.02E-07 | 4.63E-07 |
| rs7070693 | -0.04556109 | 0.008818206 | 2.38E-07 | 4.63E-07 |
| rs711793 | -0.045207763 | 0.008814572 | 2.92E-07 | 4.63E-07 |
| rs71646142 | -0.044913883 | 0.008811323 | 3.45E-07 | 4.63E-07 |
| rs7195278 | -0.045706295 | 0.008806596 | 2.10E-07 | 4.63E-07 |
| rs7233920 | -0.04440736 | 0.008791854 | 4.40E-07 | 4.78E-07 |
| rs72674898 | -0.044699093 | 0.008799356 | 3.78E-07 | 4.63E-07 |
| rs72807818 | -0.045568705 | 0.008803155 | 2.26E-07 | 4.63E-07 |
| rs72828517 | -0.044313609 | 0.008801139 | 4.78E-07 | 5.02E-07 |
| rs72977992 | -0.044837595 | 0.008805921 | 3.55E-07 | 4.63E-07 |
| rs73040036 | -0.044840893 | 0.008806671 | 3.55E-07 | 4.63E-07 |
| rs73499064 | -0.044747371 | 0.008806207 | 3.75E-07 | 4.63E-07 |
| rs75033012 | -0.045235997 | 0.008817058 | 2.89E-07 | 4.63E-07 |
| rs7526112 | -0.04487529 | 0.008820145 | 3.62E-07 | 4.63E-07 |
| rs7531271 | -0.045805449 | 0.008814583 | 2.03E-07 | 4.63E-07 |
| rs75433564 | -0.045283021 | 0.008815156 | 2.79E-07 | 4.63E-07 |
| rs7548936 | -0.044466153 | 0.008814111 | 4.54E-07 | 4.83E-07 |
| rs7580304 | -0.045097628 | 0.008813197 | 3.10E-07 | 4.63E-07 |
| rs7583473 | -0.04523481 | 0.008816769 | 2.89E-07 | 4.63E-07 |
| rs7598246 | -0.045399979 | 0.008813963 | 2.59E-07 | 4.63E-07 |
| rs7629643 | -0.044997397 | 0.008812076 | 3.28E-07 | 4.63E-07 |
| rs76608582 | -0.046502149 | 0.008689293 | 8.72E-08 | 4.63E-07 |
| rs7675394 | -0.045715892 | 0.008811963 | 2.13E-07 | 4.63E-07 |
| rs76878669 | -0.044649196 | 0.008796079 | 3.85E-07 | 4.63E-07 |
| rs77025239 | -0.044606017 | 0.008793615 | 3.93E-07 | 4.63E-07 |
| rs7758776 | -0.045212354 | 0.008815673 | 2.92E-07 | 4.63E-07 |
| rs77675579 | -0.045129527 | 0.008817384 | 3.08E-07 | 4.63E-07 |
| rs7768116 | -0.045978185 | 0.008760072 | 1.53E-07 | 4.63E-07 |
| rs781289 | -0.046754039 | 0.008670905 | 6.97E-08 | 4.63E-07 |
| rs78452560 | -0.045401669 | 0.008815541 | 2.60E-07 | 4.63E-07 |
| rs7868164 | -0.045461279 | 0.008804747 | 2.43E-07 | 4.63E-07 |
| rs7868984 | -0.044326189 | 0.008849214 | 5.47E-07 | 5.66E-07 |
| rs7873964 | -0.044685514 | 0.008802753 | 3.85E-07 | 4.63E-07 |
| rs7966054 | -0.045285362 | 0.00881494 | 2.79E-07 | 4.63E-07 |
| rs7977614 | -0.044272774 | 0.008764301 | 4.38E-07 | 4.78E-07 |
| rs7987170 | -0.044686765 | 0.008801006 | 3.83E-07 | 4.63E-07 |
| rs7988201 | -0.046065451 | 0.00876464 | 1.47E-07 | 4.63E-07 |
| rs7988627 | -0.044882268 | 0.008811116 | 3.51E-07 | 4.63E-07 |
| rs79937071 | -0.04543661 | 0.008807907 | 2.49E-07 | 4.63E-07 |
| rs8008382 | -0.045281437 | 0.008813511 | 2.78E-07 | 4.63E-07 |
| rs8020034 | -0.045483226 | 0.008816494 | 2.48E-07 | 4.63E-07 |
| rs8057808 | -0.045885872 | 0.008782966 | 1.75E-07 | 4.63E-07 |
| rs807478 | -0.044673928 | 0.008797655 | 3.82E-07 | 4.63E-07 |
| rs837065 | -0.045572406 | 0.008812229 | 2.32E-07 | 4.63E-07 |
| rs868698 | -0.045357489 | 0.008815317 | 2.67E-07 | 4.63E-07 |
| rs879394 | -0.044812799 | 0.008805636 | 3.60E-07 | 4.63E-07 |
| rs9372625 | -0.045311003 | 0.008858888 | 3.14E-07 | 4.63E-07 |
| rs9643120 | -0.04452735 | 0.008795467 | 4.14E-07 | 4.65E-07 |
| rs9797233 | -0.045136328 | 0.008813811 | 3.04E-07 | 4.63E-07 |
| rs9888796 | -0.045743109 | 0.008793127 | 1.97E-07 | 4.63E-07 |
| All | -0.045146503 | 0.008778675 | 2.71E-07 | 4.63E-07 |
