# Supplementary material for: Causal roles of educational duration in bone mineral density and risk factors for osteoporosis: a Mendelian randomization study
Source: BMC Musculoskelet Disord. 2024 May 2;25:345. doi: 10.1186/s12891-024-07428-8 (PMC11064366; doi:10.1186/s12891-024-07428-8)
Supplement: Supplementary file 1 — Supplementary Material 1. [file 12891_2024_7428_MOESM1_ESM.zip › IVs of Educational attainment on whole body fat mass.docx]

| SNP | b | se | P.value | adjust P.value |
| --- | --- | --- | --- | --- |
| rs10058365 | -0.051091931 | 0.006094385 | 5.14E-17 | 5.48E-17 |
| rs10066409 | -0.051368459 | 0.006091974 | 3.39E-17 | 5.20E-17 |
| rs1010334 | -0.051598678 | 0.006086574 | 2.30E-17 | 5.20E-17 |
| rs10189857 | -0.051261953 | 0.006100036 | 4.33E-17 | 5.26E-17 |
| rs10215082 | -0.052074061 | 0.00604368 | 6.92E-18 | 5.20E-17 |
| rs1050847 | -0.05195898 | 0.006059365 | 9.91E-18 | 5.20E-17 |
| rs10511592 | -0.051197323 | 0.006087846 | 4.11E-17 | 5.25E-17 |
| rs10518019 | -0.051724148 | 0.006093051 | 2.08E-17 | 5.20E-17 |
| rs10745789 | -0.051687266 | 0.006081626 | 1.91E-17 | 5.20E-17 |
| rs10760023 | -0.051425744 | 0.006090108 | 3.06E-17 | 5.20E-17 |
| rs10765775 | -0.051447755 | 0.00610054 | 3.36E-17 | 5.20E-17 |
| rs10844179 | -0.051218281 | 0.00608759 | 3.98E-17 | 5.20E-17 |
| rs10854884 | -0.052003899 | 0.006077998 | 1.17E-17 | 5.20E-17 |
| rs10994777 | -0.051350587 | 0.006095781 | 3.64E-17 | 5.20E-17 |
| rs11138947 | -0.0517554 | 0.006077907 | 1.66E-17 | 5.20E-17 |
| rs11155821 | -0.051715928 | 0.006089234 | 2.01E-17 | 5.20E-17 |
| rs11214468 | -0.051710053 | 0.006083708 | 1.90E-17 | 5.20E-17 |
| rs11243838 | -0.051533047 | 0.006089615 | 2.62E-17 | 5.20E-17 |
| rs11249939 | -0.050805661 | 0.006076243 | 6.20E-17 | 6.44E-17 |
| rs11572842 | -0.05126706 | 0.006087308 | 3.70E-17 | 5.20E-17 |
| rs115877304 | -0.052009335 | 0.006054315 | 8.66E-18 | 5.20E-17 |
| rs11604034 | -0.050908623 | 0.006073522 | 5.20E-17 | 5.50E-17 |
| rs11635966 | -0.051687893 | 0.006085816 | 2.01E-17 | 5.20E-17 |
| rs11661305 | -0.051507381 | 0.006093151 | 2.83E-17 | 5.20E-17 |
| rs11678980 | -0.051142097 | 0.006101593 | 5.21E-17 | 5.50E-17 |
| rs11690224 | -0.051290217 | 0.006089059 | 3.66E-17 | 5.20E-17 |
| rs11693764 | -0.051793632 | 0.006074207 | 1.50E-17 | 5.20E-17 |
| rs11714679 | -0.051211382 | 0.00608623 | 3.95E-17 | 5.20E-17 |
| rs11720121 | -0.051220111 | 0.006095773 | 4.37E-17 | 5.26E-17 |
| rs11732657 | -0.051191676 | 0.00608455 | 3.98E-17 | 5.20E-17 |
| rs11736863 | -0.05171178 | 0.006090082 | 2.05E-17 | 5.20E-17 |
| rs11764590 | -0.050972043 | 0.006077701 | 5.00E-17 | 5.45E-17 |
| rs117799466 | -0.051217048 | 0.006087 | 3.96E-17 | 5.20E-17 |
| rs118083122 | -0.051486375 | 0.006090772 | 2.83E-17 | 5.20E-17 |
| rs11871429 | -0.051260433 | 0.006089911 | 3.85E-17 | 5.20E-17 |
| rs11915747 | -0.05055109 | 0.006063909 | 7.66E-17 | 7.83E-17 |
| rs12029988 | -0.050993228 | 0.006075737 | 4.74E-17 | 5.33E-17 |
| rs12076635 | -0.051729463 | 0.006102136 | 2.31E-17 | 5.20E-17 |
| rs12132451 | -0.051841815 | 0.00608195 | 1.54E-17 | 5.20E-17 |
| rs12468040 | -0.051253671 | 0.00609995 | 4.38E-17 | 5.26E-17 |
| rs12474895 | -0.051319278 | 0.006090675 | 3.58E-17 | 5.20E-17 |
| rs12503522 | -0.051365323 | 0.00608975 | 3.32E-17 | 5.20E-17 |
| rs12532494 | -0.051581604 | 0.006098056 | 2.70E-17 | 5.20E-17 |
| rs12574281 | -0.051474171 | 0.006090164 | 2.86E-17 | 5.20E-17 |
| rs12663818 | -0.051085441 | 0.006078201 | 4.29E-17 | 5.26E-17 |
| rs12735232 | -0.051273386 | 0.00609065 | 3.82E-17 | 5.20E-17 |
| rs12804787 | -0.05133514 | 0.006089247 | 3.44E-17 | 5.20E-17 |
| rs12921005 | -0.051662213 | 0.006082684 | 2.01E-17 | 5.20E-17 |
| rs12967855 | -0.052003185 | 0.006106352 | 1.65E-17 | 5.20E-17 |
| rs1334297 | -0.050939188 | 0.006101652 | 6.92E-17 | 7.12E-17 |
| rs13409451 | -0.051706881 | 0.006096521 | 2.23E-17 | 5.20E-17 |
| rs1363862 | -0.051404934 | 0.006090116 | 3.15E-17 | 5.20E-17 |
| rs1369128 | -0.051118836 | 0.006084928 | 4.43E-17 | 5.26E-17 |
| rs1381247 | -0.051484694 | 0.006089464 | 2.80E-17 | 5.20E-17 |
| rs1391438 | -0.051697238 | 0.006097944 | 2.29E-17 | 5.20E-17 |
| rs1452075 | -0.052325916 | 0.00599457 | 2.57E-18 | 5.20E-17 |
| rs145590108 | -0.051522025 | 0.00609188 | 2.73E-17 | 5.20E-17 |
| rs1566085 | -0.050678562 | 0.006079484 | 7.68E-17 | 7.83E-17 |
| rs1569266 | -0.051470683 | 0.006091789 | 2.93E-17 | 5.20E-17 |
| rs1620977 | -0.053068444 | 0.005983294 | 7.35E-19 | 5.20E-17 |
| rs1689510 | -0.050936446 | 0.006083052 | 5.59E-17 | 5.87E-17 |
| rs17489649 | -0.051621045 | 0.00608655 | 2.23E-17 | 5.20E-17 |
| rs17513684 | -0.051198621 | 0.006086078 | 4.02E-17 | 5.20E-17 |
| rs175325 | -0.051521628 | 0.006090836 | 2.70E-17 | 5.20E-17 |
| rs17563464 | -0.05168672 | 0.006090952 | 2.14E-17 | 5.20E-17 |
| rs17628095 | -0.051149515 | 0.006085181 | 4.26E-17 | 5.26E-17 |
| rs1788783 | -0.049922823 | 0.005925957 | 3.63E-17 | 5.20E-17 |
| rs1812587 | -0.050795452 | 0.006048949 | 4.56E-17 | 5.32E-17 |
| rs1835340 | -0.051500075 | 0.006089694 | 2.75E-17 | 5.20E-17 |
| rs185291 | -0.053346722 | 0.005988855 | 5.21E-19 | 5.20E-17 |
| rs1869165 | -0.05160662 | 0.006086416 | 2.27E-17 | 5.20E-17 |
| rs1880692 | -0.051776521 | 0.006074235 | 1.54E-17 | 5.20E-17 |
| rs1892417 | -0.051557031 | 0.006094012 | 2.67E-17 | 5.20E-17 |
| rs1917008 | -0.051637442 | 0.006084147 | 2.12E-17 | 5.20E-17 |
| rs192436652 | -0.051256807 | 0.006089982 | 3.88E-17 | 5.20E-17 |
| rs1964927 | -0.050981066 | 0.00607467 | 4.76E-17 | 5.33E-17 |
| rs1980251 | -0.052016721 | 0.006087122 | 1.28E-17 | 5.20E-17 |
| rs2145265 | -0.051193112 | 0.00608492 | 3.99E-17 | 5.20E-17 |
| rs215632 | -0.050664921 | 0.00602661 | 4.21E-17 | 5.26E-17 |
| rs2175420 | -0.051288474 | 0.006090642 | 3.74E-17 | 5.20E-17 |
| rs2182398 | -0.051243474 | 0.006086546 | 3.79E-17 | 5.20E-17 |
| rs2190872 | -0.051618616 | 0.006085531 | 2.21E-17 | 5.20E-17 |
| rs2287838 | -0.051338785 | 0.006089799 | 3.45E-17 | 5.20E-17 |
| rs2299098 | -0.051220679 | 0.006096933 | 4.42E-17 | 5.26E-17 |
| rs2309812 | -0.050404723 | 0.006065017 | 9.51E-17 | 9.65E-17 |
| rs2332818 | -0.05106785 | 0.006075706 | 4.27E-17 | 5.26E-17 |
| rs2411453 | -0.049157051 | 0.005819776 | 3.00E-17 | 5.20E-17 |
| rs2559509 | -0.050961873 | 0.006071648 | 4.72E-17 | 5.33E-17 |
| rs2570497 | -0.051039104 | 0.006081446 | 4.76E-17 | 5.33E-17 |
| rs2604541 | -0.05111873 | 0.006079582 | 4.16E-17 | 5.26E-17 |
| rs2706762 | -0.051404912 | 0.006092081 | 3.23E-17 | 5.20E-17 |
| rs2725371 | -0.050646691 | 0.006051047 | 5.77E-17 | 6.02E-17 |
| rs2735421 | -0.051967317 | 0.006089459 | 1.41E-17 | 5.20E-17 |
| rs281324 | -0.051719495 | 0.006079967 | 1.79E-17 | 5.20E-17 |
| rs2820313 | -0.050406831 | 0.005974591 | 3.26E-17 | 5.20E-17 |
| rs2834011 | -0.05135183 | 0.006091059 | 3.44E-17 | 5.20E-17 |
| rs2974312 | -0.05130967 | 0.006094894 | 3.81E-17 | 5.20E-17 |
| rs2998309 | -0.051279608 | 0.006087583 | 3.65E-17 | 5.20E-17 |
| rs324801 | -0.051211798 | 0.006086063 | 3.94E-17 | 5.20E-17 |
| rs333078 | -0.051330661 | 0.006089912 | 3.49E-17 | 5.20E-17 |
| rs34042385 | -0.051455385 | 0.006090212 | 2.94E-17 | 5.20E-17 |
| rs34192341 | -0.05158557 | 0.006088171 | 2.39E-17 | 5.20E-17 |
| rs34364916 | -0.051247896 | 0.006087287 | 3.80E-17 | 5.20E-17 |
| rs34470581 | -0.051276672 | 0.006092466 | 3.88E-17 | 5.20E-17 |
| rs34945223 | -0.051318249 | 0.006089641 | 3.54E-17 | 5.20E-17 |
| rs35039375 | -0.051023595 | 0.006080359 | 4.80E-17 | 5.33E-17 |
| rs35091253 | -0.050709164 | 0.006065031 | 6.22E-17 | 6.44E-17 |
| rs35811586 | -0.05171482 | 0.00607948 | 1.79E-17 | 5.20E-17 |
| rs35917528 | -0.051494699 | 0.006090538 | 2.79E-17 | 5.20E-17 |
| rs35999162 | -0.050365911 | 0.00611184 | 1.71E-16 | 1.71E-16 |
| rs363096 | -0.051240481 | 0.006091192 | 4.02E-17 | 5.20E-17 |
| rs3747631 | -0.051443809 | 0.006105431 | 3.58E-17 | 5.20E-17 |
| rs3788556 | -0.051618663 | 0.006090269 | 2.34E-17 | 5.20E-17 |
| rs3794620 | -0.051366354 | 0.006093211 | 3.45E-17 | 5.20E-17 |
| rs3800925 | -0.051988054 | 0.006073179 | 1.13E-17 | 5.20E-17 |
| rs3825083 | -0.051653624 | 0.006088098 | 2.17E-17 | 5.20E-17 |
| rs3827531 | -0.051346121 | 0.006089804 | 3.41E-17 | 5.20E-17 |
| rs3847225 | -0.051484175 | 0.006104896 | 3.36E-17 | 5.20E-17 |
| rs3943093 | -0.051110043 | 0.006094427 | 5.01E-17 | 5.45E-17 |
| rs4130477 | -0.051467962 | 0.006089785 | 2.88E-17 | 5.20E-17 |
| rs4146675 | -0.051568144 | 0.006087339 | 2.43E-17 | 5.20E-17 |
| rs417968 | -0.052256143 | 0.006060955 | 6.59E-18 | 5.20E-17 |
| rs42210 | -0.051364158 | 0.006089985 | 3.33E-17 | 5.20E-17 |
| rs4246167 | -0.051120723 | 0.006089419 | 4.66E-17 | 5.33E-17 |
| rs4700393 | -0.051961156 | 0.006115492 | 1.95E-17 | 5.20E-17 |
| rs4726070 | -0.051427023 | 0.006093771 | 3.19E-17 | 5.20E-17 |
| rs4731992 | -0.051569267 | 0.006099892 | 2.81E-17 | 5.20E-17 |
| rs4757957 | -0.050984589 | 0.00607559 | 4.79E-17 | 5.33E-17 |
| rs4780563 | -0.050957201 | 0.006067807 | 4.54E-17 | 5.32E-17 |
| rs4808766 | -0.050479189 | 0.005980191 | 3.14E-17 | 5.20E-17 |
| rs4958568 | -0.051895972 | 0.006067368 | 1.20E-17 | 5.20E-17 |
| rs55800473 | -0.051631158 | 0.006088756 | 2.26E-17 | 5.20E-17 |
| rs55842281 | -0.051692401 | 0.00608495 | 1.98E-17 | 5.20E-17 |
| rs55859553 | -0.051516223 | 0.006089653 | 2.68E-17 | 5.20E-17 |
| rs55872852 | -0.051356676 | 0.006090264 | 3.38E-17 | 5.20E-17 |
| rs56118554 | -0.051866815 | 0.006086832 | 1.58E-17 | 5.20E-17 |
| rs575113 | -0.051749606 | 0.006076471 | 1.65E-17 | 5.20E-17 |
| rs59123361 | -0.051169025 | 0.006091557 | 4.47E-17 | 5.26E-17 |
| rs6071573 | -0.052238448 | 0.006040472 | 5.24E-18 | 5.20E-17 |
| rs613872 | -0.051045036 | 0.006086443 | 5.00E-17 | 5.45E-17 |
| rs61787087 | -0.051826664 | 0.006068086 | 1.33E-17 | 5.20E-17 |
| rs61787785 | -0.050736543 | 0.006051362 | 5.10E-17 | 5.46E-17 |
| rs61868084 | -0.051475739 | 0.00609114 | 2.89E-17 | 5.20E-17 |
| rs62018215 | -0.051440414 | 0.006090071 | 3.00E-17 | 5.20E-17 |
| rs62182125 | -0.051478348 | 0.0060896 | 2.83E-17 | 5.20E-17 |
| rs62184483 | -0.051506006 | 0.006102055 | 3.15E-17 | 5.20E-17 |
| rs62253608 | -0.051290762 | 0.006090907 | 3.74E-17 | 5.20E-17 |
| rs62389638 | -0.051795633 | 0.006084404 | 1.70E-17 | 5.20E-17 |
| rs6429911 | -0.051487945 | 0.006092391 | 2.88E-17 | 5.20E-17 |
| rs6556982 | -0.051710541 | 0.006079431 | 1.80E-17 | 5.20E-17 |
| rs660001 | -0.051134044 | 0.006089749 | 4.59E-17 | 5.32E-17 |
| rs6682095 | -0.051271977 | 0.006092943 | 3.93E-17 | 5.20E-17 |
| rs66844142 | -0.051609915 | 0.006085522 | 2.24E-17 | 5.20E-17 |
| rs6760772 | -0.051512895 | 0.006089518 | 2.69E-17 | 5.20E-17 |
| rs67651814 | -0.051261553 | 0.006091758 | 3.93E-17 | 5.20E-17 |
| rs6779254 | -0.051208321 | 0.006092202 | 4.26E-17 | 5.26E-17 |
| rs6789699 | -0.051284064 | 0.006090744 | 3.76E-17 | 5.20E-17 |
| rs67944653 | -0.051760912 | 0.006078605 | 1.66E-17 | 5.20E-17 |
| rs6935954 | -0.050170234 | 0.006065575 | 1.32E-16 | 1.34E-16 |
| rs6959579 | -0.051612339 | 0.006085847 | 2.24E-17 | 5.20E-17 |
| rs702606 | -0.051328998 | 0.00609013 | 3.51E-17 | 5.20E-17 |
| rs7031698 | -0.051378129 | 0.006090675 | 3.30E-17 | 5.20E-17 |
| rs7070693 | -0.051643138 | 0.006094851 | 2.39E-17 | 5.20E-17 |
| rs711793 | -0.05170867 | 0.006081104 | 1.84E-17 | 5.20E-17 |
| rs71646142 | -0.051671645 | 0.006083952 | 2.01E-17 | 5.20E-17 |
| rs7195278 | -0.051374319 | 0.006096283 | 3.54E-17 | 5.20E-17 |
| rs7233920 | -0.051683253 | 0.006089758 | 2.12E-17 | 5.20E-17 |
| rs72674898 | -0.051000659 | 0.006071488 | 4.46E-17 | 5.26E-17 |
| rs72807818 | -0.05140387 | 0.006091677 | 3.22E-17 | 5.20E-17 |
| rs72828517 | -0.052323337 | 0.006051648 | 5.33E-18 | 5.20E-17 |
| rs72977992 | -0.051286342 | 0.006088312 | 3.65E-17 | 5.20E-17 |
| rs73040036 | -0.051490344 | 0.006089889 | 2.79E-17 | 5.20E-17 |
| rs73499064 | -0.051609043 | 0.00608862 | 2.32E-17 | 5.20E-17 |
| rs75033012 | -0.051794351 | 0.006079121 | 1.59E-17 | 5.20E-17 |
| rs7526112 | -0.051354153 | 0.006097039 | 3.68E-17 | 5.20E-17 |
| rs7531271 | -0.052029484 | 0.006081183 | 1.17E-17 | 5.20E-17 |
| rs75433564 | -0.051510736 | 0.006091008 | 2.75E-17 | 5.20E-17 |
| rs7548936 | -0.051135943 | 0.006098326 | 5.06E-17 | 5.45E-17 |
| rs7580304 | -0.05127694 | 0.006087589 | 3.66E-17 | 5.20E-17 |
| rs7583473 | -0.050884345 | 0.006065037 | 4.87E-17 | 5.38E-17 |
| rs7598246 | -0.050793583 | 0.006057454 | 5.06E-17 | 5.45E-17 |
| rs7629643 | -0.051294182 | 0.006088643 | 3.62E-17 | 5.20E-17 |
| rs76608582 | -0.050895617 | 0.006064803 | 4.78E-17 | 5.33E-17 |
| rs7675394 | -0.051410308 | 0.006098965 | 3.48E-17 | 5.20E-17 |
| rs76878669 | -0.051387966 | 0.006090971 | 3.26E-17 | 5.20E-17 |
| rs77025239 | -0.051535109 | 0.006089871 | 2.62E-17 | 5.20E-17 |
| rs7758776 | -0.051227416 | 0.006088063 | 3.95E-17 | 5.20E-17 |
| rs77675579 | -0.051449438 | 0.006092422 | 3.05E-17 | 5.20E-17 |
| rs7768116 | -0.051559931 | 0.00608841 | 2.48E-17 | 5.20E-17 |
| rs781289 | -0.051496877 | 0.006094704 | 2.93E-17 | 5.20E-17 |
| rs78452560 | -0.051419037 | 0.006094423 | 3.25E-17 | 5.20E-17 |
| rs7868164 | -0.051577919 | 0.006086459 | 2.37E-17 | 5.20E-17 |
| rs7868984 | -0.053324328 | 0.006021181 | 8.28E-19 | 5.20E-17 |
| rs7873964 | -0.051513787 | 0.006091545 | 2.75E-17 | 5.20E-17 |
| rs7966054 | -0.051343543 | 0.00609138 | 3.49E-17 | 5.20E-17 |
| rs7977614 | -0.051771717 | 0.006079526 | 1.66E-17 | 5.20E-17 |
| rs7987170 | -0.051561183 | 0.006089666 | 2.52E-17 | 5.20E-17 |
| rs7988201 | -0.052075491 | 0.00605279 | 7.73E-18 | 5.20E-17 |
| rs7988627 | -0.05168048 | 0.0060843 | 2.00E-17 | 5.20E-17 |
| rs79937071 | -0.051170578 | 0.006084041 | 4.08E-17 | 5.24E-17 |
| rs8008382 | -0.051254443 | 0.006088107 | 3.80E-17 | 5.20E-17 |
| rs8020034 | -0.051184955 | 0.006092009 | 4.39E-17 | 5.26E-17 |
| rs8057808 | -0.052085299 | 0.006050575 | 7.41E-18 | 5.20E-17 |
| rs807478 | -0.051535446 | 0.006089272 | 2.60E-17 | 5.20E-17 |
| rs837065 | -0.05162456 | 0.006092244 | 2.37E-17 | 5.20E-17 |
| rs868698 | -0.051611955 | 0.006089288 | 2.33E-17 | 5.20E-17 |
| rs879394 | -0.051484699 | 0.006090182 | 2.82E-17 | 5.20E-17 |
| rs9372625 | -0.050138094 | 0.006074184 | 1.53E-16 | 1.54E-16 |
| rs9643120 | -0.052161019 | 0.006043914 | 6.12E-18 | 5.20E-17 |
| rs9797233 | -0.051375466 | 0.006090013 | 3.28E-17 | 5.20E-17 |
| rs9888796 | -0.051914153 | 0.006067765 | 1.17E-17 | 5.20E-17 |
| All | -0.051413402 | 0.006065914 | 2.33E-17 | 5.20E-17 |
